# Partially Biobased Polyurethanes With Reversibly Crosslinkable Furan Units Toward Recyclable Thermosetting Textile Fibres

**DOI:** 10.1002/chem.71541

**Published:** 2026-08-10

**Authors:** Rafael N. L. Menezes, Fredrik Bäcklund, Tam T. Nguyen, Maria Maukonen, Zengwei Guo, Olga Gordivska, Cecilia Hansson, Baozhong Zhang

**Affiliations:** ^1^ Centre For Analysis and Synthesis, Department of Chemistry Lund University Lund Sweden; ^2^ Division Materials and Production, Department of Polymers Fibers and Composites RISE Research Institutes of Sweden Mölndal Sweden; ^3^ Max IV Lund Sweden; ^4^ Bona AB Malmö Sweden

**Keywords:** biobased polymers, fibers, polyurethanes, recycling, textiles

## Abstract

Molecular design combining biobased feedstocks, fiber processability, and reversible crosslinking is important for sustainable textiles. Herein, we report a new molecular design and synthetic strategy for partially biobased polyurethanes with tuneable chemical composition and properties, which show potential for the development of reversibly crosslinked textile fibers with enhanced recyclability. A lignin‐derived new aromatic monomer was synthesized as the rigid building block, which could be copolymerized with flexible tetraethylene glycol and diisocyanates to achieve the balanced structural rigidity needed for fiber design. Moreover, a sugar‐based 2,5‐bis(hydroxymethyl)furan was also incorporated in the copolymerization, which offers enhanced structural rigidity and enables thermally induced crosslinking/decrosslinking based on Diels‐Alder chemistry. The resulting polyurethanes exhibited moderate to high molecular weights (10–280 kDa) and tuneable glass transition temperatures (31–124°C). Reversible crosslinking/decrosslinking of the obtained polymers could be conveniently carried out and repeated at least five cycles without significant property deterioration. Selected polymers were processed into noncrosslinked and crosslinked fibers by wet spinning at different draw ratios. The spinnability, thermal properties, and fiber mechanical responses were affected by molecular composition and processing conditions. These results demonstrate that the molecular design strategy successfully introduces thermally reversible crosslinks into partially biobased polyurethanes with potential for fiber applications.

## Graphical abstract

A molecular design strategy combines lignin‐derived aromatic units, sugar‐derived furan groups, and flexible glycol segments to create wet‐spinnable polyurethanes. Thermally reversible Diels–Alder crosslinking chemistry enables formation of crosslinked thermosetting fibers, repeated de‐crosslinking/re‐crosslinking cycles, and tunable fiber properties, highlighting a promising approach toward future development of recyclable crosslinked textile materials.

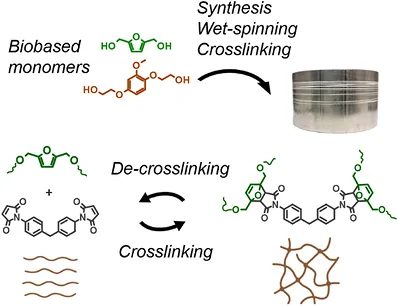

## 1 Introduction

In modern society, fibers are widely used in everyday applications such as clothing, medicine, and construction [1, 2, 3, 4, 5], with an annual production of more than 100 million of tons [4, 6, 7, 8, 9, 10]. Currently, fossil‐based synthetic fibers represent approximately 78% of the fiber market share [10]. However, growing concerns about the environmental impacts of fossil‐based materials have led to a renewed interest in biobased fibers. These biobased fibers can be either fully naturally produced (e.g., cotton, silk, etc.) or synthesized from biomass‐based chemical building blocks (e.g., PLA, PHA, etc.) [11, 12, 13]. While the former may be subjected to inherent natural constraints (e.g., seasonality of growing, weather conditions, land availability) [10], the latter can have the potential to promote the use of agricultural wastes and drive the development of new materials, technologies, and processes, contributing to sustainable industrial growth [11]. In addition, the use of biobased chemicals for fiber synthesis offers more control and diversity in terms of fiber composition, properties, and functionalities [11]. Therefore, there has been active research and development of versatile functional biobased polymer fibers toward various commodity or niche applications [14, 15, 16].

For textiles, strong and durable fibers are desirable because they generally increase the resistance to wear, tear, and damage of textiles, and can lead to longer‐lasting reliable products for various applications, from clothing to industrial gear [17]. In general, chemical crosslinking in fibers (i.e. thermosets) can enhance the mechanical strength and durability [15, 18, 19, 20, 21, 22, 23]. However, the current textile production is completely dominated by the use of noncrosslinked thermoplastic fibers (e.g., Elastane), while chemically crosslinked (e.g., via covalent bonding, hydrogen bonding, or ionic bonding) fibers have been only reported occasionally [24, 25]. A major hindrance to the development of chemically crosslinked fibers is their inherent challenges in recycling caused by the chemically fixed networks [26]. Therefore, reversibly crosslinked textile fibers have significant potential, because they may combine network stability during use with improved solubility, processability, and recyclability, enabled by de‐crosslinking [27]. By this means, the advantages of thermoplastics and thermosets can be combined [6]. During the past decade, several types of dynamic covalent chemistry have been investigated to achieve reversible crosslinking for various materials, for example those based on oxime‐urethane [20, 28, 29], disulfide [27, 30, 31], imine [32, 33, 34], thiourea [35], acyl hydrazone [36], and boronic ester [37]. However, some of these chemistries rely on the use of toxic or fossil‐based chemicals, which are undesirable for sustainable fibers and textiles. Among them, Diels‐Alder reactions using furan moieties (as dienes) are particularly attractive, since many furan derivatives can be conveniently synthesized from various biomass resources (e.g., polysaccharides) [38, 39, 40]. The underlying mechanisms and kinetics of reversible Diels‐Alder reactions of furan units have been extensively reported in the literature [41, 42]. However, the molecular design and synthesis of new reversibly crosslinked polymer fibers for textiles based on such chemistry has only been rarely investigated [18].

At the early stage of developing a new concept for recyclable textile materials, molecular design is fundamentally important, as the elastic polymers for textile fibers usually require the combination of soft and hard segments (Scheme 1) [43]. In the case of polyurethane fibers, the soft segments generally consist of aliphatic polyethers or polyesters [18, 44], providing flexibility, extensibility, and spinnability. The hard segments generally contain aromatic structures, which can contribute to rigidity, thermal stability, and mechanical performance. By tuning the balance between the hard and soft segments, fibers can be tailored to meet the specific mechanical, thermal, and functional requirements toward commodity textiles or high‐performance technical applications [45]. In addition to molecular design, solvent selection is also an important consideration for recyclable polymer and fiber systems. Therefore, the use of solvents with lower environmental, health, and safety impacts (e.g., according to the Chem21 solvent selection guide) is increasingly important [46]. Herein, we report the initial molecular design and synthesis of new polyurethanes using a lignin‐based rigid aromatic monomer **Q** (Scheme 1), a potentially biobased long flexible tetraethylene glycol (TEG) [47, 48, 49], and a sugar‐based 2,5‐bis(hydroxymethyl)furan as the crosslinkable unit. These monomers were polymerized with two different isocyanates in varied ratios to produce a series of thermoplastic polyurethanes with tunable structural composition and thermal properties. The obtained polymers could effectively undergo thermally controlled reversible crosslinking/decrosslinking via Diels‐Alder chemistry, which was indirectly investigated by different methods. The preliminary fiber spinnability of selected polymers was demonstrated, and the resulting fiber could be thermally de‐crosslinked without changing the molecular and thermal properties (Scheme 1). This proof‐of‐concept study suggests the potential of the new molecular design concept to realize reversible crosslinking and potentially facilitate the development of recyclable partially biobased textile fibers.

**SCHEME 1:**
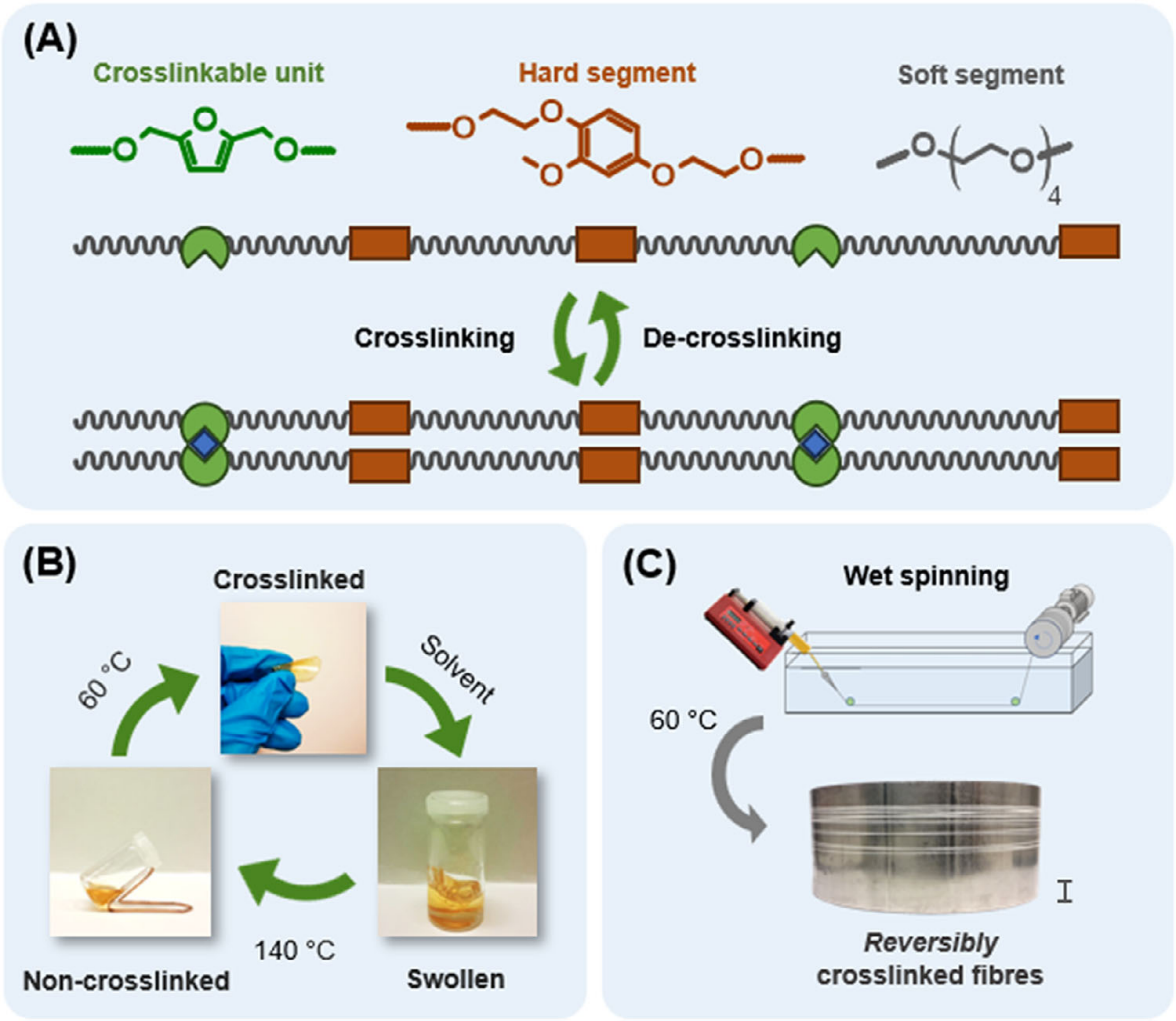
(A) Schematic representation of the production of polyurethanes with reversibly crosslinkable furan units (blue squares represent the crosslinker, bismaleimide), (B) reversible crosslinking/decrosslinking cycles of the obtained polymer, and (C) production of crosslinked fibers via wet‐spinning process (scale bar = 1 cm).

**SCHEME 2:**
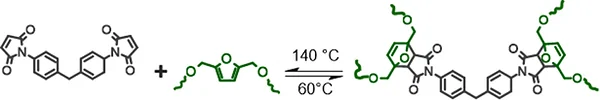
Reversible crosslinking of the obtained furan‐based polymer via Diels‐Alder and retro‐Diels‐Alder reactions.

## 2 Results and Discussion

### 2.1 Monomer Q Synthesis

Monomer **Q** was synthesized via the hydroxyethylation of 2‐methoxyhydroquinone (Figure 1), which converted the phenolic OH into primary OH groups to enhance reactivity [50]. Ethylene carbonate—a compound that could be produced from sustainable sources including CO_2_, ethylene glycol, and urea—was used for the hydroxyethylation [51]. This reaction required the use of polar solvents, so several solvents were screened, including dimethylformamide (DMF), dimethylsulfoxide (DMSO), cyrene, and ethylene carbonate (EC; in excess, as both solvent and reagent). The reaction conditions (140°C, 2 h, and K_2_CO_3_ used as the base catalyst) were chosen based on a previous study by our group, where an analogous hydroxyethylation reaction of a bisphenol was optimized [52]. As a result, DMF and DMSO proved to be the most effective solvents, enabling high conversion (86% and 80%, respectively, as determined from Figure S1 and Equation S1) and high yield (73% and 70%, respectively). The molecular structure of the obtained monomer **Q** was unambiguously characterized (Figures S2–S7). Unfortunately, cyrene was not suitable as a solvent for this synthesis. The proton nuclear magnetic resonance spectroscopy (^1^H NMR) spectrum of the purified product displayed a series of unidentified peaks at 4–8 ppm without the corresponding peaks for monomer **Q** (Figure S8). The use of ethylene carbonate as the solvent (neat reaction) yielded less straightforward results than the other reactions. After 2 h at 140°C (the same condition used for the other solvents), the purified product contained a mixture of monomer **Q** (∼64 mol%) and other diols structures (formed by the subsequent reaction of monomer **Q** and ethylene carbonate [53, 54]; Figures S9 and S10), which were difficult to separate by a straightforward precipitation, recrystallization, or liquid extraction. To better understand this reaction, we conducted a kinetic investigation (details in Supporting Information), revealing that in the neat synthesis the phenol groups of 2‐methoxyhydroquinone were completely consumed to form monomer **Q** within just 15 min. In this case, the removal of unreacted ethylene carbonate by precipitation, recrystallization, or liquid extraction was also challenging, as monomer **Q** and ethylene carbonate had similar solubility in different solvents (e.g., water, acetone, chloroform, ethyl acetate, methanol, ethanol, and anisole). Despite the presence of byproducts in the final product obtained by monomer **Q**’s synthesis at different times, the mixture of products obtained (monomer **Q** and ethylene carbonate, or monomer **Q** and diol mixture, for the syntheses carried out for 15 min and 2 h, respectively), could in principle still serve as a viable precursor for polyurethane synthesis. To investigate the contribution of monomer **Q** to the properties of polyurethanes, the remaining syntheses described in this paper were carried out with pure monomer **Q**.

**FIGURE 1:**
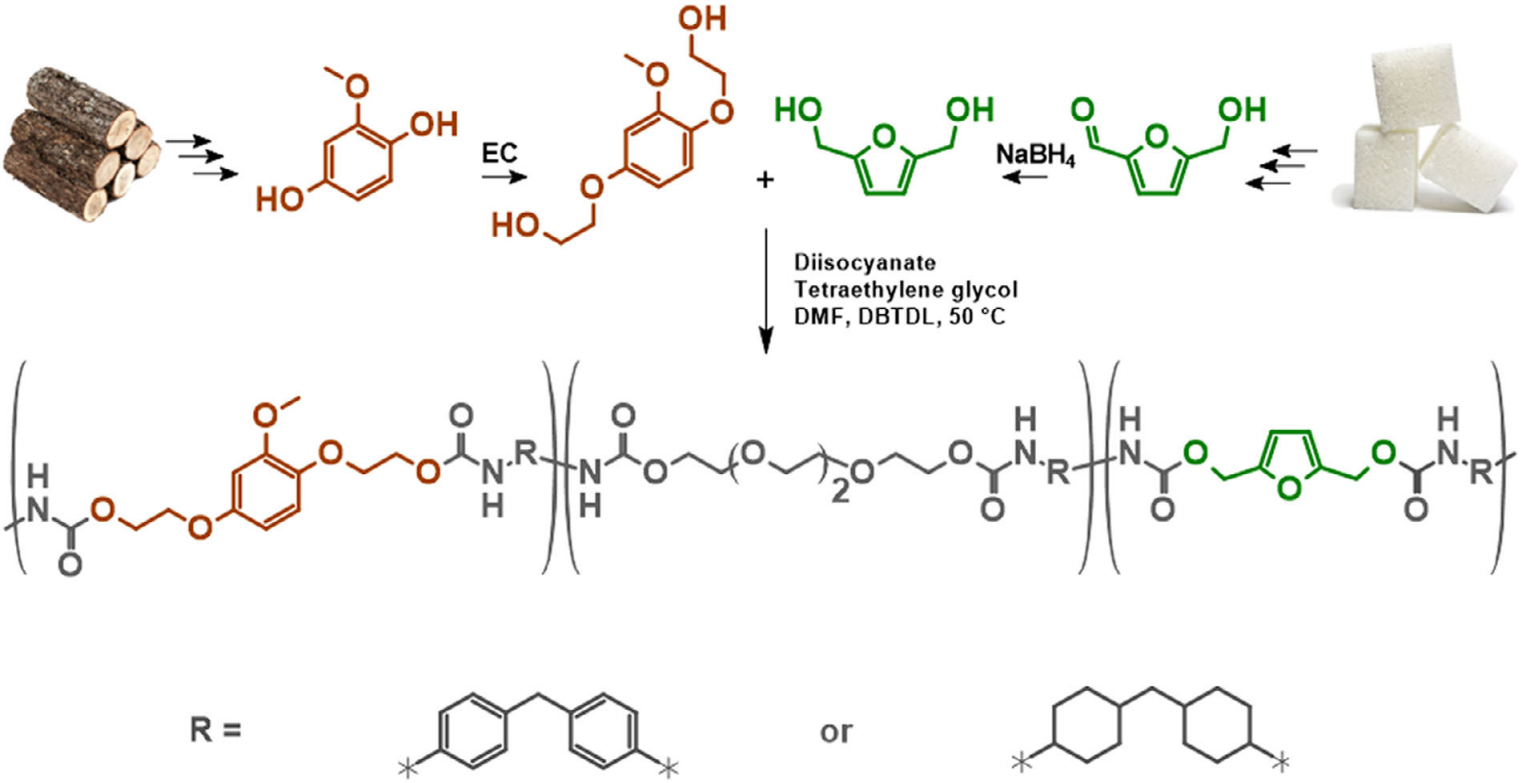
Synthesis of monomer **Q**, BHF, and the potentially biobased polyurethanes.

The sustainability of monomer **Q**’s synthesis was further evaluated in terms of e‐factor (detailed in Supporting Information), which is a metric used to quantify the amount of waste generated in a chemical process relative to the amount of product. For monomer **Q** synthesis, using DMF or DMSO as solvent (reactions with the highest yields), this value corresponded to 36.6 and 36.3, which is in the same range as some industrial small‐molecule synthesis [55]. The higher contribution to the waste mass in this process comes from purification, mainly extraction in chloroform. Considering that chloroform can be easily recovered by distillation and subsequently reused, the e‐factor can be hypothetically reduced to 3.45 in both cases. For **BHF**, optimized synthesis procedures presented in the literature show that it can be obtained with an e‐factor as low as 3.7 [56]. Overall, the synthetic route to obtain monomer **Q** and **BHF** had a similar E‐factor compared to other monomers used in the synthesis of reversible crosslinked fibers. For instance, 5‐[[4‐(hydroxymethyl)‐2‐methoxyphenoxy]methyl]‐2‐furanmethanol, a monomer obtained from 5‐chloromethylfurfural and vanillin, possessed a significantly higher e‐factor (350) [18]. Furfuryl methacrylate, another potentially biobased monomer used in reversible crosslinking processes [6], can be synthesized by different routes, with e‐factor ranging from 1.96 to 64.6 [57, 58, 59]. While this analysis provides an initial indication of the material efficiency of the monomer synthesis, a full environmental assessment of the polymer synthesis, fiber processing, and recycling steps will be required in future studies.

### 2.2 Polyurethane (PU) Synthesis

The reactivity of monomer **Q** with the two commonly used diisocyanates, 4,4‐methylenebis(phenyl isocyanate) and 4,4'‐methylenebis(cyclohexyl isocyanate) (**MDI** and **H_12_MDI**, respectively), was investigated. The reactions followed a protocol previously established by our group, in which the diol was reacted with 1.05 equivalents of diisocyanate (excess added to compensate for the residual moisture in the solvent) in dimethylacetamide (DMAc), using dibutyltin dilaurate catalyst [18, 44, 60]. The reaction was carried out until no isocyanate peak (2253 cm^−1^) was observed in the Fourier‐transform infrared (FTIR) spectrum of the reaction medium (typically within 4 h). Afterwards, the polymer was isolated by precipitation in MeOH. The copolymerization with **MDI** yielded **P(Q‐M)** with high molecular weight (M_n_∼280 kDa), whereas the copolymerization with **H_12_MDI** resulted in **P(Q‐H)** with decent but lower molecular weight (M_n_∼40 kDa). These were generally higher than the polyurethanes obtained by the polymerization of 2‐methoxyhydroquinone with aliphatic isocyanates under similar conditions (M_n_∼ 18–27 kDa) [61], which confirmed the high reactivity of the primary OH groups in monomer **Q** compared to phenolic OH groups. Furthermore, copolymerizations of monomer **Q**, **BHF** (crosslinkable unit), **TEG** (soft segment), and the diisocyanate (**MDI** or **H_12_MDI**) were also investigated. The reactions were carried out similarly, yielding copolymers with molecular weight ranging from 10 to 41 kDa (Figures S23–28). These copolymers, containing **TEG** and **BHF**, are referred to as **P(Q_a_T_b_F_c_‐X)**, where a, b, and c represent the molar percentage of each diol respective to the total diol content (determined from ^1^H‐NMR data, Supporting Information; summarized in Table 1) and X represents the diisocyanate used (either **MDI**, represented as **M**, or **H_12_MDI**, represented as **H**). Overall, the polymer composition was rather consistent with the monomer feed (Table 1). Because polyurethane synthesis proceeds via a step‐growth mechanism, the attainable molecular weight is highly sensitive to stoichiometric imbalance, trace water, and small amounts of monofunctional impurities. The observed lower molecular weights of the copolymerization may originate from a combination of several factors, such as residual moisture associated with hygroscopic **TEG**, differences in the reactivity of monomer **Q**, **TEG**, and **BHF** toward isocyanate groups, and possible small deviations from ideal bifunctionality/stoichiometry. The slightly lower experimental incorporation of the crosslinkable unit **BHF** compared with the feed composition also suggests that it may participate less efficiently than the other diols under the present conditions. Further optimization of monomer purification, drying, and feed stoichiometry will be required to maximize molecular weight.

**TABLE 1:** Molecular structures of the obtained polymers. The molar ratios of monomer **Q**, **TEG**, and **BHF** were expressed as n_Q_, n_T_, n_F_ and n_NCO_, respectively. The columns “Feed” and “Exp.” refer to the ratios for the monomer mixtures and the polymer composition (according to ^1^H NMR spectra), respectively. Number‐average molecular weight (M_n_) and polydispersity index (*Ð*) were measured by gel permeation chromatography (GPC) in tetrahydrofuran.

Polymer	n_Q_ ^a^		n_TEG_ ^a^		n_BHF_ ^a^		M_n_ (kDa)	*Ð*
	Feed	Exp.	Feed	Exp.	Feed	Exp.		
**P(Q‐M)**	—	—	—	—	—	—	280	1.3
**P(Q_35_T_41_F_19_‐M)**	0.33	0.35	0.41	0.47	0.27	0.19	10	1.6
**P(Q_21_T_58_F_21_‐M)**	0.20	0.21	0.56	0.58	0.24	0.21	20	2.1
**P(Q‐H)**	—	—	—	—	—	—	41	2.1
**P(Q_35_T_49_F_16_‐H)**	0.33	0.35	0.42	0.49	0.25	0.16	18	2.0
**P(Q_23_T_62_F_15_‐H)**	0.20	0.23	0.56	0.62	0.24	0.15	35	1.6

^a^ Determined from ^1^H‐NMR spectra (Figures S11–S16) and Equations S2–S7.

The molecular structure of the obtained polymers was investigated by ^1^H‐NMR, ^13^C‐NMR, and FTIR spectra (Figures S11–22). The ^1^H‐NMR spectra of **P(Q‐M)**, **P(Q_35_T_41_F_19_‐M)**, and **P(Q_21_T_58_F_21_‐M)** are shown in Figure 2, while the spectra for other obtained polymers are shown in Figures S14–S16. For **P(Q‐M)**, the urethane proton (*a*) was observed at∼ 9.7 ppm, along with two other peaks at 7.5 and 7.1 ppm, derived from the **MDI** monomer (protons *b* and *c*, respectively). Additional peaks at 6.4–7.0, 4.5, 4.1, and 3.7 ppm originated from the monomer **Q** moiety (protons *g‐i*, *e*, *f*, and *j*, respectively). The copolymers with **BHF** and **TEG**, **P(Q_35_T_41_F_19_‐M)** and **P(Q_21_T_58_F_21_‐M)**, exhibited spectra similar to **P(Q‐M)**, with additional proton peaks of **BHF** (*n* and *o*, at 6.4 and 5.1 ppm, respectively) and the **TEG** segment (protons *k*, *l*, and *m*, at 4.2, 3.6, and 3.5 ppm, respectively). The ^1^H‐NMR spectra of the **H_12_MDI**‐based PUs displayed characteristic peaks for the urethane proton (7.3 ppm) and the aliphatic peaks originating from the **H_12_MDI** segment (0.7–2.0 ppm), as well as the residue structures for monomer **Q**, **BHF** and **TEG** (protons *g‐i*, at 7.0–6.4 ppm, protons *n* and *o* at 6.4–4.9 ppm, and protons *k‐m* at 4.2–3.5 ppm, respectively). ^13^C‐NMR spectra of all PUs showed a peak at 153–155 ppm corresponding to the urethane linkage, which was further corroborated by the band at 1700–1730 cm^−1^ in the respective FTIR spectra.

**FIGURE 2:**
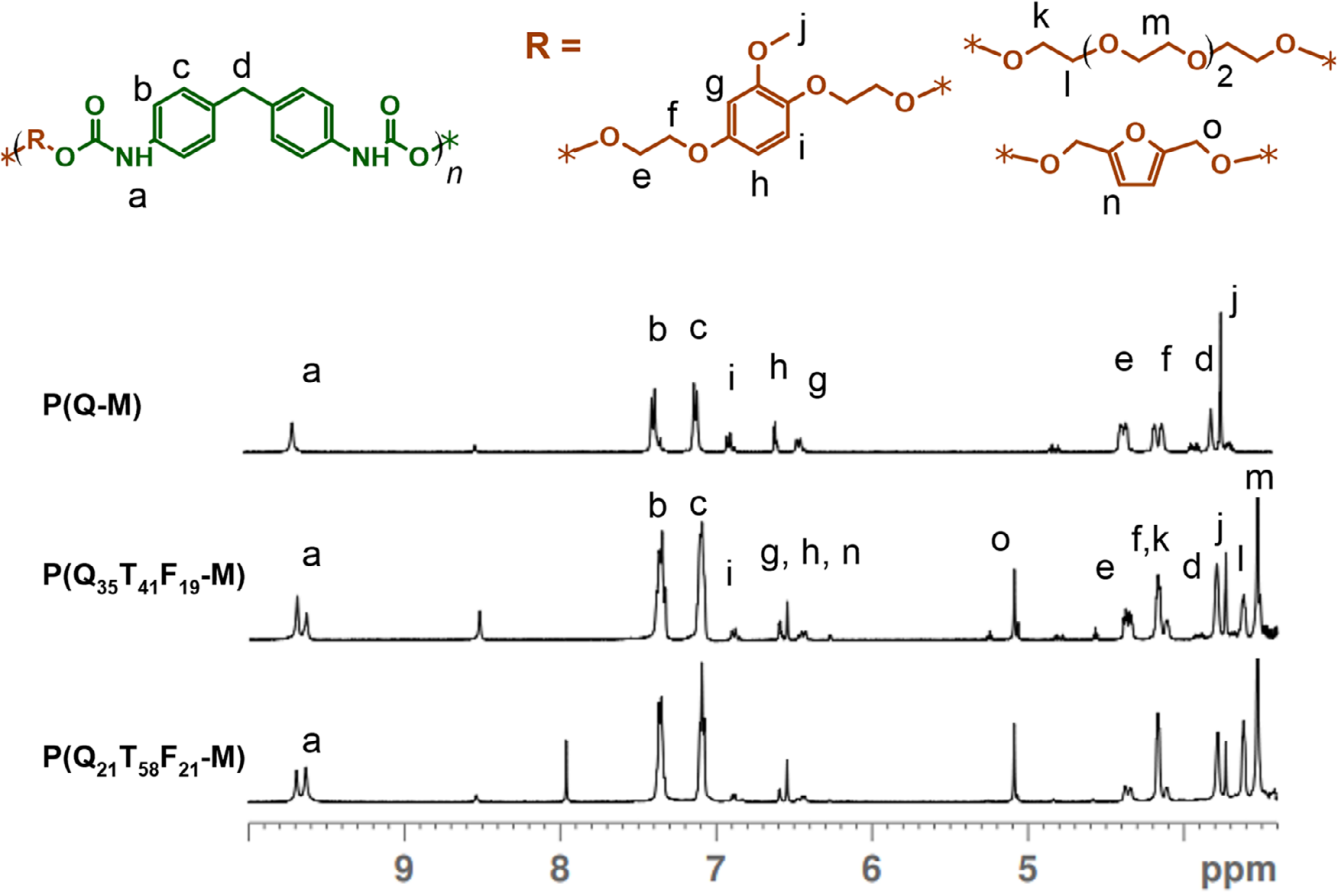
^1^H‐NMR spectra of **P(Q‐M)**, **P(Q_35_T_41_F_19_‐M)**, and **P(Q_21_T_58_F_21_‐M)** in DMSO‐d_6_.

Herein, the sustainability of our polyurethanes was evaluated in terms of (potentially) biobased content. Considering that all the diols can be obtained from biobased feedstock, including tetraethylene glycol, potentially obtained from biobased ethylene glycol [47, 48, 49], the biobased content was approximately 41% for **P(Q_35_T_41_F_19_‐M)** and **P(Q_35_T_49_F_16_‐H),** and 40% for **P(Q_21_T_58_F_21_‐M)** and **P(Q_23_T_62_F_15_‐H)**. In comparison, the biobased content of previously reported reversible crosslinked fibers based on 5‐[[4‐(hydroxymethyl)‐2‐methoxyphenoxy]methyl]‐2‐furanmethanol was approximately 35% [18], and the value for the fiber based on furfuryl methacrylate‐based fibers ranged from 10% to 20% [62, 63]. These results demonstrate that hydroxyethylation of the lignin‐derived aromatic precursor is an effective strategy to generate a reactive diol building block for polyurethane synthesis, enabling incorporation of rigid aromatic content while maintaining polymerization efficiency.

### 2.3 Thermal Analysis

Thermal stability of the obtained polymers was assessed by thermogravimetry (TGA, thermograms shown in Figures S29–34). The initial thermal decomposition temperature (i.e., temperature with 5% mass loss, T_5_) of the obtained PUs was in the range of 217°C–272°C (Table S1), which was generally consistent with other previously reported biobased PUs [18, 44, 60]. The obtained PUs based on MDI exhibited lower thermal stability compared to their analogs based on H_12_MDI (T_5_ value at least 17°C lower), which was also consistent with the literature [64, 65]. In addition, the incorporation of TEG and BHF crosslinkable units also lowered the thermal stability, and T_5_ was ∼36°C–38°C lower for **P(Q_21_T_58_F_21_‐M)** and **P(Q_23_T_62_F_15_‐H)** compared to their respective homopolymers without TEG or BHF units.

In the first derivative curves (DTG, Figures S29–S34), 1–3 maximal rates for thermal decomposition events were observed between 200°C and 400°C, followed by another single thermal decomposition rate maximum at 400°C–500°C. The first set of events (200°C–400°C) could be attributed to the thermal decomposition of BHF moieties (the monomer presented T_5_ at 140°C, Figure S35) and urethane linkages releasing various kinds of gases (e.g., CO, CO_2,_ and ethylene, etc.) as reported for many other PUs in the literature [64, 65, 66]. The first rate maximum was largely affected by the chemical structures, which was almost 100°C lower for the MDI‐based PUs compared to the corresponding polymer based on H_12_MDI. The second thermal decomposition event (400°C–500°C), attributed to the thermal degradation of the ether linkages in monomer **Q** and tetraethylene glycol structure [67], did not show significant difference for the PUs based on MDI or H_12_MDI. Finally, the char yield (CY) after TGA measurements at 600°C was generally higher for MDI‐based PUs (CY ∼11%–17%) compared to those of H_12_MDI‐based (CY < 2%), which was consistent with the existence of aromatic units in MDI‐based polymers [66].

The thermal properties of the obtained PUs were also investigated by differential scanning calorimetry (DSC). As shown in the second heating curves (Figure S36), all the obtained PUs were completely amorphous without any melting endotherm. The T_g_ was in the range of 31°C–124°C, depending on the chemical structures. In general, the PUs based on MDI showed higher T_g_ than those based on H_12_MDI, due to the more rigid structure of MDI, which has been reported before [66]. For example, T_g_ of **P(Q‐M)** and **P(Q‐H)** were 123°C and 90°C, respectively. In addition, as expected, the inclusion of a soft segment in the PU structure by copolymerization with TEG also led to lower T_g_, as shown by the difference between the T_g_ of **P(Q_35_T_49_F_16_‐H)** and **P(Q_23_T_62_F_15_‐H)** (81°C and 31°C, respectively) compared to that of the homopolymer **P(Q‐H)** (T_g,_ ∼ 90°C).

### 2.4 Fiber‐Spinning of P(Q_x_T_y_F_z_‐M) and P(Q_x_T_y_F_z_‐H)

Fiber spinning experiments were conducted for **P(Q_21_T_58_F_21_‐M)**, **P(Q_35_T_41_F_19_‐M)**, **P(Q_23_T_62_F_15_‐H)**, and **P(Q_35_T_49_F_16_‐H)**, according to a similar wet‐spinning process in the literature [18, 68, 69]. Polymer solutions (40 wt% in DMF) were extruded through a 12‐hole spinneret (hole diameter = 80 µm) into a coagulation bath with deionized water (Scheme 1C), providing a preliminary demonstration of wet‐spinning processability for selected polymer compositions. Among the investigated compositions, **P(Q_21_T_58_F_21_‐M)** and **P(Q_35_T_49_F_16_‐H)** showed suitable preliminary spinnability, yielding multi‐filament, noncrosslinked fibers collected at pick‐up speeds of 5, 6, and 7 m min^−^^1^ (corresponding to draw ratios of 1.0, 1.2, and 1.4, respectively). At higher pick‐up speeds, the fibers lost integrity and broke during processing. In contrast, **P(Q_35_T_41_F_19_‐M)** and **P(Q_23_T_62_F_15_‐H)** failed in wet spinning. The former coagulated too quickly, clogging the spinneret holes, while the latter coagulated too slowly, preventing fiber formation and resulting in mechanically fragile filaments. Furthermore, the two polymers that were successful for fiber spinning, i.e., **P(Q_21_T_58_F_21_‐M)** and **P(Q_35_T_49_F_16_‐H)**, were also prepared into crosslinked fibers by spinning the polymer solution containing bismaleimide. The crosslinking kinetics depend on several factors, including the polymer structure and the processing temperature. Based on literature reports, these conditions were selected to promote extensive Diels–Alder crosslinking of the furan‐containing fibers [41, 70]. To confirm the crosslinking efficiency, the resulting fibers were submerged in DMSO‐d_6_ solvent (a good solvent for the noncrosslinked polymers and the monomers) at 25°C for 72 h. Afterwards, the resulting mixture was subjected to ^1^H NMR spectroscopic analysis, which showed no signals corresponding to the noncrosslinked furan units at 6.4 ppm (Figure S37). This result indicates that no detectable extractable noncrosslinked furan‐containing species remained under the experimental conditions, supporting efficient crosslinking of the fibers. The fibers were also analyzed by FTIR before and after crosslinking, which revealed no noticeable changes (Figure S37C). In principle, the progress of the Diels–Alder (or retro‐Diels–Alder) reaction could be assessed by monitoring the increase or decrease of the peaks at 1013, 1345, and 1436 cm^−^^1^, corresponding to C─O─C stretching, furan breathing, and furan/pyrrole stretching vibrations, respectively [41]. However, monomer **Q** also exhibited absorption bands at these wavenumbers, and combined with the relatively low concentration of BHF/bismaleimide units in the polymer structures, this prevented the extraction of meaningful quantitative information about the crosslinking process. Likewise, no meaningful quantitative information regarding the crosslinking process could be obtained by ^1^H NMR spectroscopy.

The obtained noncrosslinked and crosslinked fibers of **P(Q_21_T_58_F_21_‐M)** and **P(Q_35_T_49_F_16_‐H)** were characterized by ^1^H NMR spectroscopy (note, the crosslinked polymers were insoluble at room temperature but became soluble after heating at 140°C in DMSO‐d_6_). As a result, the NMR peaks of all the obtained fibers showed no significant difference compared to that of the initial polymer powder (Figures S39–S42), indicating the possibility of re‐using these polymers for fiber spinning. Furthermore, the thermal properties of the obtained fibers were measured by TGA and DSC (Table S2). The thermal stability of the obtained fibers remained relatively unchanged after spinning (Figures S43–S45). Both noncrosslinked/crosslinked **P(Q_21_T_58_F_21_‐M)** and **P(Q_35_T_49_F_16_‐H)** fibers showed a ± 10°C variation in T_5_ compared to the respective original polymer (Table S2). The T_g_ of noncrosslinked **P(Q_21_T_58_F_21_‐M)** fibers (77°C–81°C) was higher compared to that of the original polymer powder (T_g_ ∼70°C), while the T_g_ of the noncrosslinked **P(Q_35_T_49_F_16_‐H)** fibers (T_g_ ∼ 75°C–80°C) remained similar to that of the original polymer (T_g_ ∼78°C).

The T_g_ of the crosslinked fibers, in principle, is nontrivial to investigate because crosslinking and de‐crosslinking could occur during the first heating and cooling cycle [71]. The T_g_ of crosslinked **P(Q_21_T_58_F_21_‐M)**, measured by the “normal” DSC experiment, was 83°C and 86°C at the first and second heating, respectively. For **P(Q_35_T_49_F_16_‐H)**, the T_g_, also measured by the “normal” DSC experiment, was 83°C at the second heating (it could not be measured at the first heating due to the occurrence of other unknown events, possibly residual solvent evaporation). To further investigate the T_g_ of the crosslinked fibers, DSC experiments were repeated, and the fibers were submitted to an isotherm at 60°C for 10 h during the first cooling (Figure 3A), to mitigate the effects of de‐crosslinking caused by retro‐Diels‐Alder reaction. Under these conditions, T_g_ was observed at 87°C and 84°C (both taken at the second heating) for the crosslinked fibers of **P(Q_21_T_58_F_21_‐M)** and **P(Q_35_T_49_F_16_‐H)**, respectively. These values of T_g_ were significantly higher than the respective noncrosslinked fibers or polymer powder, presumably due to the restricted mobility of the polymer chains upon crosslinking. The observed enhanced T_g_ of these fibers upon crosslinking is consistent with other crosslinked materials [72]. In the DSC curves, one thermal event at 100°C–140°C was observed, which is consistent with the retro‐Diels‐Alder reaction [18, 71]. To further examine the thermal reversibility of the crosslinked networks, the fiber **P(Q_21_T_58_F_21_‐M)** (obtained at draw ratio 1.0) was heated at 140°C for 20 min under a nitrogen atmosphere, a condition sufficient to promote de‐crosslinking through the retro‐Diels–Alder reaction. Subsequent DSC analysis revealed a T_g_ of 82°C during the first heating, comparable to that of the noncrosslinked fiber (77°C, Table S2), confirming decrosslinking. Afterwards, the resulting de‐crosslinked fiber was annealed at 60°C for 10 h, which led to an increased T_g_ ∼ 87°C (similar to the original crosslinked fiber), indicating the reformation of the crosslinked structure. Repeating this de‐crosslinking and annealing cycle resulted in repeated T_g_ values for the de‐crosslinked and re‐crosslinked fibers during each cycle, demonstrating the reversible nature of the thermally induced crosslinking/de‐crosslinking process.

**FIGURE 3:**
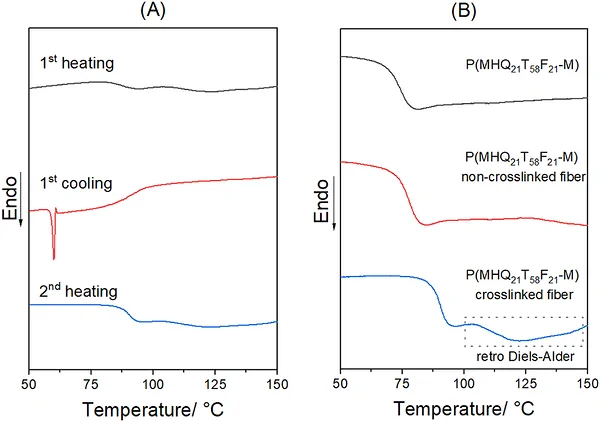
(A) DSC results for **P(Q_21_T_58_F_21_‐M)** crosslinked fiber and (B) DSC results (second heating curves only) for **P(Q_21_T_58_F_21_‐M)** (polymer powder), noncrosslinked fibers of **P(Q_21_T_58_F_21_‐M)**, and the crosslinked fiber of **P(Q_21_T_58_F_21_‐M)**. All fibers were spun at a draw ratio = 1.0.

The mechanical properties of the obtained noncrosslinked and crosslinked fibers were investigated by stress‐strain tests (Figures 4, S46, and S47; Table S2). Qualitatively, most of the measured elongation at break, tenacity, and Young's Modulus for noncrosslinked fibers generally decreased with the increase of draw ratio, which could be attributed to the effects of over‐orientation [73]. The only exceptions were the Young's modulus of the noncrosslinked **P(Q_21_T_58_F_21_‐M)** fibers and the tenacity of the noncrosslinked **P(Q_35_T_49_F_16_‐H)** fibers. The corresponding crosslinked fiber, however, showed a different dependence of the mechanical properties with the draw ratio. For the cross‐linked **P(Q_35_T_49_F_16_‐H)** fibers, tenacity and Young's modulus increased as the draw ratio also increased from 1.0 to 1.4, while elongation at break decreased. The measured preliminary mechanical data for **P(Q_21_T_58_F_21_‐M)** showed less variation within the error margin. The relatively large error bars could be related to the known core‐skin effect, in which the fiber skin and core coagulate at different rates [74]. Since wet spinning is diffusion‐controlled, small fluctuations in coagulation bath composition can alter solvent–nonsolvent exchange kinetics, leading to variations in radial fiber morphology. It should be noted that these fibers were produced using a nonoptimized laboratory‐scale wet‐spinning setup. Consequently, the mechanical properties reported in this study do not represent the full performance potential of the materials. Further optimization of the spinning process and processing parameters may lead to improved mechanical performance.

**FIGURE 4:**
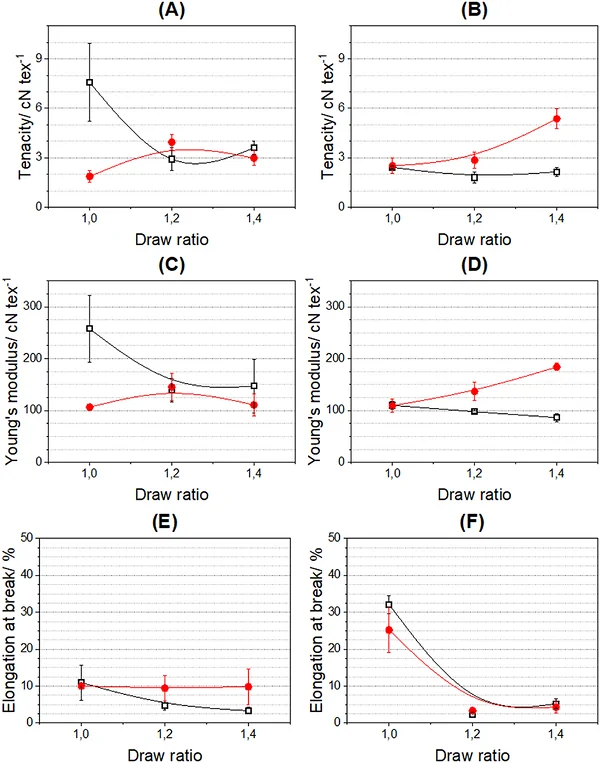
Tenacity of (A) **P(Q_21_T_58_F_21_‐M)** and (B) **P(Q_35_T_49_F_16_‐H)** fibers. Young's modulus of (C) **P(Q_21_T_58_F_21_‐M)** and (D) **P(Q_35_T_49_F_16_‐H)** fibers. Elongation at break of (E) **P(Q_21_T_58_F_21_‐M)** and (F) **P(Q_35_T_49_F_16_‐H)** fibers. Open squares and closed circles represent noncrosslinked and crosslinked fibers, respectively. Error bars correspond to the standard deviation.

The impact of crosslinking on mechanical properties was also revealed by comparing the crosslinked fibers with the corresponding noncrosslinked fibers under the same draw ratio. For **P(Q_35_T_49_F_16_‐H)** fibers obtained at higher draw ratios (1.2 and 1.4), the tenacity and Young's modulus, and elongation at break were increased up to 240% after crosslinking, exhibiting an improvement of mechanical performance similar to that of other Diels‐Alder‐crosslinked polyurethanes reported recently [75]. The elongation at break remained mostly similar. For **P(Q_21_T_58_F_21_‐M)**, the fibers obtained at a lower draw ratio (1.0) showed decreased tenacity and Young's modulus after crosslinking, while the fibers obtained at draw ratios of 1.2 and 1.4 showed similar Young's modulus and slightly decreased tenacity after crosslinking. Considering the error, the elongation at break of **P(Q_21_T_58_F_21_‐M)** obtained at higher draw ratios (1.2 and 1.4) fibers slightly decreased after crosslinking. Interestingly, the mechanical properties of H_12_MDI‐based fibers were (generally) improved after crosslinking, while the opposite was observed for MDI‐based fibers. Although the underlying reasons behind this difference are not yet fully understood, FTIR spectra of the MDI‐ and H_12_MDI‐based fibers before and after crosslinking (Figure S38) indicate that the effect of crosslinking on urethane carbonyl hydrogen bonding differs considerably between the two systems. In the MDI‐based system, crosslinking led to an increase in hydrogen‐bonded carbonyl (observed at 1703 cm^−1^) contributions accompanied by a decrease in free carbonyl content (observed at 1724 cm^−1^). In contrast, the H_12_MDI‐based system exhibited the opposite trend, with a decrease in hydrogen‐bonded species and a corresponding increase in free carbonyl content after crosslinking (both observed at 1692 and 1708 cm^−1^, respectively). A possible hypothesis to explain the different effect of crosslinking on the mechanical behavior of **MDI** and **H_12_MDI**‐based is that the mechanical performance of the MDI‐based fibers could be governed not only by the covalent crosslinking but also the rigid aromatic hard segments. In this MDI‐based system, the bulky Diels–Alder adducts may disrupt the packing of the hard aromatic domains and alter the distribution of hydrogen‐bonded urethane groups, leading to less effective load transfer despite the formation of crosslinking. In contrast, the aliphatic H_12_MDI‐based polymer could be less dependent on the ordered packing of aromatic segments, and thus the formation of covalent Diels–Alder network may become the dominant factor. This hypothesis is consistent with the different FTIR carbonyl‐band changes observed for the two systems, although further studies would be required to confirm the phase‐separation behavior.

Overall, these results indicate that the mechanical response of the fibers is governed by the combined effects of polymer composition, wet‐spinning conditions, draw ratio, and reversible crosslinking. However, it should be noted that the mechanical properties are still inferior to those of commercial (noncrosslinked) elastane fibers, and the present fiber data should be regarded as a proof‐of‐concept demonstration of processability and structure–property tunability, rather than an optimized fiber‐performance study. In the meantime, this work only includes a limited number of specimens, so the statistical significance of the observed mechanical properties remained to be further explored in the future.

### 2.5 Investigation of Crosslinking/Decrosslinking

The reversible Diels–Alder chemistry between furan units as dienes and bismaleimide as the dienophile was investigated for the obtained polymers, since this reaction has recently been explored as a promising strategy for reversibly crosslinked polyurethanes [75]. First, the films of the obtained polymers, **P(Q_21_T_58_F_21_‐M)**, **P(Q_35_T_41_F_19_‐M)**, **P(Q_23_T_62_F_15_‐H)**, and **P(Q_35_T_49_F_16_‐H)**, were prepared by casting the polymer solutions in DMF in the presence of bismaleimide (Scheme 2). Due to the presence of bis‐maleimide (crosslinker), the films underwent crosslinking through Diels‐Alder chemistry at 60°C under nitrogen. The chemical crosslinking was qualitatively confirmed by the observation that the films became swollen but completely insoluble in the respective solvent (for film casting) at room temperature (while the noncrosslinked films were soluble). De‐crosslinking of the crosslinked films could be achieved by heating the films to 140°C (at which the retro‐Diels‐Alder reaction occurred), leading to the complete solubilization and the formation of the original noncrosslinked polymers and bismaleimide (both were soluble in DMF). The resulting solution containing the noncrosslinked polymers and the crosslinker was cast into a film and re‐crosslinked at 60°C to yield recycled films, demonstrating qualitatively the reversible crosslinking.

Building on these results, the reversible crosslinking of the two polymers with promising spinnability (i.e., **P(Q_21_T_58_F_21_‐M)** and **P(Q_35_T_49_F_16_‐H)** was further investigated indirectly by different methods. The gel fraction of these two polymers was measured as 90% and 78%, respectively, which confirmed the effectiveness of the crosslinking under the experimental conditions. The swelling ratios of the films of **P(Q_21_T_58_F_21_‐M)** and **P(Q_35_T_49_F_16_‐H)** were also measured as (534 ± 343) % and (656 ± 447) %, respectively. The relatively large error range in the measured swelling ratios could be possibly attributed to the uneven solvent diffusion rate during film formation, which is known as the “skin effect” and can cause network inhomogeneities that could be related to the high error range of the measured swelling ratios [76, 77]. Further quantification of the crosslinking density based on swelling ratio is challenging, as it requires the Flory–Huggins interaction parameters that are currently unknown [78].

The expected changes in FTIR spectra after crosslinking could not be observed due to the low concentration of BHF/BHF Diels‐Alder adducts (ca. 8 mol% respective to the global composition) and overlapping with other peaks, as discussed earlier for the crosslinked fibers. Furthermore, **P(Q_21_T_58_F_21_‐M)** and **P(Q_35_T_49_F_16_‐H)**, were subjected to four more cycles (five in total) of de‐crosslinking and re‐crosslinking. The films formed after the last cycle were analyzed by GPC (Table 2 and Figure 5) and ^1^H NMR spectroscopy (Figures S48 and S49). The chromatogram of the recovered **P(Q_21_T_58_F_21_‐M)** after 5 cycles showed only a slight decrease in molecular weight, from 26.3 to 23.6 kDa, whereas **P(Q_35_T_49_F_16_‐H)** showed a slight increase from 18.4 to 19.4 kDa. In both cases, the ^1^H NMR spectra of the recovered polymers showed no significant differences compared to the respective original polymers. These results further verified the reversibility of the material within the limitations in the precision of the NMR and GPC methodologies, that is, the materials retain their composition while a slight variation of molecular weight was observed. However, the full characterization of reversibility, including a quantitative evaluation of the crosslinking density during these cycles, remains to be established (a further discussion is provided along with the DMA data, discussed at the end of this section).

**TABLE 2:** Number‐average molecular weight (M_n_) and polydispersity index (*Ð*) (measured by gel permeation chromatography in tetrahydrofuran) of **P(Q_21_T_58_F_21_‐M)** and **P(Q_35_T_49_F_16_‐H)** before and after five cycles of crosslinking/de‐crosslinking in different solvents.

Polymer	Solvent	M_n_ (kDa)	*Ð*
**P(Q_21_T_58_F_21_‐M)**	—^a^	26.3	1.8
	DMF	23.6	1.7
**P(Q_35_T_49_F_16_‐H)**	—^a^	18.4	2.0
	Anisole	16.4	1.9
	Cyrene	14.1	2.1
	DMF	19.4	1.8
	DMSO^b^	15.6	2.1
	GVL	5.1	2.4

^a^ Original polymer, before any crosslinking/de‐crosslinking cycles.

^b^ For the experiment carried out in the presence of cotton and PET, M_n_ and *Ð* were 18.9 kDa and 2.1, respectively.

**FIGURE 5:**
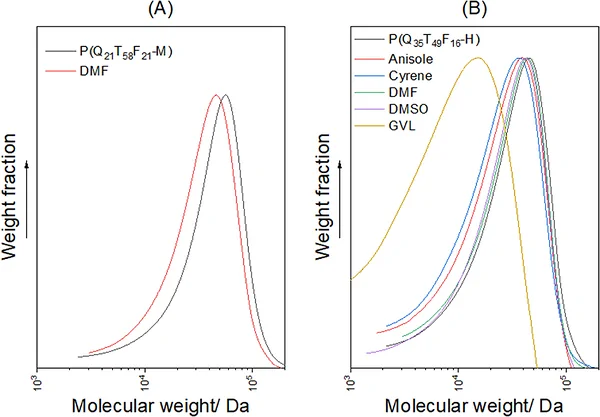
Gel permeation chromatogram (in tetrahydrofuran) of (A) **P(Q_21_T_58_F_21_‐M)** and **P(Q_21_T_58_F_21_‐M)** after 5 cycles of crosslinking/de‐crosslinking in DMF, and (B) **P(Q_35_T_49_F_16_‐H)** and **P(Q_35_T_49_F_16_‐H)** after five cycles of crosslinking/de‐crosslinking in different solvents.

To explore if the solvent used for de‐crosslinking/casting had any influence on the reversible crosslinking, the experiment was repeated for **P(Q_35_T_49_F_16_‐H)** using different solvents (DMSO, cyrene, anisole, and γ‐valerolactone (GVL), all considered of low environmental impact according to the Chem21 Solvent Selection Guide [46]). This is particularly important as processing of crosslinked polyurethanes is generally performed in high‐boiling‐point and toxic solvents, such as DMF and DMAc [27]. In all the studied solvents (DMSO, cyrene, anisole, and GVL), the polymer could be crosslinked at 60°C, which was confirmed by solubility tests, and de‐crosslinked at 140°C. After five cycles of crosslinking/de‐crosslinking, the polymers were analyzed by GPC and ^1^H‐NMR investigations. GPC chromatograms (Table 2 and Figure 5) revealed a slight decrease in molecular weight for the polymers recovered from the experiments performed with anisole, DMSO, and cyrene (respectively, 16.4, 15.6, and 14.1 kDa vs. 18.4 kDa for the original polymer). For the experiment with GVL, a pronounced decrease in M_n_ to 5.1 kDa and a significant increase in polydispersity (PDI = 2.5) were observed, suggesting substantial degradation. A possible explanation for the degradation in GVL is that the solvent can undergo ring‐opening to 4‐hydroxyvaleric acid under conditions involving moisture [79]. The resulting hydroxy‐acid species may participate in acid‐catalyzed urethane cleavage and exchange reactions [80].

The ^1^H NMR spectra of the recovered polymers showed no significant differences in terms of polymer composition, as the peaks corresponding to the polymer protons remained largely unchanged. However, a series of unidentified peaks, depending on the solvent used, appeared at different chemical shifts, most likely related to solvent degradation during the experiment. In principle, the **P(Q_35_T_49_F_16_‐H)** and **P(Q_21_T_58_F_21_‐M)** polymers obtained after five crosslinking/decrosslinking cycles in DMF are expected to exhibit somewhat similar spinnability as compared to the original polymer, while the crosslinking/decrosslinking cycles using the other solvents may interfere with the spinnability.

The reversible crosslinking of BHF units was also successfully repeated in the presence of both cotton and poly(ethylene terephthalate) (PET) substrates, providing a simplified model environment relevant to mixed textile waste streams. The experiments were conducted with **P(Q_35_T_49_F_16_‐H)** using DMSO as solvent. Notably, after five complete crosslinking/de‐crosslinking cycles, no detectable change in the chemical composition or molecular weight distribution species was observed, indicating good chemical stability of the reversible network under the tested model conditions. Interestingly, when cotton and PET were included, no sub‐products from solvent degradation were detected in the NMR spectrum, further supporting the robustness of the system in complex material environments.

Finally, to further assess the impact of reversible crosslinking on the mechanical performance of the polymers, dynamic mechanical analysis (DMA) measurements were conducted with crosslinked **P(Q_21_T_58_F_21_‐M)** and **P(Q_35_T_49_F_16_‐H)** samples, which were subjected to three consecutive crosslinking/de‐crosslinking cycles. As a result, the crosslinked polymers exhibited a comparable storage modulus in the glassy plateau region after each cycle (Figure S50), as the average variation in storage modulus over the three cycles was within approximately ± 10%. Therefore, no considerable loss of stiffness was observed upon repeated cycling, indicating that the reversible Diels–Alder crosslinking did not compromise the intrinsic mechanical integrity of the materials. Similarly, the tan δ profiles also remained consistent over the three cycles (Figure S51), indicating stable viscoelastic behavior throughout the reversible crosslinking/decrosslinking process. Unfortunately, crosslinking density and reversibility could not be quantitatively evaluated since the rubbery plateau region was not accessible due to the proximity of the glass transition (≈70°C–80°C) to the onset of de‐crosslinking (≈100°C). Instead, E′ decreases continuously with increasing temperature until the onset of viscous flow, without reaching a temperature‐independent plateau. This behavior is frequently observed in networks where a fully developed rubbery regime is not established within the accessible temperature window, which can be caused by (and may indicate) a relatively low crosslinking density.

## 3 Conclusion

This study presented a new molecular design to realize the reversible crosslinking concept toward the development of reprocessable thermosetting textile fibers. A new lignin‐derived rigid monomer Q and a sugar‐based diol with furan unit were exploited in the molecular design. The obtained polyurethanes exhibited tunable molecular weights (10–280 kDa) and glass transition temperatures (31°C–124°C). A thermally controlled reversible crosslinking and decrosslinking process enabled by the incorporated furan units could be conveniently performed up to five cycles without significant alteration in the molecular and thermal characteristics. Wet‐spinning was investigated for the obtained polymers to demonstrate the preliminary spinnability, and the obtained fibers showed distinct mechanical responses depending on composition, draw ratio, and crosslinking. These results indicate that the reversible Diels–Alder chemistry can be integrated into fiber‐forming polyurethanes and may provide a useful molecular design principle for future recyclable crosslinked textile materials. In the future, methodology development in fine‐controlling and characterizing the crosslinking ratio and rate, including a variable‐temperature FTIR measurement, and further optimization on the molecular design and synthesis parameters are expected to provide deeper insight and knowledge about recyclable textile fibers based on the reversible crosslinking strategy. Thorough optimization and environmental assessment (e.g., life cycle assessment, LCA) of the synthesis and processing will also be valuable in the future when upscaling processes are investigated.

## Conflicts of Interest

All authors declare no conflicts of interest.

## Supporting information




**Supporting File 1**: chem71541‐sup‐0001‐SuppMat.pdf.

## Data Availability

The data that support the findings of this study are available in the Supporting Information of this article.
