# Supplementary material for: Partially Biobased Polyurethanes With Reversibly Crosslinkable Furan Units Toward Recyclable Thermosetting Textile Fibres
Source: Chemistry. 2026 Aug 10;32(41):e71541. doi: 10.1002/chem.71541 (PMC13729944; doi:10.1002/chem.71541)
Supplement: Supplementary file 1 [file CHEM-32-e71541-s001.pdf]

*Supporting Information*

# Partially Biobased Polyurethanes with Reversibly Crosslinkable Furan Units toward Recyclable Thermosetting Textile Fibres

Rafael N. L. Menezes,[a] Fredrik Bäcklund,[b] Tam T. Nguyen, [a] Maria Maukonen, [b] Zengwei Guo, [b] Olga Gordivska, [a, c] Cecilia Hansson,[a,d] Baozhong Zhang*[a].

[a] Dr. R. N. L. Menezes, T. T. Nguyen, Dr. B. Zhang
Centre for Analysis and Synthesis, Department of Chemistry
Lund University P.O. Box 124, SE-22100 Lund, Sweden
E-mail: baozhong.zhang@chem.lu.se

[b] Dr. F. Bäcklund, M. Maukonen, Dr. Z. Guo
Division Materials and Production, Department of Polymers Fibers and Composites
RISE Research Institutes of Sweden, SE-43153, Mölndal, Sweden.

[c] Dr. O. Gordivska
Max IV, SE-224 84 Lund, Sweden.

[d] Dr. C. Hansson
Bona AB, SE-200 21 Malmö, Sweden.

* Corresponding author, email: baozhong.zhang@chem.lu.se


| Item | | Page |
|---|---|---|
| 1 | Materials and methods | S1 |
| 2 | Characterization of monomer **Q** | S9 |
| 3 | Characterization of PU – $^{1}$H-NMR spectra | S16 |
| 4 | Characterization of PU – $^{13}$C-NMR and FTIR spectra | S18 |
| 5 | Characterization of PU – GPC chromatograms | S26 |
| 6 | Thermal characterization – DSC and TGA | S29 |

| | | |
|---|---|---|
| 7 | Properties of PU fibers | S34 |
| 8 | Crosslinking/de-crosslinking investigation | S44 |


## 1. Materials and methods

4,4′-Methylenebis(phenyl isocyanate) (MDI, 98%), cyrene, anisole, γ-valerolactone (GVL), deuterated chloroform ($CDCl_3$) and deuterated dimethyl sulfoxide (DMSO-$d_6$) were purchased from Sigma-Aldrich. Dibutyltin dilaurate (DBTDL, metatin katalysator 712 ES) was purchased from Dow Chemicals. Tetraethylene glycol (TEG, 99%), Methanol (MeOH), dimethyl formamide (DMF), dimethyl sulfoxide (DMSO) and dimethyl acetamide (DMAc) were purchased obtained from Fischer Scientific. 4,4'-methylenebis(cyclohexyl isocyanate) (H12MDI, >99.5%) was supplied by Evonik. All solvents, reagents and chemicals were used without further purification. 2,5-bis(hydroxymethyl)furan (BHF) was synthesized according to the literature [49].

Proton nuclear magnetic resonance spectroscopy ($^1$H-NMR) was performed on a Bruker DR X400 at 400.13 MHz using polymer solutions in DMSO-$d_6$ at 2-5 mg mL$^{-1}$. Carbon-13 nuclear magnetic resonance spectroscopy ($^{13}$C-NMR) was performed on a Bruker Avance III at 125.8 MHz using polymer solutions in DMSO-$d_6$ at 20-25 mg mL$^{-1}$ solutions. Chemical shifts were assigned based on the residual solvent hydrogen. Fourier-transform infrared (FTIR) spectra were obtained using a Bruker Alpha FT-IR spectrometer with attenuated total reflection (ATR) setup. Thermogravimetry was performed on a TGA Q500 system (TA Instruments) under nitrogen atmosphere; samples were heated from 30 °C to 600 °C at 10 °C min$^{-1}$ heating rate. $T_{d,5}$ was determined as the temperature at 5% weight loss, and the char yield was taken as the remaining weight (in percentage) at 600 °C. Differential scanning calorimetry (DSC) was performed using a

DSC Q2000 analyzer (TA Instruments). Samples previously dried for 24 h under vacuum and at 50 °C were subjected to the following analysis program: (i) equilibrium at 20 °C; (ii) heating to ($T_{d,5}$ – 20 °C); (iii) isotherm for 2 min; (iv) cooling to −50 °C; (v) isotherm for 2 min; and (vi) heating to ($T_{d,5}$ – 20 °C). The scans were carried out at a rate of 10 °C $min^{-1}$ under nitrogen atmosphere and the thermograms were normalized with respect to the sample mass. Unless specified, the glass transition temperature ($T_g$) was taken at the second heating scan. Tensile testing was performed on a Vibrodyn/Vibroscope machine from Lenzing Technique GmbH & Co KG, Austria. The testing speed was 20 mm $min^{-1}$ and the gauge length was 20 mm. Each sample was measured and analyzed multiple times (at least 3 times, and the exact number of times for each sample varies), and the error was obtained as the standard deviation. Dynamic Mechanical Analysis (DMA) measurements were performed using a TA Instruments Q800 analyzer from 25 to 100 °C at a heating rate of 3 °C $min^{-1}$ and a frequency of 1 Hz. A strain sweep was conducted for all samples to determine the Linear Viscoelastic Region (LVR). A strain of 0.05%, within the LVR, was selected and used for all analyses. DMA specimens were prepared by crosslinking 0.40 g of polymer in a 40% (m/m) DMF solution containing 0.50 eq of bismaleimide relative to the furan units at 60 °C for 18 h. The crosslinked material was then dried in a vacuum oven at 50 °C for 3 days before being hot-pressed into a rectangular mold (1.0 × 5.0 × 17.5 mm, t × w × l). Hot pressing was carried out at 110 °C, approximately 30 °C below the respective decrosslinking temperature. After each DMA measurement, the sample was returned to the mold and heated at 140 °C for 2 h to induce decrosslinking, followed by 60 °C for 18 h to allow re-crosslinking. This cycle was repeated three times. After each cycle, the crosslinked samples were subjected to DMA analysis.

### 1.2 Synthesis

### 1.2.1 Monomer Q

To a 50 mL round-bottom flask were added 2-methoxyhydroquinone (1.04 g, 6.80 mmol), ethylene carbonate (1.38 g, 15.7 mmol), potassium carbonate (0.370 g, 2.64 mmol), and 2.0 mL of solvent (DMF, DMSO, cyrene, or additional 2 g of ethylene carbonate). The reaction mixture was heated to 140 °C under $N_2$ and stirred for 2 h, followed by cooling down to room temperature, and quenching with 2 mL of aqueous NaOH solution (0.5 mol $L^{-1}$). The reaction mixture was maintained under magnetic stirring at room temperature overnight. Afterwards, the solvent was removed under reduced pressure and the remaining solid was dissolved in 10 mL of 0.1 mol $L^{-1}$ aqueous NaOH, followed by two extractions with 10 mL of $CHCl_3$. The organic phase was dried over $Na_2SO_4$ and the solvent was removed under reduced pressure to obtain monomer **Q** as a brown solid. Yield = 73% (reaction in DMF). Melting point (DSC) = 83 °C. $^1$H NMR (500.17 MHz, $CDCl_3$, ppm): 6.87 (d, 1H, Ar-H), 6.55 (d, 1H, Ar-H), 6.42 (dd, 1H, Ar-H), 4.06 (q, 4H, -C$H_2$-OH), 3.96 (t, 2H, Ar-O-$CH_2$-), 3.88 (t, 2H, Ar-O-$CH_2$-), 3.84 (s, 3H, -O-$CH_3$). $^{13}$C NMR (600.13 MHz, $CDCl_3$, ppm): 154.0, 150.59, 142.36, 115.76, 104.40, 100.95, 77.27, 77.02, 76.77, 72.56, 69.67, 61.62, 61.26, 55.73.

### 1.2.2 P(Q-M)

Monomer **Q** (1.96 g, 8.60 mmol) and MDI (2.25 g, 9.00 mmol) were added to 5 mL of DMAc. The reagents were stirred for 10 min until a slurry mixture was obtained. Afterwards, 100 µL of a 10 % v/v DBTDL solution in 2-butanone were added and the temperature was raised to 50 °C. After 20 min the viscosity increased significantly, and then an additional aliquot of DBTDL (100 µL, 10 %v/v) was added into the reaction mixture, followed by the addition of 6 mL of DMAc, which reduced viscosity of the reaction mixture and allowed magnetic stirring. The reaction was

maintained at 50 °C and monitored by measuring FTIR spectrum of a small sample taken out from the reaction medium every 30 min. After no isocyanate peak was observed in the FTIR spectrum of the reaction medium, the reaction was completed. Afterwards, the reaction mixture was cooled to room temperature and precipitated in 200 mL of MeOH. The precipitated solid was filtered and subsequently dried under vacuum at 50 °C until constant weight, yielding **P(Q-M)** as a yellow solid. $^1$H NMR (400.13 MHz, DMSO-$d_6$, ppm): 9.7 (br, 2H, NH-(C=O)-O-), 7.4 (d, 4H, -$CH_2$-Ar-*H*), 7.0 (d, 4H, -$CH_2$-Ar-*H*), 6.9-6.4 (m, 3H, Ar-H), 4.4 (m, 4H, -O-C$H_2$-$CH_2$-O-Ar), 4.1 (m, 4H, O-$CH_2$-C$H_2$-O-Ar), 3.8 (br, 2H, Ar-$CH_2$-Ar), 3.7 (br, 3H, O-$CH_3$); $^{13}$C NMR (100.61 MHz, DMSO-$d_6$, ppm): 154.15, 153.82, 153.80, 153.56, 153.24, 152.94, 150.51, 150.45, 142.84, 142.26, 142.02, 138.01, 137.36, 137.32, 136.04, 135.98, 135.25, 129.23, 118.80, 118.71, 115.26, 115.10, 114.86, 114.40, 104.48, 101.34, 71.30, 20.18. 68.02, 66.71, 63.33, 63.25, 60.80, 60.03, 55.85, 30.69, 21.74; FT-IR: 1700 cm$^{-1}$; $T_g$ = 124 °C (DSC); $M_n$ = 280 kDa, $Đ$ = 1.3.

### 1.2.3 P($Q_{35}T_{41}F_{19}$-M)

Monomer **Q** (1.20 g, 5.26 mmol), BHF (0.53 g, 4.14 mmol), TEG (1.25 g, 6.41 mmol) and MDI (4.16 g, 16.6 mmol) were added to 10 mL DMAc with stirring at room temperature, forming a slurry. Afterwards, a solution of DBTDL in DMAc (500 µL of a 10 % v/v) was added, followed by a second addition of the same volume after 10 minutes. The reaction was maintained at 50 °C until no isocyanate peak was observed in the FTIR spectrum of the reaction medium. The purification was done by precipitation in 200 mL of MeOH. The obtained pale-yellow solid was dried under vacuum at 50 °C until constant weight, yielding polyurethane **P($Q_{35}T_{41}F_{19}$-M)**. $^1$H NMR (400.13 MHz, DMSO-$d_6$, ppm): 9.8-9.6 (m, 1.5H, NH-(C=O)-O-); 8.5 (s, 0.5H, -NH-(C=O)-O-), 7.4 (m, 4H, -$CH_2$-Ar-*H*), 7.1 (m, 4H, -$CH_2$-Ar-*H*), 6.9 (m, 0.25H, Ar-H), 6.6 (m, 0.34H, Ar-

H), 6.5 (s, 0.31H, -C=C-H), 6.4 (m, 0.31H, Ar-H), 5.1 (m, 0.65H, $CH_2$-C=C-), 4.4 (m, 1.15H, -O-C*H*$_2$-$CH_2$-O-Ar), 4.1 (m, 2.24H, -O-$CH_2$-C*H*$_2$-O-Ar and -N-(C=O)-O-C*H*$_2$-$CH_2$-O-$CH_2$-), 3.8 (br, 2H, Ar-$CH_2$-Ar), 3.7 (s, 1H, O-$CH_3$), 3.6 (br, 1.27H, -N-(C=O)-O-$CH_2$-C*H*$_2$-O-$CH_2$-), 3.5 (m, 2.83H, -(C*H*$_2$-C*H*$_2$)$_2$-); $^{13}$C NMR (100.61 MHz, DMSO-d$_6$, ppm): 162.73, 153.87, 153.84, 153.81, 153.57, 153.43, 153.34, 153.34, 153.24, 152.95, 150.80, 150.52, 150.45, 149.24, 142.85, 142.27, 142.02, 138.02, 137.41, 137.36, 137.33, 137.19, 136.21, 136.11, 136.05, 135.98, 135.89, 135.36, 135.26, 129.24, 118.72, 115.10, 114.86, 112.07, 111.81, 108.16, 104.48, 101.35, 101.27, 72.70, 71.31, 70.21, 70.15, 70.10, 69.05, 67.94, 66.72, 63.80, 63.34, 63.27, 60.59, 60.09, 60.04, 58.21, 58.03, 56.03, 55.85, 36.16, 31.15, 21.75; FT-IR: 1700 cm$^{-1}$; $T_g$ = 70 °C (DSC); $M_n$ = 10 kDa, $Đ$ = 1.6.

**1.2.4 P(Q$_{21}$T$_{58}$F$_{21}$-M)**

Monomer **Q** (0.75 g, 3.29 mmol), BHF (0.50 g, 3.90 mmol), TEG (1.75 g, 9.02 mmol) and MDI (4.29 g, 16.3 mmol) were added to 10 mL DMAc with stirring at room temperature, forming a solution. Afterwards, a solution of DBTDL in DMAc (500 µL of a 10 % v/v) was added, followed by a second addition of the same volume after 15 minutes. The reaction was maintained at 50 °C until no isocyanate peak was observed in the FTIR spectrum of the reaction medium. The purification was done by precipitation in 200 mL of MeOH. The obtained pale-yellow solid was dried under vacuum at 50 °C until constant weight, yielding polyurethane **P(Q$_{21}$T$_{58}$F$_{21}$-M)**. $^1$H NMR (400.13 MHz, DMSO-d$_6$, ppm): 9.8-9.6 (m, 1.7H, -NH-(C=O)-O-); 7.4 (m, 4H, -$CH_2$-Ar-*H*), 7.1 (m, 4H, -$CH_2$-Ar-*H*), 6.9 (m, 0.19H, Ar-H), 6.6 (m, 0.19H, Ar-H), 6.5 (s, 0.39H, -C=C-H), 6.4 (m, 0.24H, Ar-H), 5.1 (m, 0.86H, $CH_2$-C=C-), 4.4 (m, 0.81H, -O-C*H*$_2$-$CH_2$-O-Ar), 4.1 (m, 2.84H, -O-$CH_2$-C*H*$_2$-O-Ar and -N-(C=O)-O-C*H*$_2$-$CH_2$-O-$CH_2$-), 3.8 (br, 2H, Ar-$CH_2$-Ar), 3.7 (s,

0.71H, O-$CH_3$), 3.6 (br, 2.13H, -N-(C=O)-O-$CH_2$-C$H_2$-O-$CH_2$-), 3.5 (m, 4.44H, -(C$H_2$-C$H_2$)$_2$-); $^{13}$C NMR (100.61 MHz, DMSO-d$_6$, ppm): 162.73, 153.82, 153.78, 153.57, 153.33, 152.93, 150.80, 150.51, 149.96, 145.64, 142.26, 138.01, 137.41, 137.35, 137.18, 136.10, 135.95, 135.87, 135.24, 131.83, 129.21, 113.20, 112.06, 104.74, 101.26, 92.31, 70.15, 70.10, 69.05, 67.93, 66.71, 65.99, 63.79, 63.33, 63.25, 60.58, 58.02, 56.00, 55.86, 36.16, 31.15, 21.76; FT-IR: 1700 cm$^{-1}$; $T_g$ = 70 °C (DSC); $M_n$ = 20 kDa, $Đ$ = 2.1.

### 1.2.5 P(Q-H)

Monomer **Q** (1.21 g, 5.31 mmol) and MDI (1.45 g, 5.53 mmol) were added to 5 mL of 2-butanone with stirring at room temperature, forming a slurry. Afterwards, a solution of DBTDL in 2-butanone (10 % v/v, 126 µL) was added and the temperature was raised to 50 °C. After 30 min, an additional aliquot of DBTDL solution (126 µL of 10 % v/v) was added, followed by 2.5 mL of 2-butanone, to prevent gelation. After 30 min, one last aliquot of 2.5 mL of 2-butanone was added. The reaction was maintained at 50 °C until no isocyanate peak was observed in the FTIR spectrum of the reaction medium. The purification was done by precipitation in 200 mL of MeOH. The obtained white solid was dried under vacuum at 50 °C until constant weight, yielding polyurethane **P(Q-H)**. $^1$H NMR (400.13 MHz, DMSO-d$_6$, ppm): 7.2 (d, 2H, NH-(C=O)-O-), 6.8 (d, 1H, Ar-H), 6.6 (s, 1H, Ar-H), 6.4 (d, 1H, Ar-H), 4.2 (m, 4H, -O-C$H_2$-$CH_2$-O-Ar), 4.0 (m, 4H, O-$CH_2$-C$H_2$-O-Ar), 3.7 (s, 3H, O-$CH_3$), 1.9-0.7 (m, 22H, -$C_6H_{10}$-$CH_2$-$C_6H_{10}$-); $^{13}$C NMR (100.61 MHz, DMSO-d$_6$, ppm): 155.98, 155.84, 155.66, 153.74, 150.42, 142.29, 114.88, 104.90, 104.38, 101.24, 68.65, 68.06, 62.76, 62.69, 59.67, 55.83, 50.70, 33.69, 32.88, 32.19, 32.14, 31.95, 31.75, 29.26, 27.96, 27.90, 21.75, 15.06; FT-IR: 1700 cm$^{-1}$; $T_g$ = 70 °C (DSC); $M_n$ = 41 kDa, $Đ$ = 2.1.

**1.2.6 P($Q_{35}T_{49}F_{16}$-H)**

Monomer **Q** (1.20 g, 5.26 mmol), BHF (0.50 g, 3.90 mmol), TEG (1.25 g, 6.41 mmol) and H12MDI (4.29 g, 1.63 mmol) were added to 10 mL DMAc with stirring at room temperature, forming a slurry. Afterwards, a solution of DBTDL in DMAc (500 µL of a 10 % v/v) was added, followed by a second addition of the same volume after 15 minutes. The purification was done by precipitation in 200 mL of MeOH. The obtained white solid was dried under vacuum at 50 °C until constant weight, yielding polyurethane **P($Q_{35}T_{49}F_{16}$-H)**. $^{1}$H NMR (400.13 MHz, DMSO-$d_6$, ppm): 7.3-7.0 (m, 1.64H, -NH-(C=O)-O-); 6.8 (d, 0.34H, Ar-H), 6.6 (s, 0.31H, -C=C-H), 6.5-6.2 (m, 0.72H, Ar-H), 4.9 (m, 0.77H, $CH_2$-C=C-), 4.2 (br, 1.28H, -O-C$H_2$-$CH_2$-O-Ar), 4.1-4.0 (m, 2.97H, -O-$CH_2$-C$H_2$-O-Ar and -N-(C=O)-O-C$H_2$-$CH_2$-O-$CH_2$-), 3.7 (s, 0.97H, O-$CH_3$), 3.6-3.4 (m, 5.6H, -N-(C=O)-O-$CH_2$-C$H_2$-O-(C$H_2$-C$H_2$)$_2$-), 1.9-0.7 (m, 22H, -$C_6H_{10}$-$CH_2$-$C_6H_{10}$-); $^{13}$C NMR (100.61 MHz, DMSO-$d_6$, ppm) 162.72, 156.28, 155.90, 155.73, 155.46, 155.38, 155.29, 155.21, 151.05, 150.44, 148.76, 149.74, 142.77, 142.29, 142.11, 115.00, 111.56, 111.47, 111.33, 111.25, 108.07, 108.05, 104.51, 104.43, 101.34, 101.23, 72.69, 70.20, 70.16, 70.14, 70.09, 69.27, 68.07, 66.90, 60.58, 60.07, 60.03, 57.70, 57.53, 56.01, 55.82, 50.24, 47.58, 44.24, 36.16, 33.69, 32.88, 32.19, 32.14, 31.96, 29.26, 27.95, 27.90, 21.74; FT-IR: 1686 cm$^{-1}$; $T_g$ = 78 °C (DSC); $M_n$ = 18 kDa, *Đ* = 2.1.

**1.2.7 P($Q_{23}T_{62}F_{15}$-H)**

Monomer **Q** (0.75 g, 3.29 mmol), BHF (0.50 g, 3.90 mmol), TEG (1.75 g, 9.02 mmol) and H12MDI (4.50 g, 16.3 mmol) were added to 10 mL DMAc were added to 10 mL DMAc with stirring at room temperature, forming a solution. Afterwards, a solution of DBTDL in DMAc (500 µL of a 10 % v/v) was added, followed by a second addition of the same volume after 15 minutes.

The reaction was maintained at 50 °C until no isocyanate peak was observed in the FTIR spectrum of the reaction medium. The purification was done by precipitation in 200 mL of MeOH. The obtained white solid was dried under vacuum at 50 °C until constant weight, yielding polyurethane **P($Q_{23}T_{62}F_{15}$-H)**. $^{1}$H NMR (400.13 MHz, DMSO-$d_6$, ppm): 7.3-7.0 (m, 1.7H, -NH-(C=O)-O-); 6.8 (d, 0.27H, Ar-H), 6.6 (s, 0.26H, -C=C-H), 6.5-6.2 (m, 0.31H, Ar-H), 4.9 (m, 0.93H, $CH_2$-C=C-), 4.2 (m, 1.28H, -O-C$H_2$-$CH_2$-O-Ar), 4.1-4.0 (m, 2.84H, -O-$CH_2$-C$H_2$-O-Ar and -N-(C=O)-O-C$H_2$-$CH_2$-O-$CH_2$-), 3.7 (s, 1.18H, O-$CH_3$), 3.6-3.4 (m, 9.57H, -N-(C=O)-O-$CH_2$-C$H_2$-O-(C$H_2$-C$H_2$)$_2$-), 1.9-0.7 (m, 22H, -$C_6H_{10}$-$CH_2$-$C_6H_{10}$-); $^{13}$C NMR (100.61 MHz, DMSO-$d_6$, ppm) 162.72, 155.80, 153.85, 153.77, 153.56, 153.32, 152.93, 150.79, 150.50, 149.96, 145.64, 142.26, 138.01, 137.41, 137.35, 137.18, 136.09, 135.94, 135.86, 135.24, 131.82, 129.27, 129.23, 129.20, 121.07, 118.73, 118.31, 117.37, 113.21, 112.06, 104.48, 70.14, 70.09, 69.04, 67.92, 66.70, 65.99, 63.79, 63.62, 63.25, 58.02, 56.00, 55.85; FT-IR: 1690 $cm^{-1}$; $T_g$ = 31 °C (DSC); $M_n$ = 35 kDa, $Đ$ = 1.6.

### 1.3 Fibre-spinning

Solutions for non-crosslinked fibres were prepared by dissolving **P($Q_{21}T_{58}F_{21}$-M)** and **P($Q_{35}T_{49}F_{16}$-H)** in DMF to obtain 38 wt% and 40 wt% solutions, respectively. Solutions for producing crosslinked fibres were prepared similarly, but with addition of 0.5 equivalents of bismaleimide (with respect to the furan units) as the crosslinker. Afterwards, the spinning solution (loaded into a 10 mL syringe pump) was extruded at 0.3 mL $min^{-1}$ through a spinneret with 6 holes of 80 μm (diameter) into a 1 m coagulation bath filled with deionized water at room temperature. The fibre was collected by a take-up roller at take-up speeds of 5, 6, and 7 m $min^{-1}$ (draw ratio of 1.0, 1.2 and 1.4, respectively). The collected fibres were dried in an oven at 60 °C for 18 h.

## 2. Characterization of monomer Q

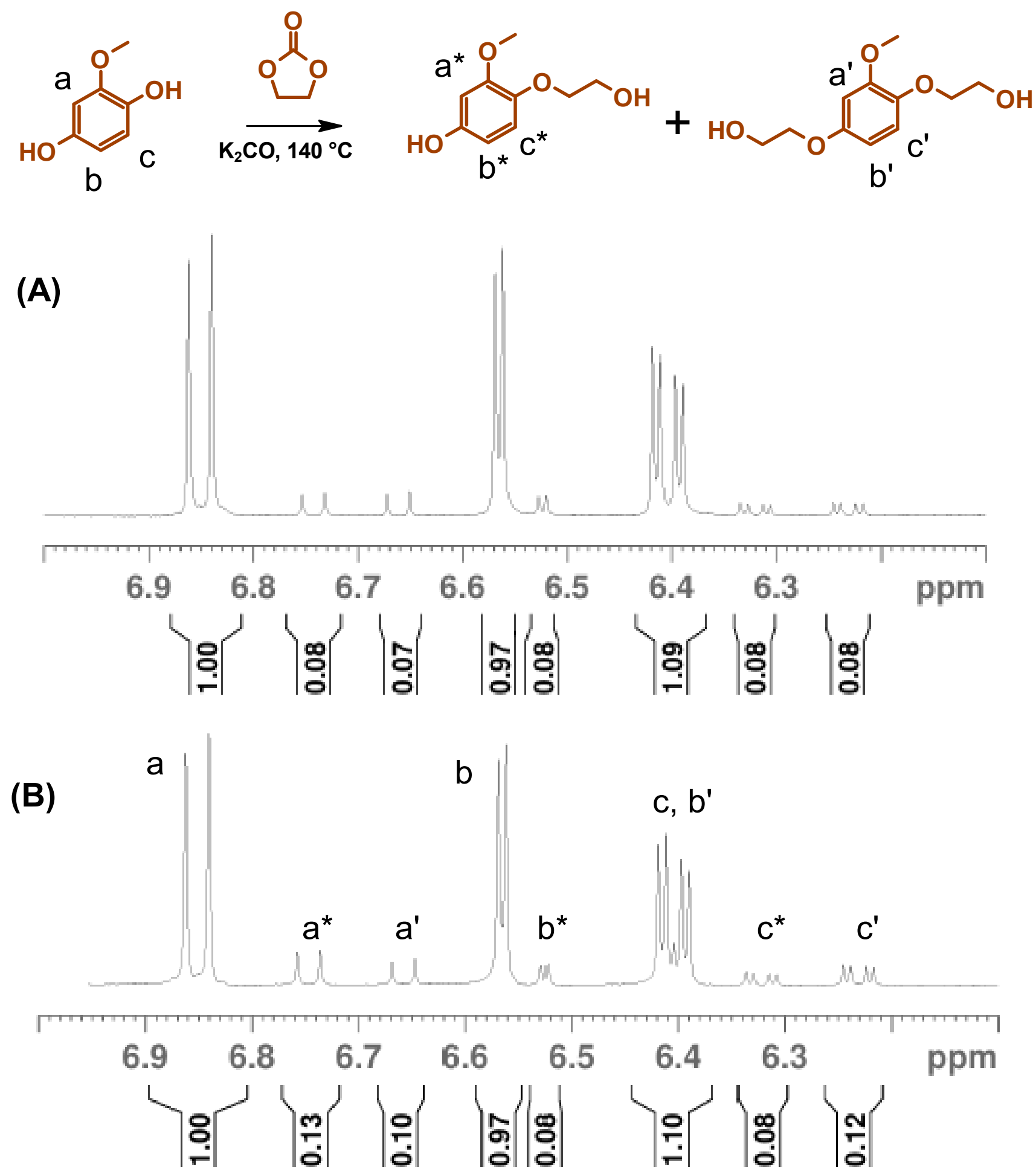


**Figure S1.** $^1$H-NMR spectra of reaction of crude product from ethoxylation of 2-methoxyhydroquinone in **(A)** DMF and **(B)** DMSO.

Conversion was calculated from Eq. S1:

$$c\ (\%) = \frac{H_a^*}{H_a + H_a' + H_a^*}\ 100\% \qquad \textbf{Equation S1}$$

Where $c$, $H_a$, $H_a'$ and $H_a^*$ represent, respectively, the conversion, and the area of corresponding to hydrogens a, a' and a* in Figure S1.

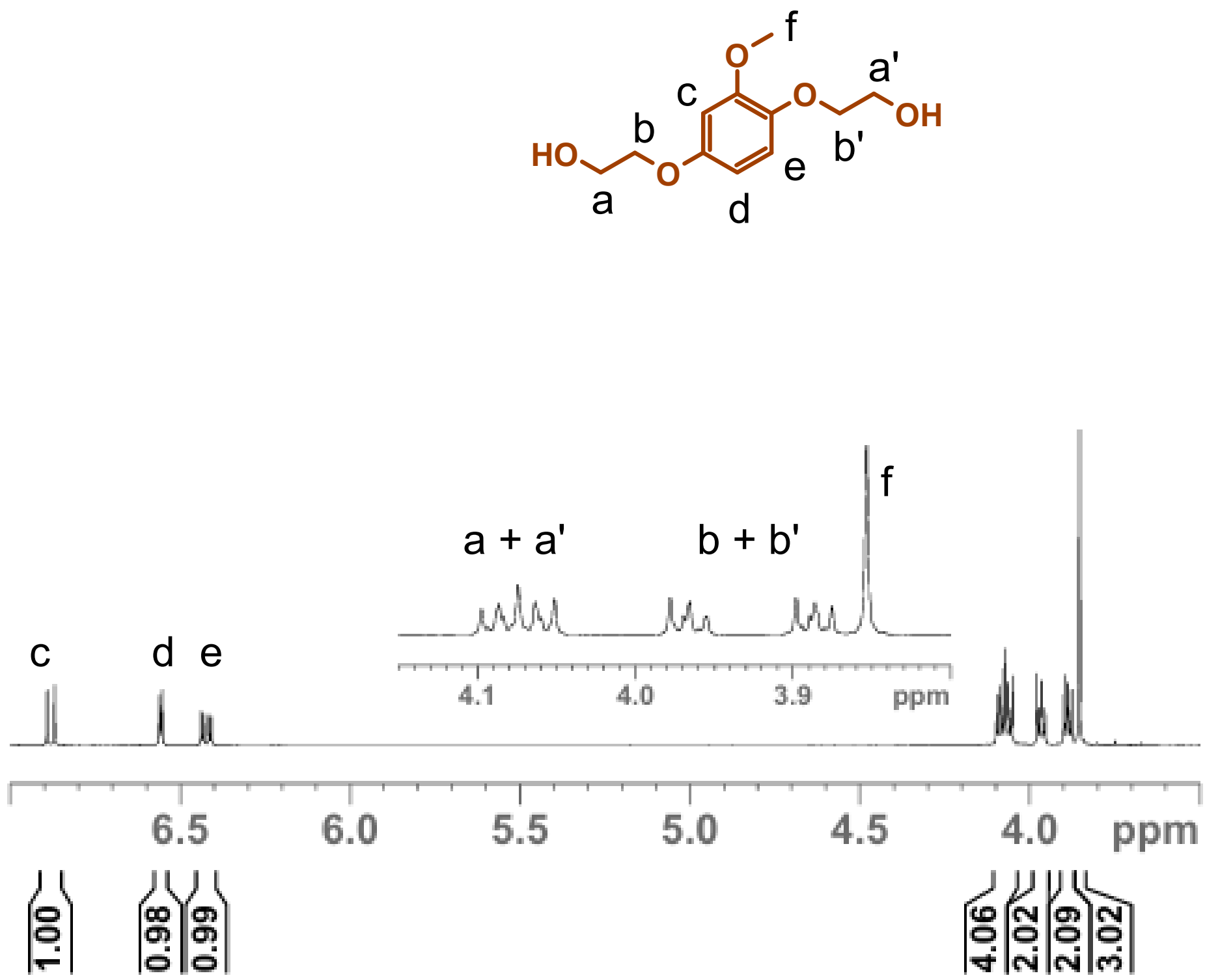


**Figure S2**. $^{1}$H-NMR spectrum of monomer **Q** (synthesized in DMF). Solvent: $CDCl_3$.

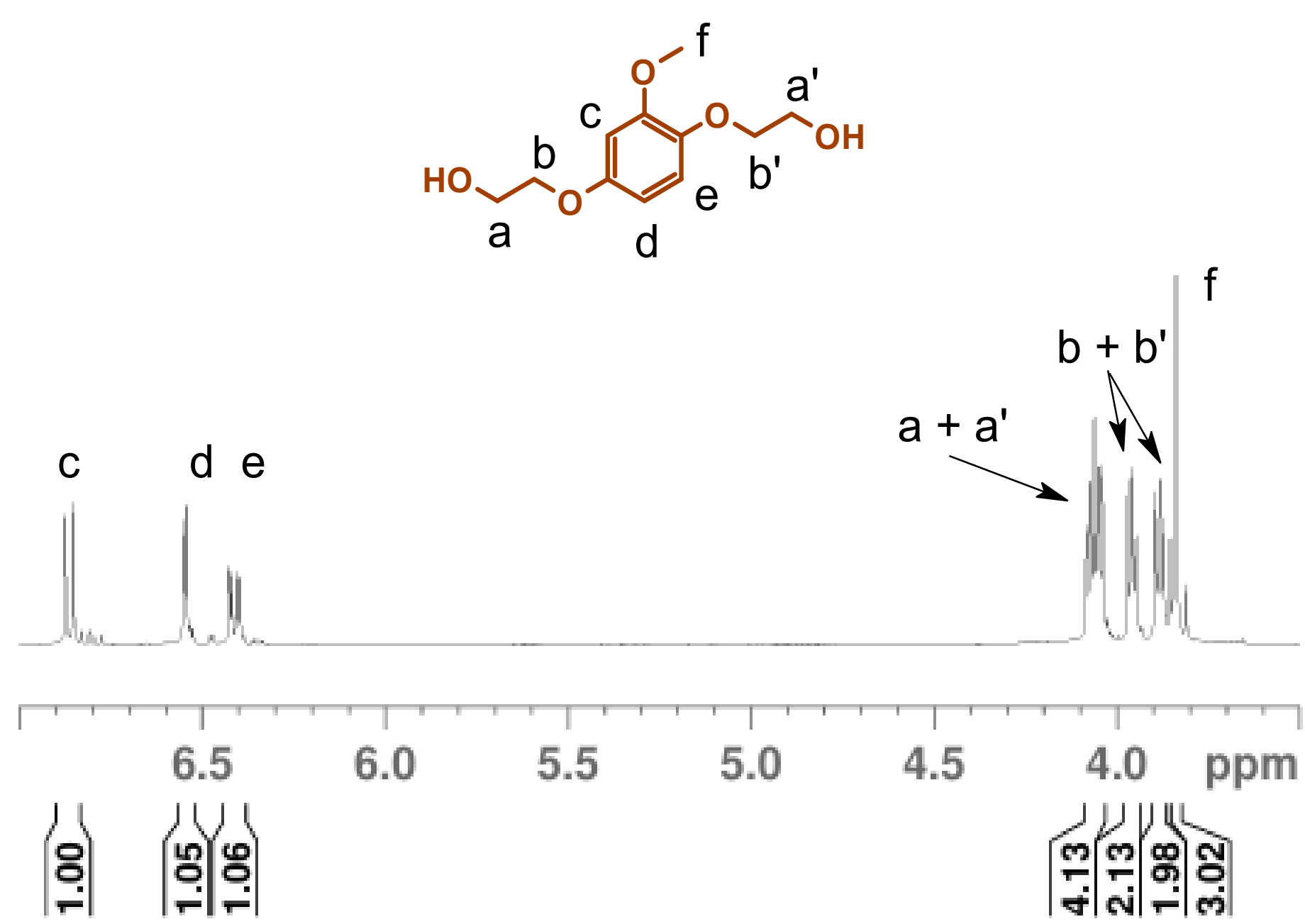


**Figure S3.** $^{1}$H-NMR spectrum of monomer **Q** (synthesized in DMSO). Solvent: $CDCl_3$.

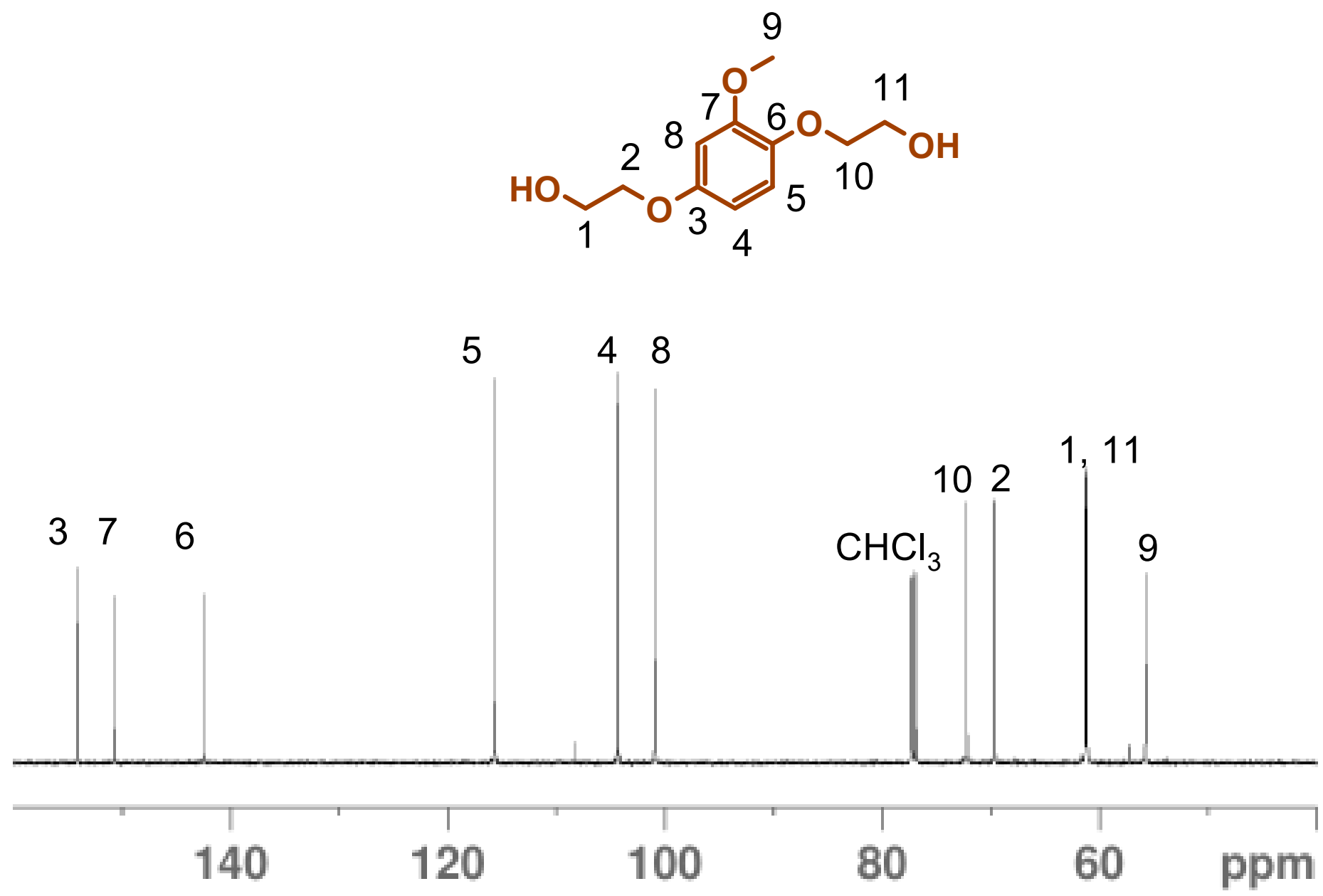


**Figure S4**. $^{13}$C-NMR spectrum of monomer **Q** in $CDCl_3$.

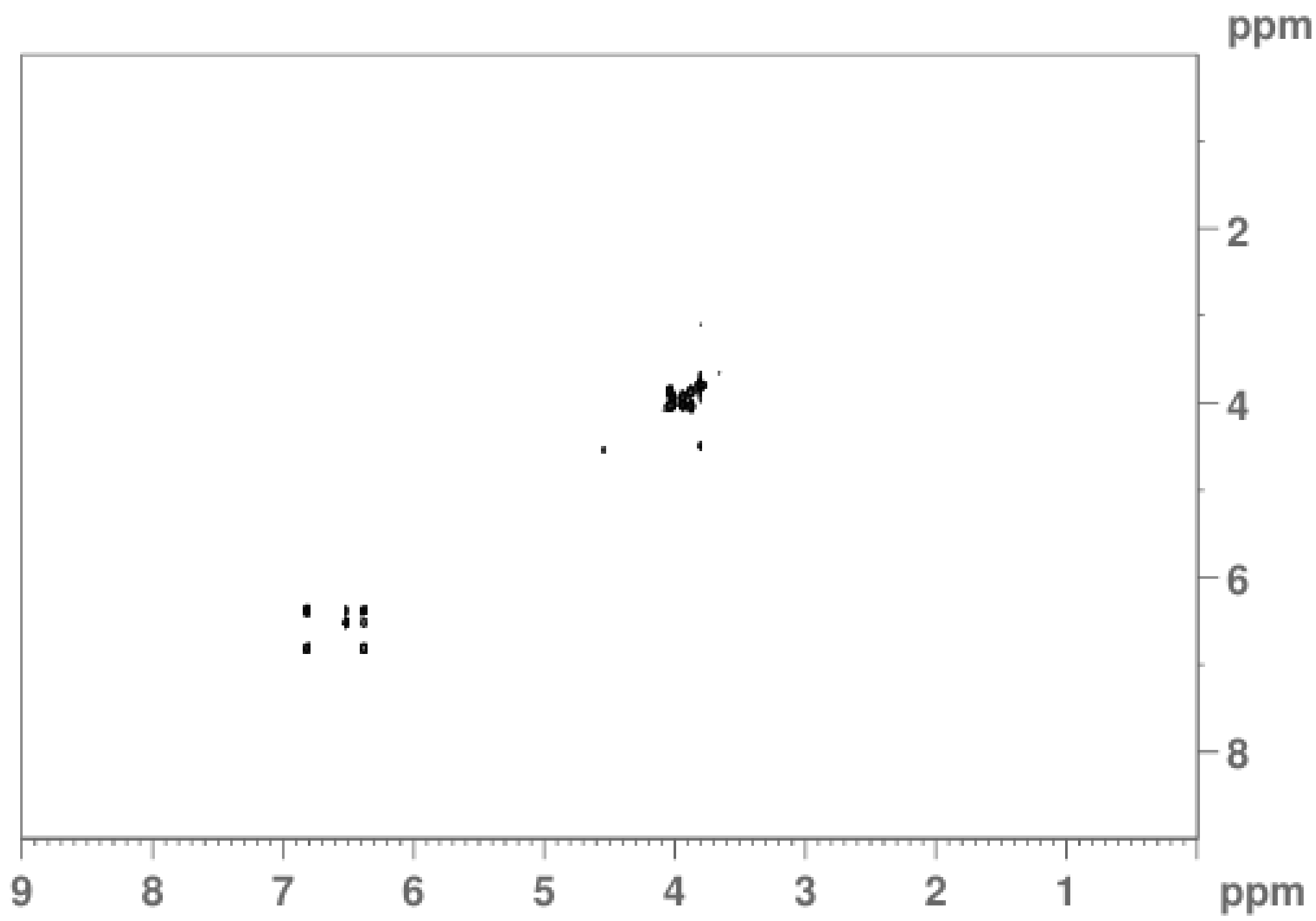


**Figure S5**. COSY spectrum of monomer **Q** in $CDCl_3$.

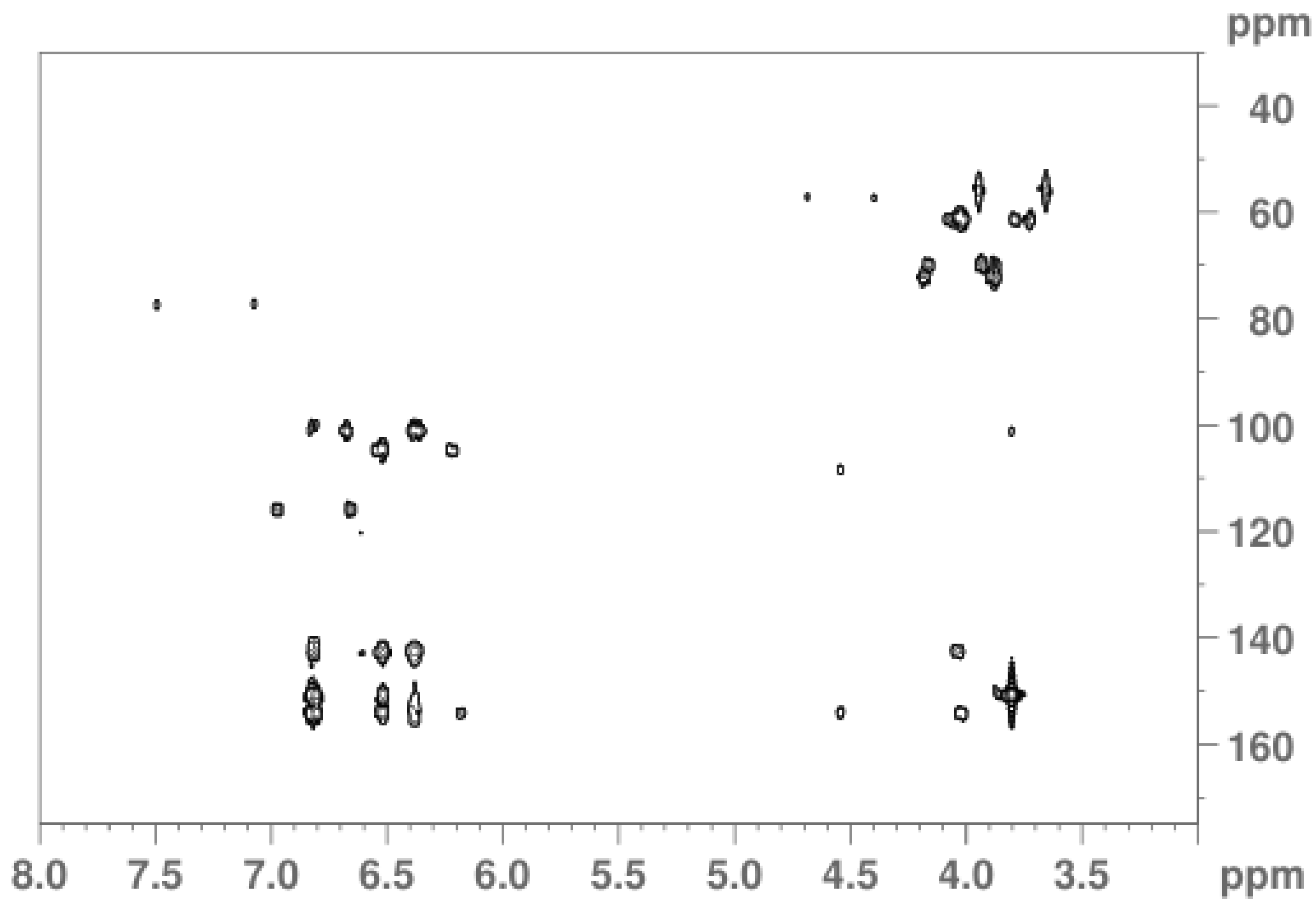


**Figure S6**. HMBC spectrum of monomer **Q** in $CDCl_3$.

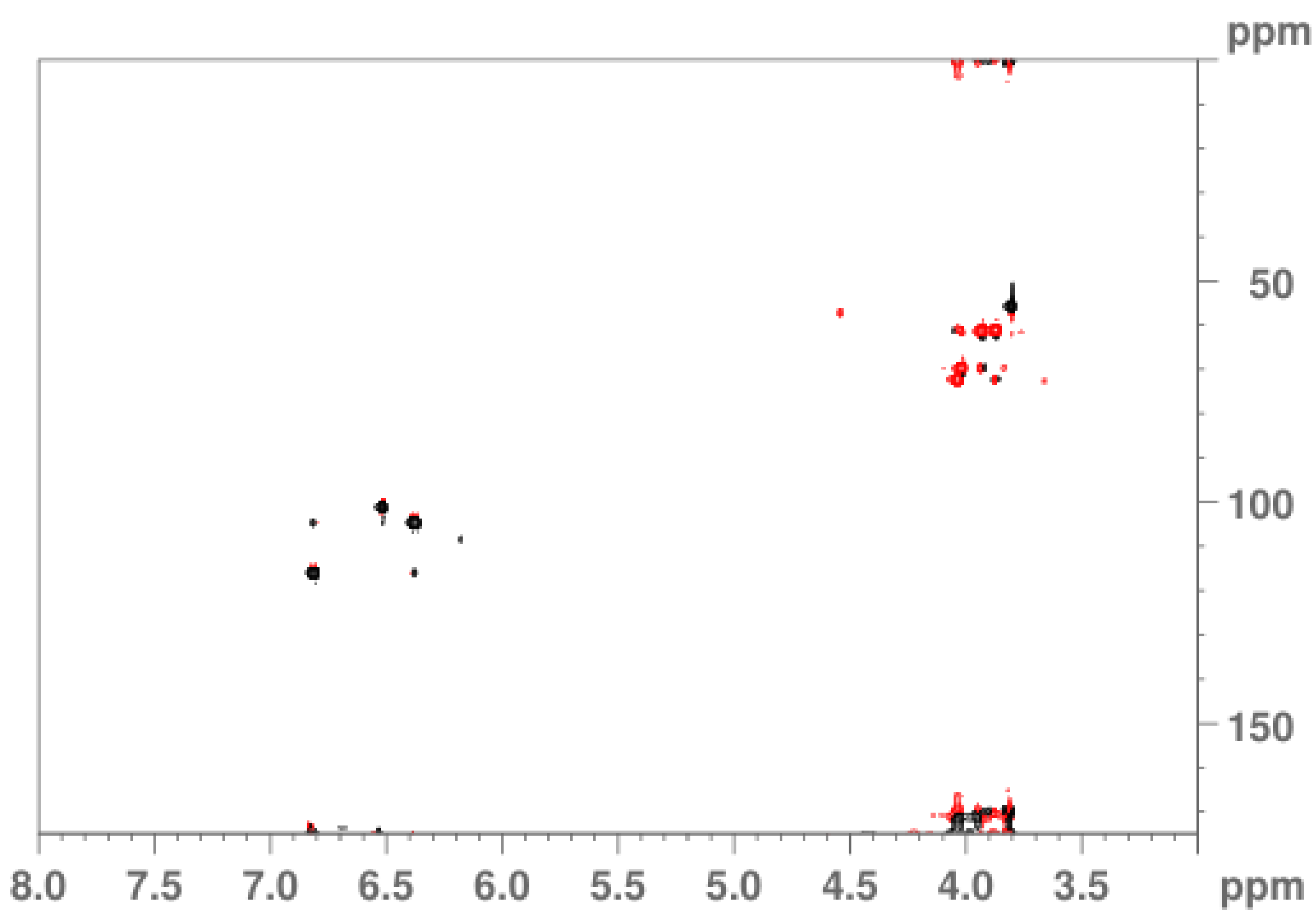


**Figure S7**. HSQC spectrum of monomer **Q** in $CDCl_3$.

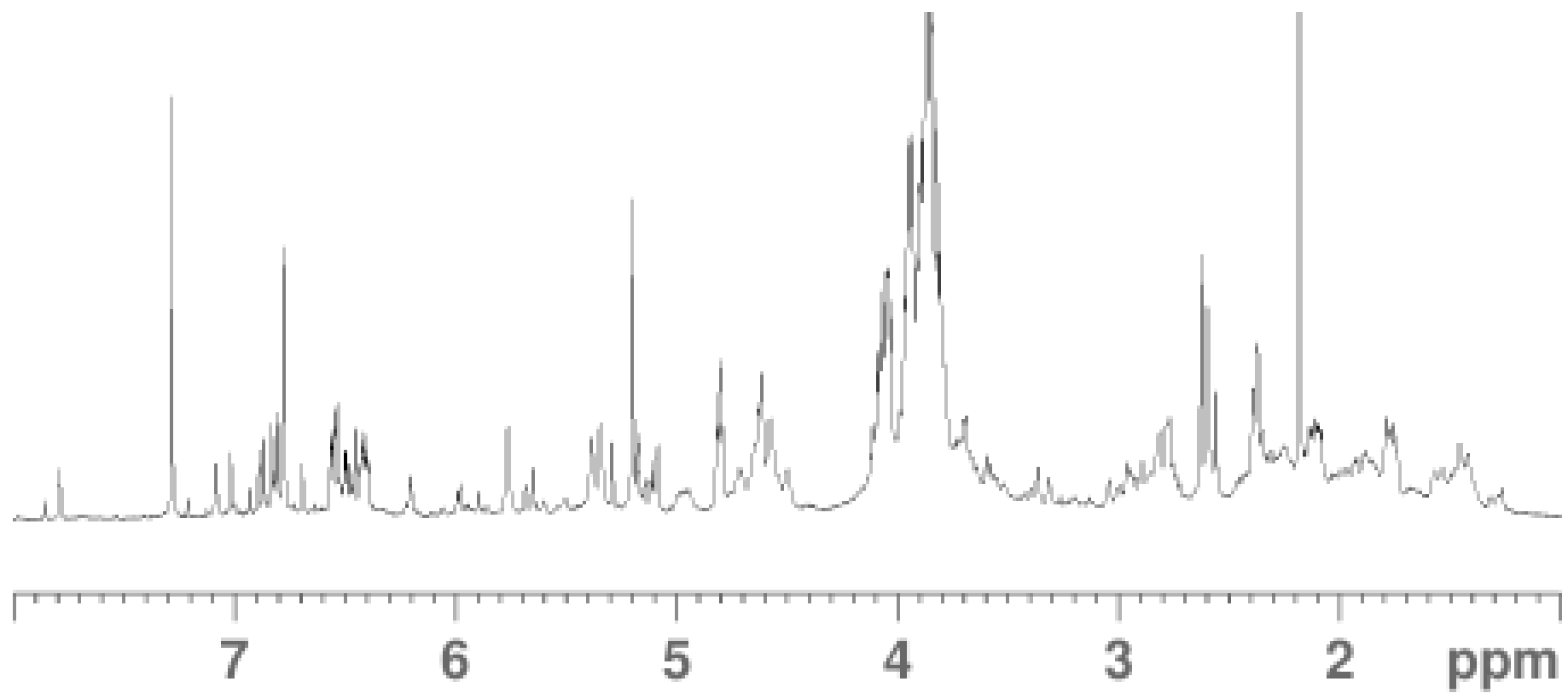


**Figure S8.** $^{1}$H-NMR ($CDCl_3$) of the product obtained from the reaction of 2-methoxyhydroquinone and ethylene carbonate in cyrene.

(A)

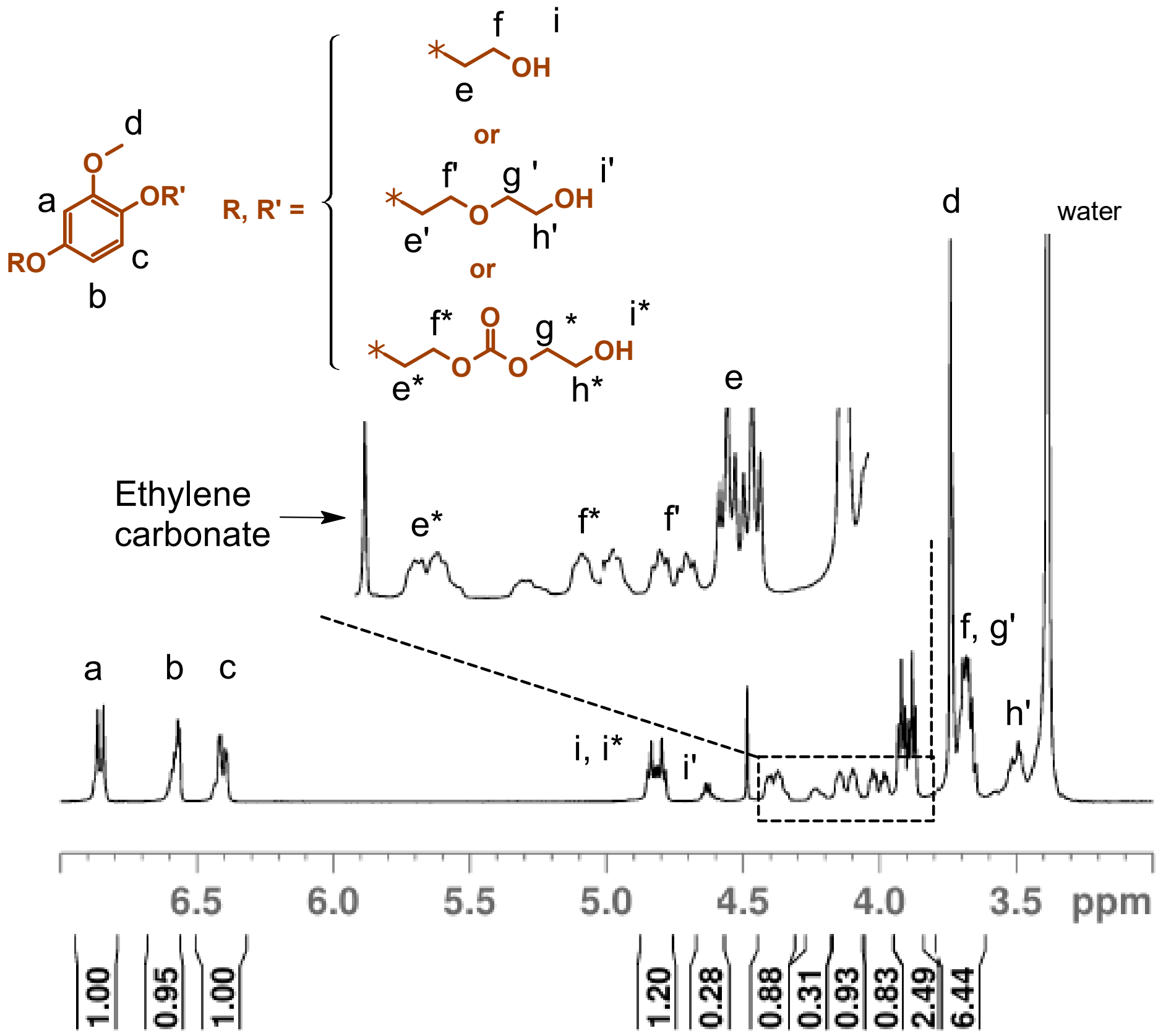


(B)

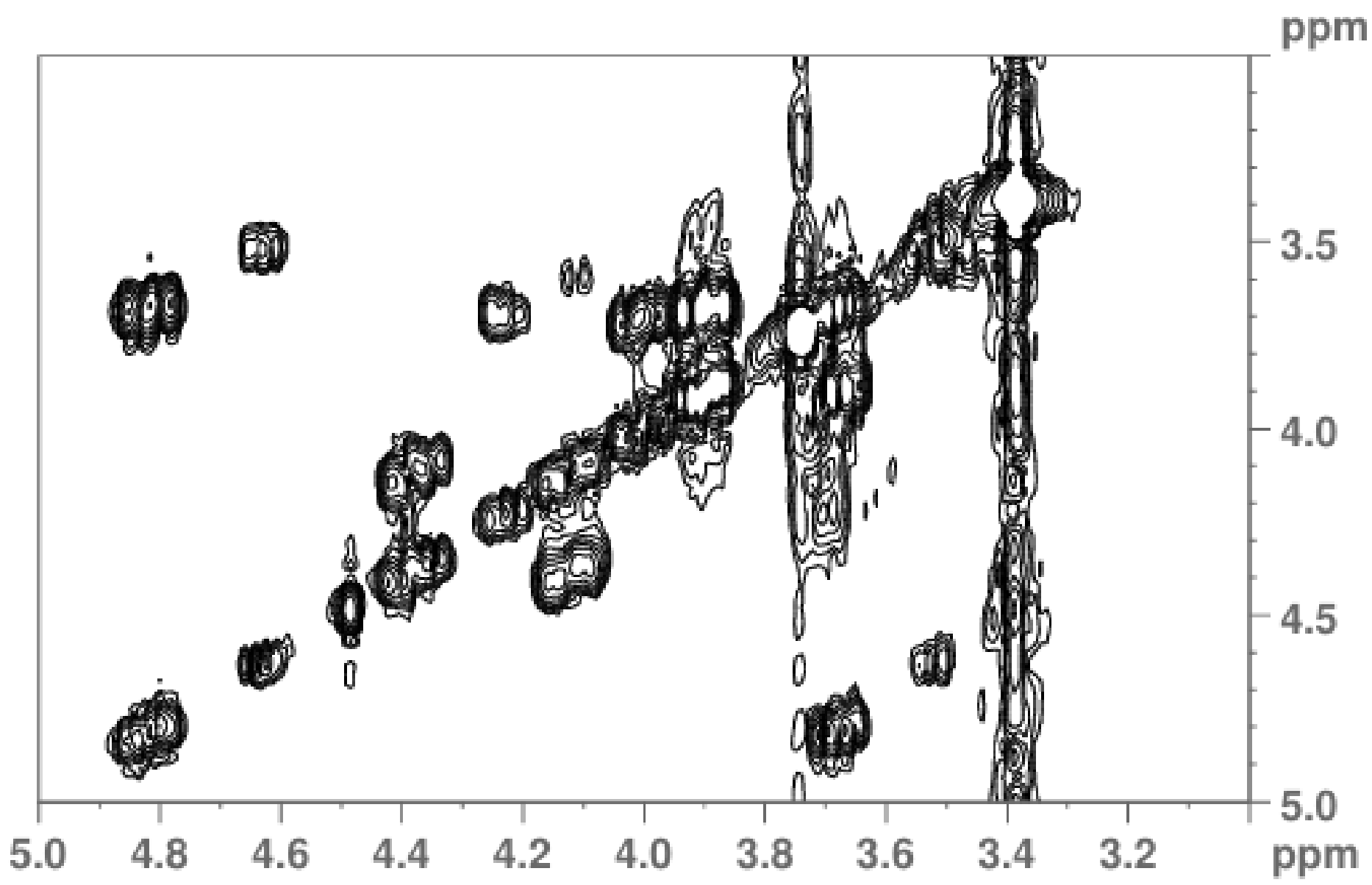


**Figure S9. (A)** $^{1}$H-NMR and **(B)** COSY spectra (DMSO-d$_6$) of monomer **Q** and 2-methoxyhydroquinone-based diols obtained from the neat reaction of 2-methoxyhydroquinone and ethylene carbonate.

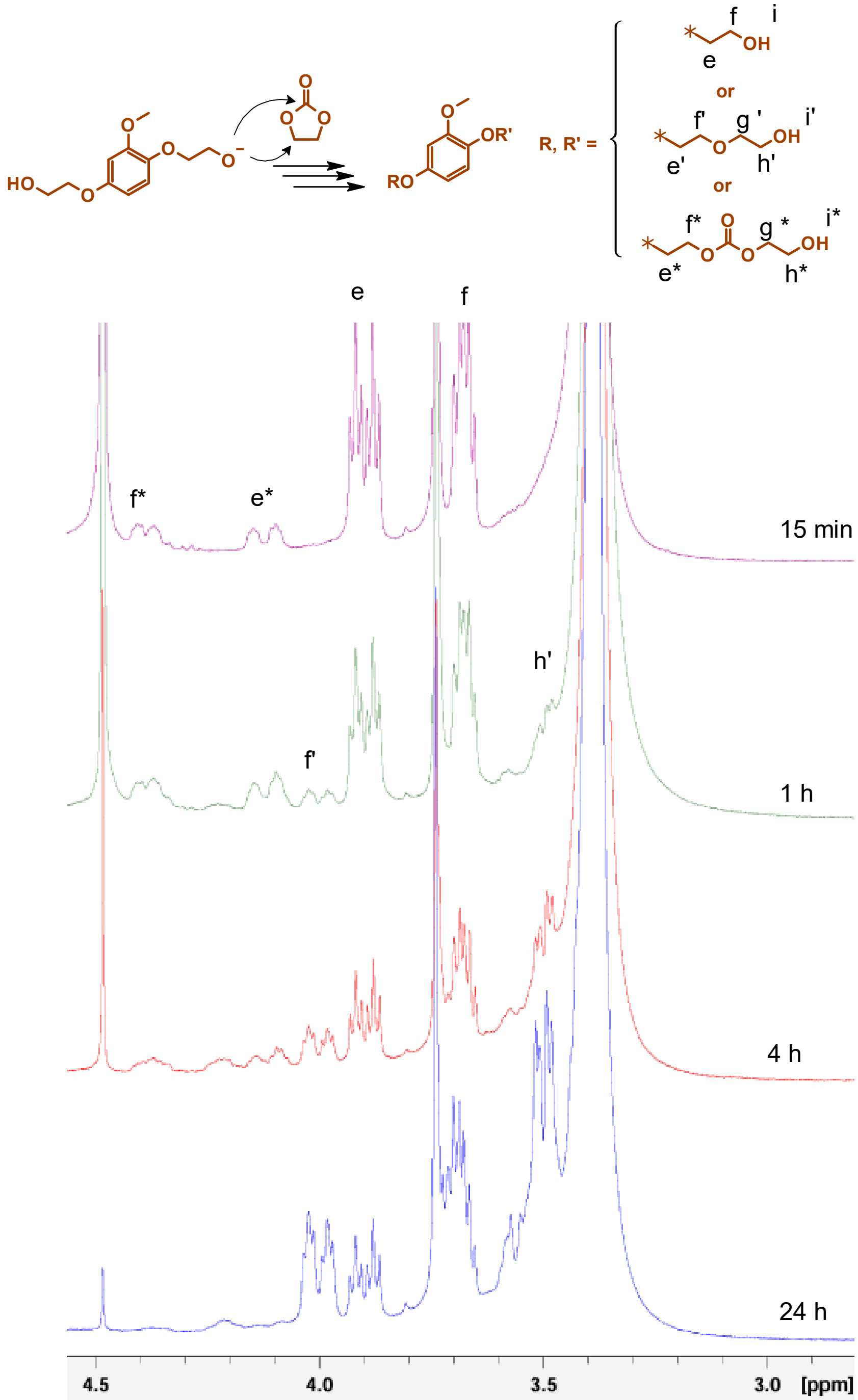


**Figure S10**. $^{1}$H-NMR and (DMSO-d6) of aliquots from the net hydroxyethylation of 2-methoxyquinone taken at different times.

### 3. Characterization of PU – $^{1}$H-NMR spectra

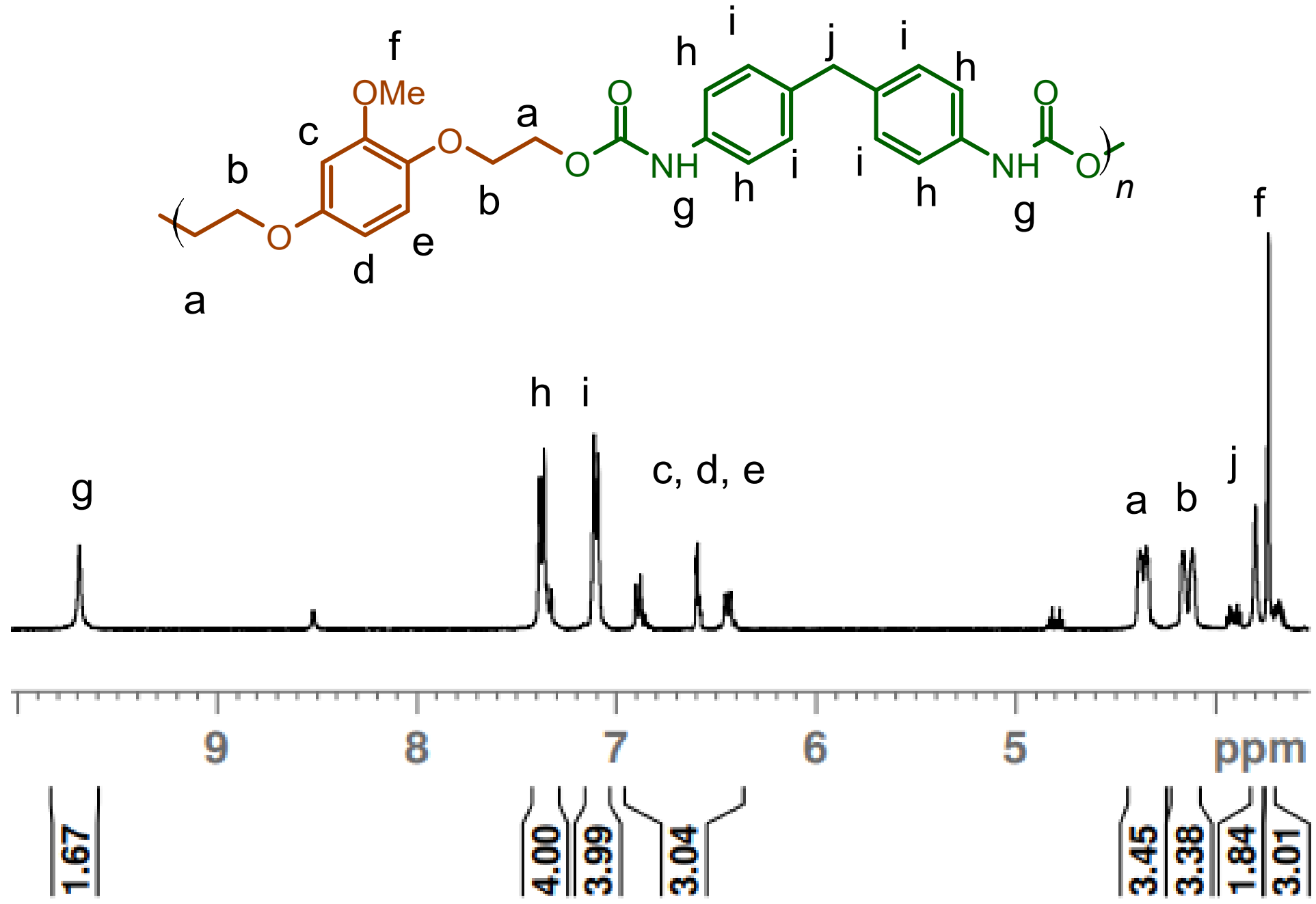


**Figure S11.** $^{1}$H-NMR spectrum of **P(Q-M)** in DMSO-d$_6$.

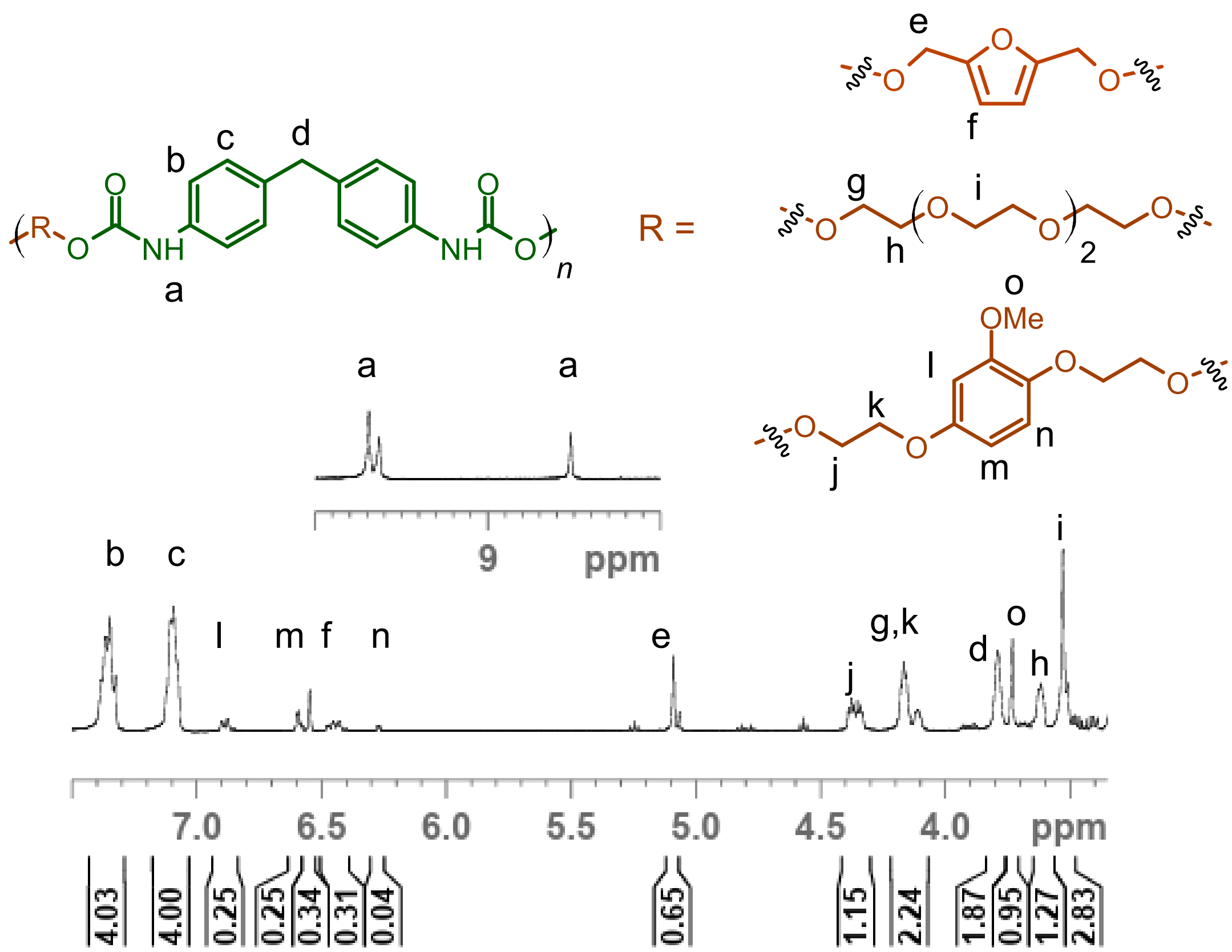


**Figure S12.** $^{1}$H-NMR spectrum of **P($Q_{35}T_{41}F_{19}$-M)** in DMSO-d$_6$.

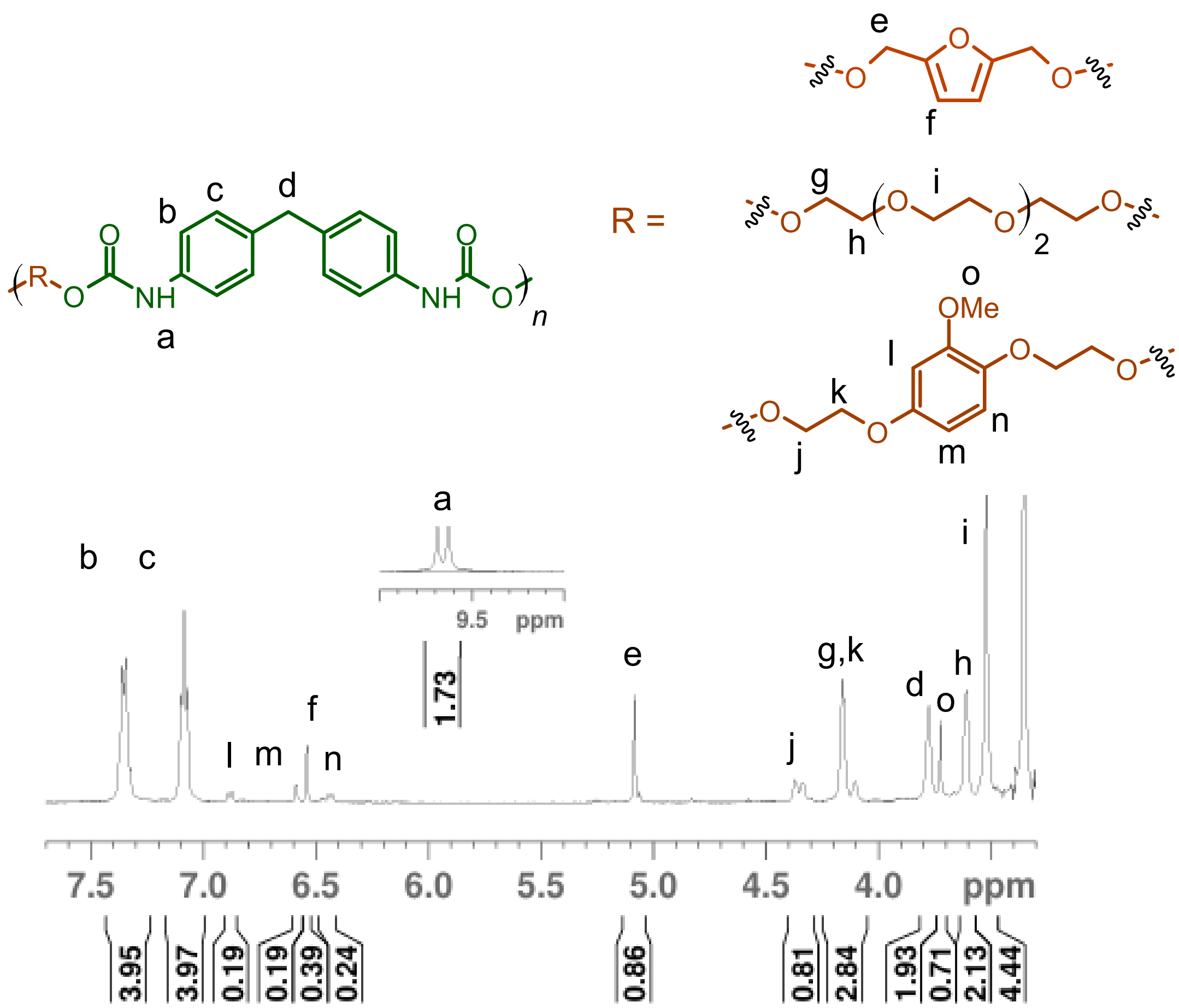


**Figure S13.** $^1$H-NMR spectrum of **P(Q$_{21}$T$_{58}$F$_{21}$-M)** in DMSO-$d_6$.

The molar ratio of each monomer on **P(Q$_{35}$T$_{41}$F$_{19}$-M)** and **P(Q$_{21}$T$_{58}$F$_{21}$-M)** was calculated according to Equations S1-3:

$$x = H_l/(H_l + \frac{H_f}{2} + \frac{H_i}{8}) \quad \textbf{Equation S2}$$

$$y = (H_i/8)/(H_l + \frac{H_f}{2} + \frac{H_i}{8}) \quad \textbf{Equation S3}$$

$$z = (H_f/2)/(H_l + \frac{H_f}{2} + \frac{H_i}{8}) \quad \textbf{Equation S4}$$

Where $x$, $y$ and $z$ represent the molar ration of monomer **Q**, TEG and BHF, respectively, and $H_l$, $H_f$, $H_i$, $H_b$ represent the area of the peaks corresponding to protons l, f, I and b in Figures S6 and S7.

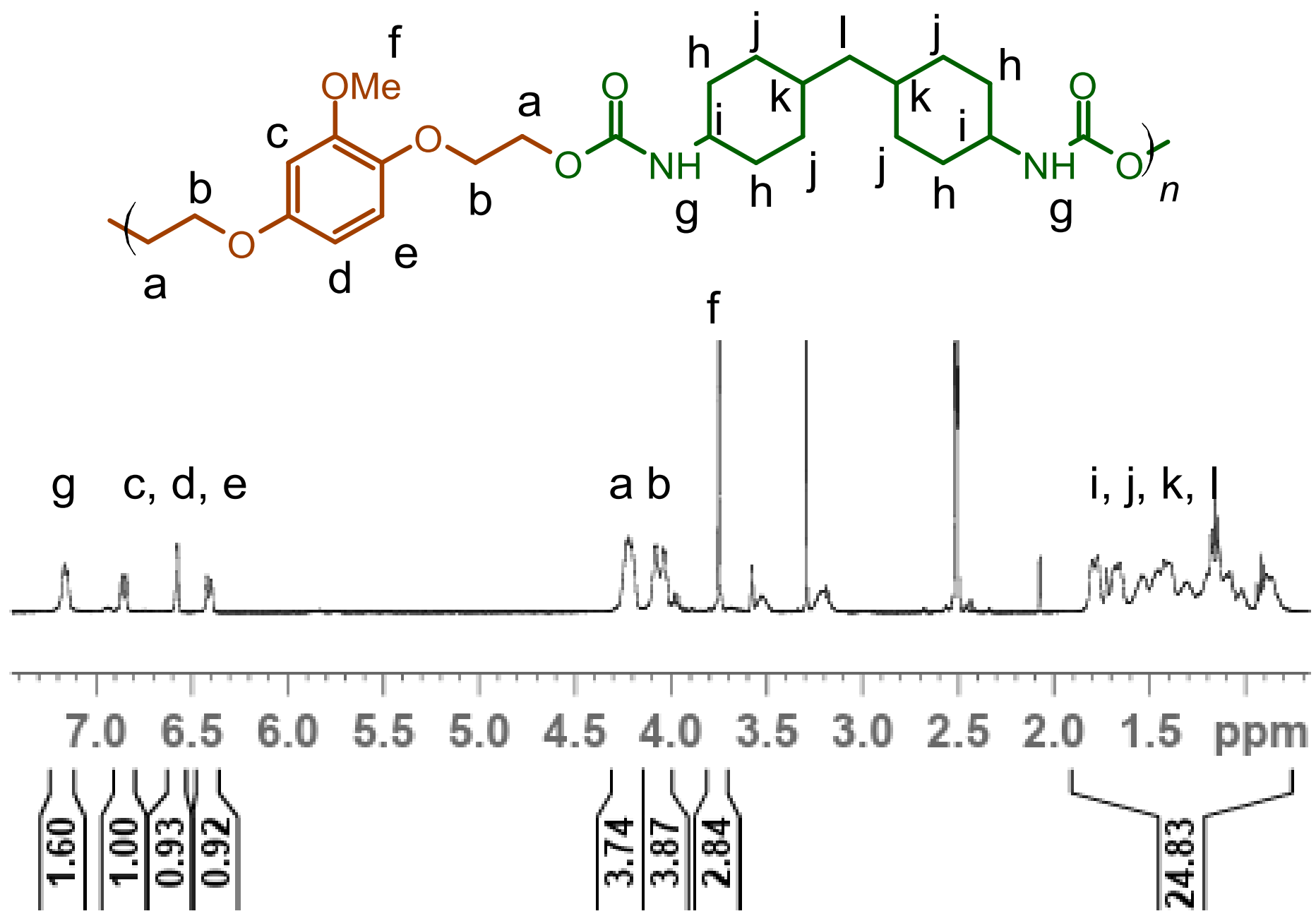


**Figure S14.** $^{1}$H-NMR spectrum of **P(Q-H)** in DMSO-d$_6$.

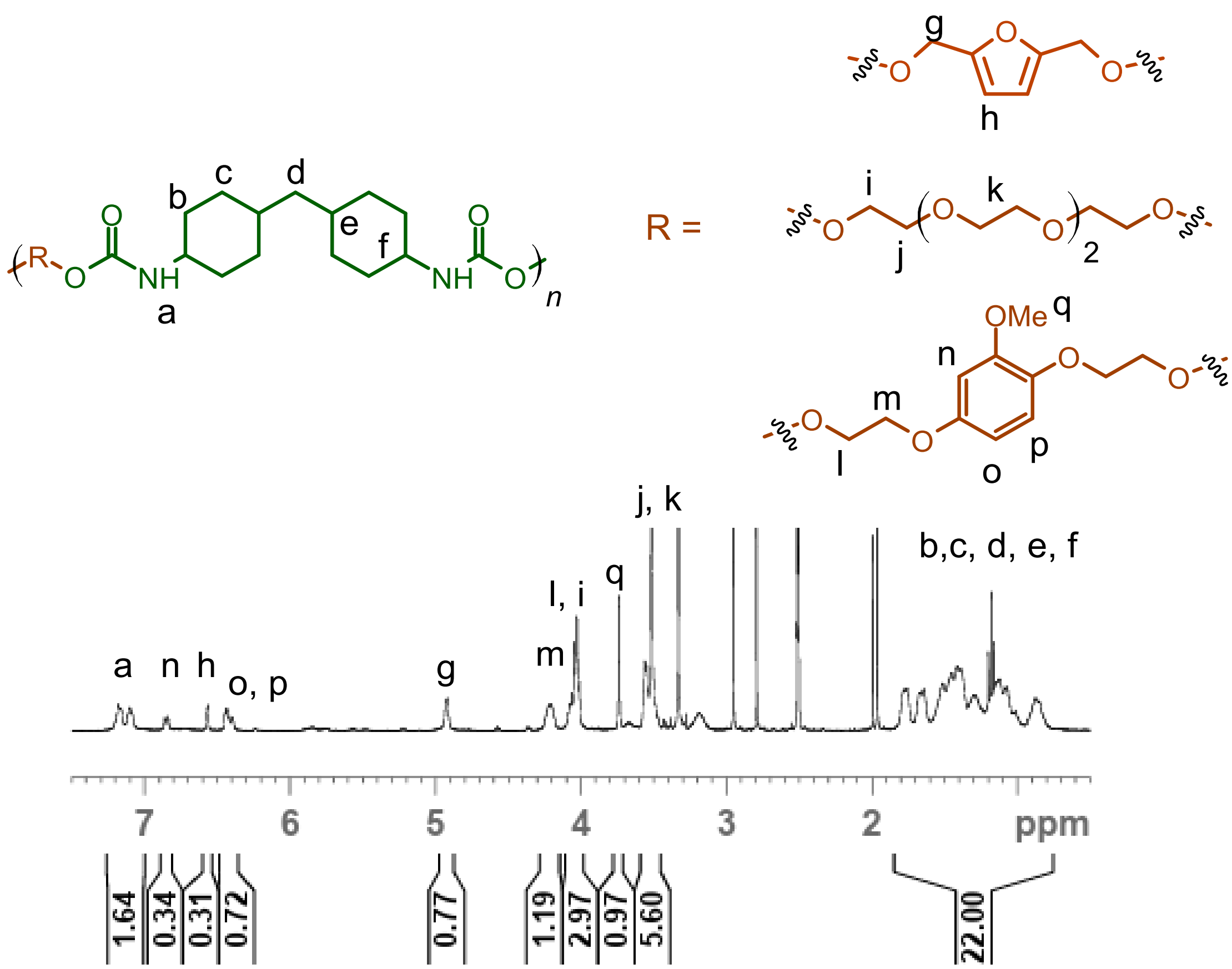


**Figure S15.** $^{1}$H-NMR spectrum of **P(Q$_{35}$T$_{49}$F$_{16}$-H)** in DMSO-d$_6$.

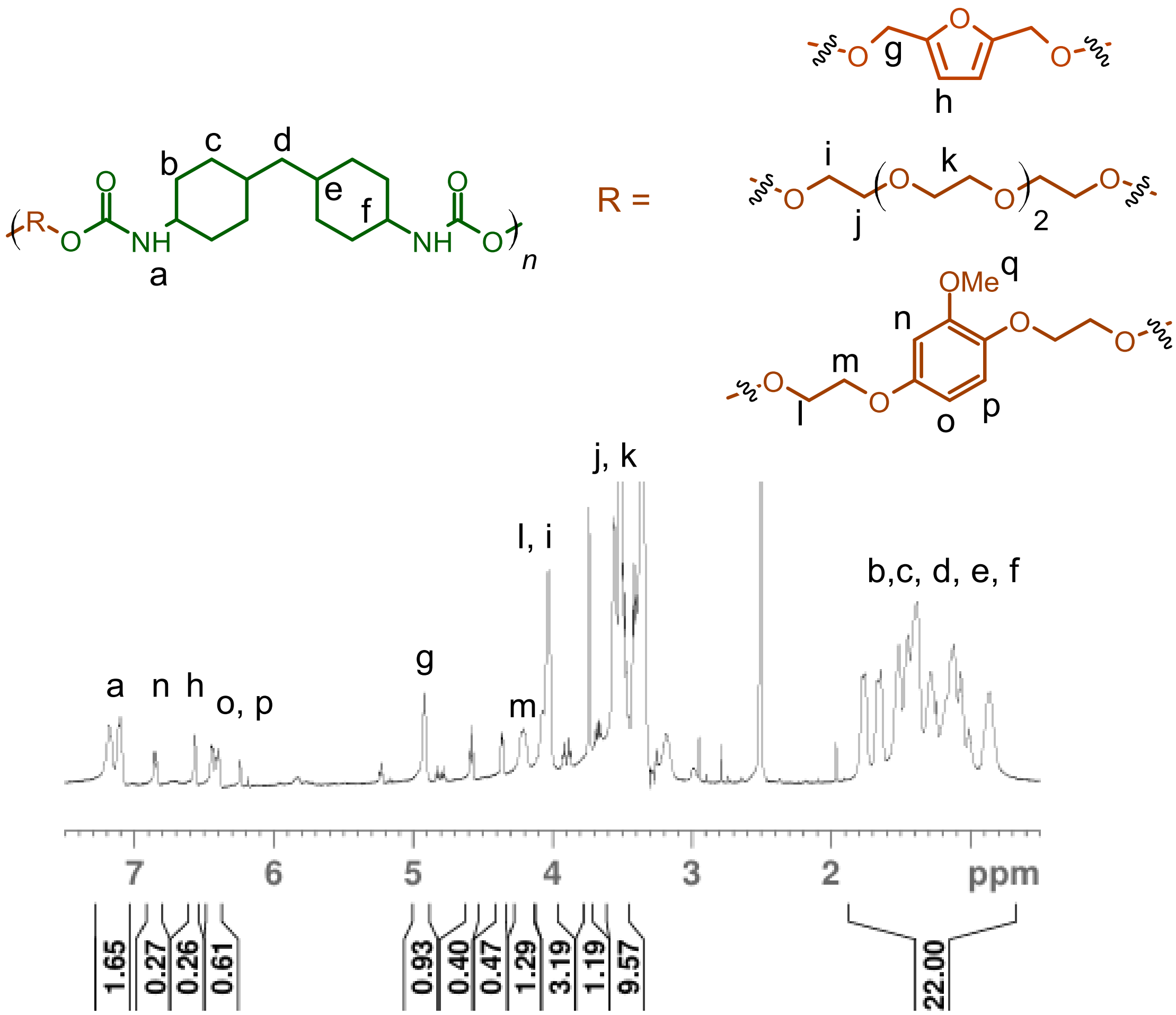


**Figure S16.** $^{1}$H-NMR spectrum of **P($Q_{23}T_{62}F_{15}$-H)** in DMSO-d$_6$.

The molar ratio of each monomer on **P($Q_{35}T_{49}F_{16}$-H)** and **P($Q_{23}T_{62}F_{15}$-H)** was calculated according to Equations S4-6:

$$x = H_n/(H_n + \frac{H_h}{2} + \frac{H_{j,k}}{12}) \quad \textbf{Equation S5}$$

$$y = (\frac{H_{j,k}}{12})/(H_n + \frac{H_h}{2} + \frac{H_{j,k}}{12}) \quad \textbf{Equation S6}$$

$$z = (\frac{H_h}{2})/(H_n + \frac{H_h}{2} + \frac{H_{j,k}}{12}) \quad \textbf{Equation S7}$$

Where $x$, $y$ and $z$ represent the molar ration of monomer **Q**, TEG and BHF, respectively, and $H_n$, $H_h$, $H_{j,k}$, $H_{b-f}$ represent the area of the peaks corresponding to protons n, h, j-k and b-f in Figures S10 and S11.

**4. Synthesis and characterization of PU – $^{13}$C-NMR and FTIR spectra**

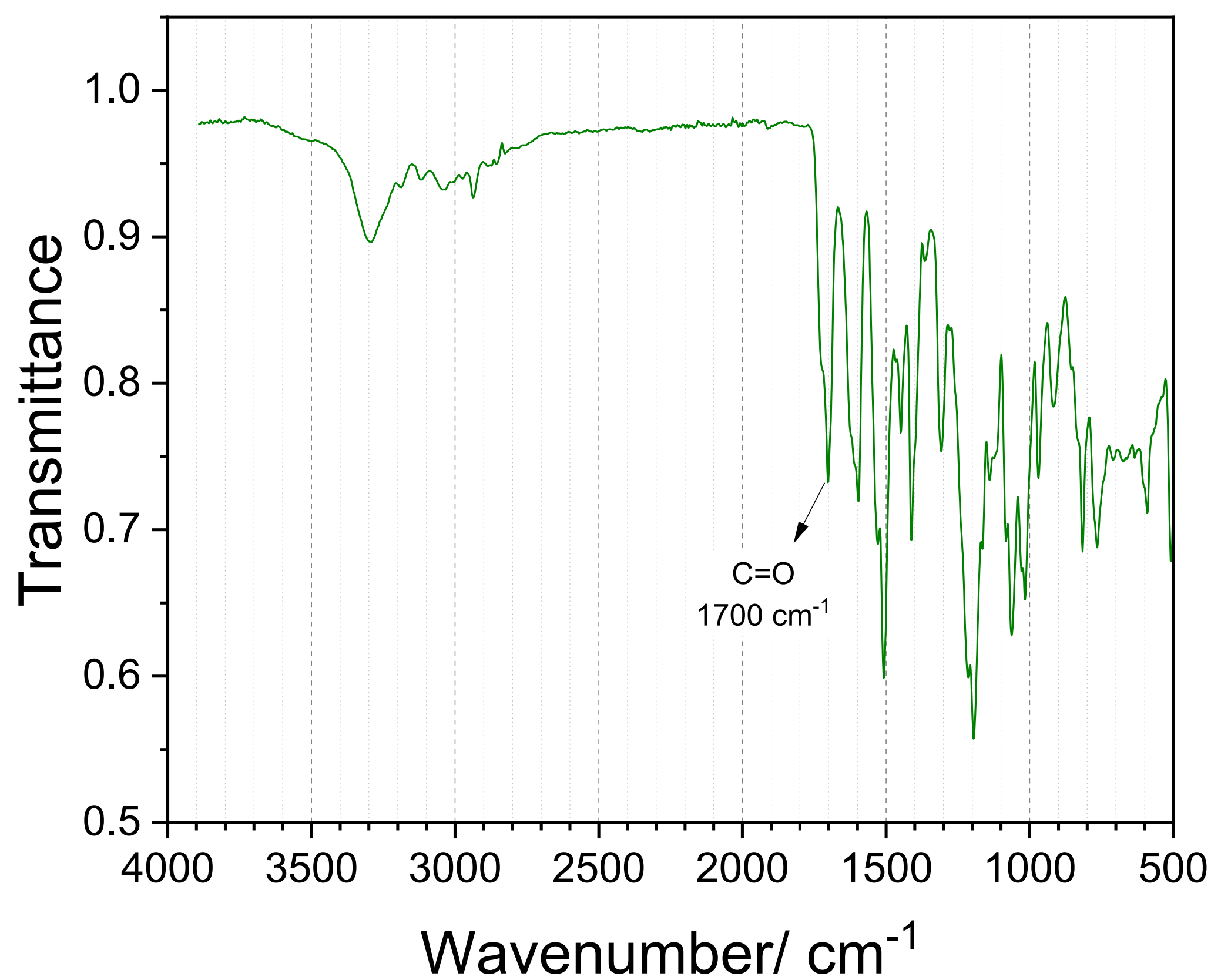


**Figure S17.** FTIR spectrum of **P(Q-M)** in DMSO-$d_6$.

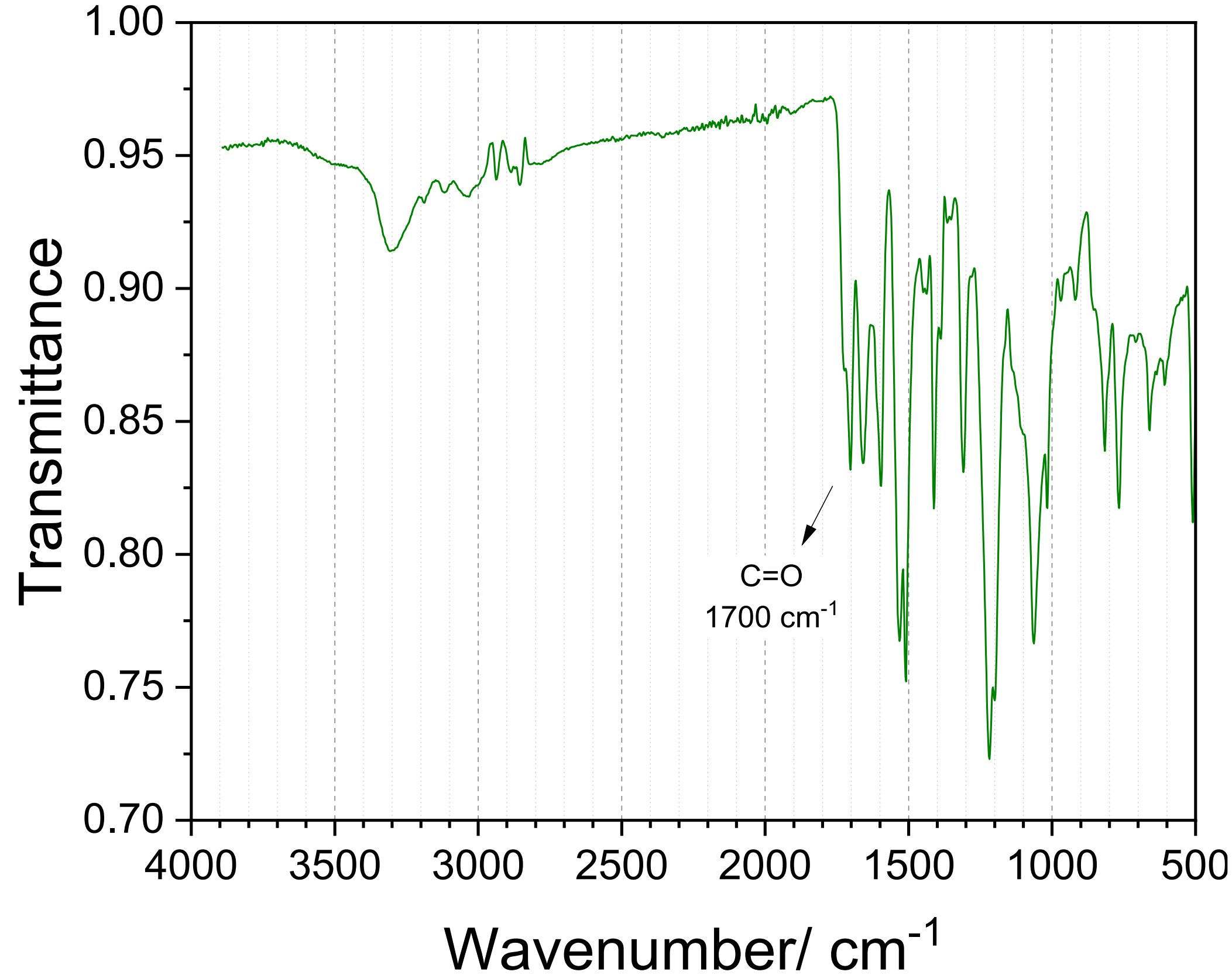


**Figure S18.** FTIR spectrum of **P($Q_{35}T_{41}F_{19}$-M)** in DMSO-$d_6$.

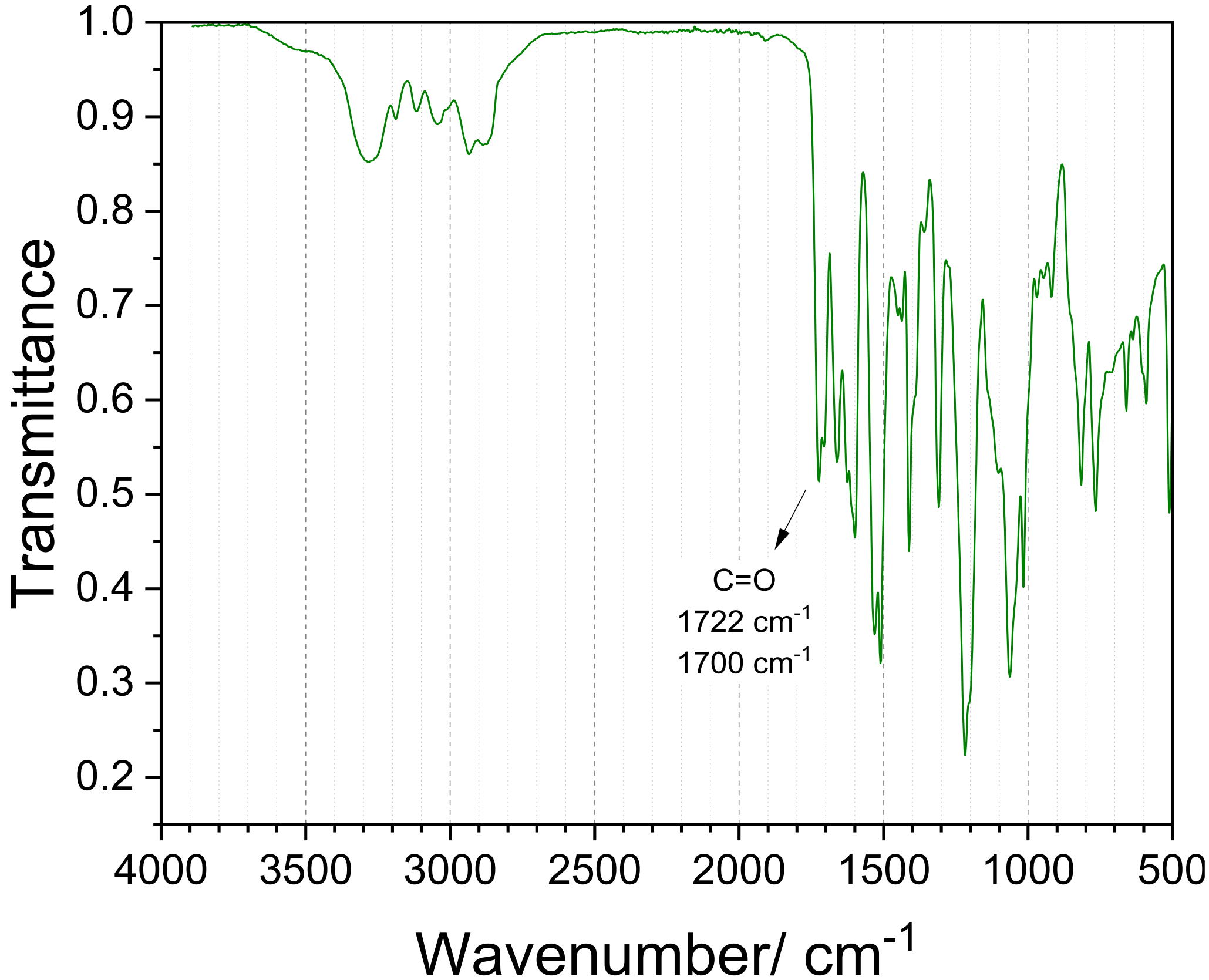


**Figure S19.** FTIR spectrum of **P($Q_{21}T_{58}F_{21}$-M)** in DMSO-$d_6$.

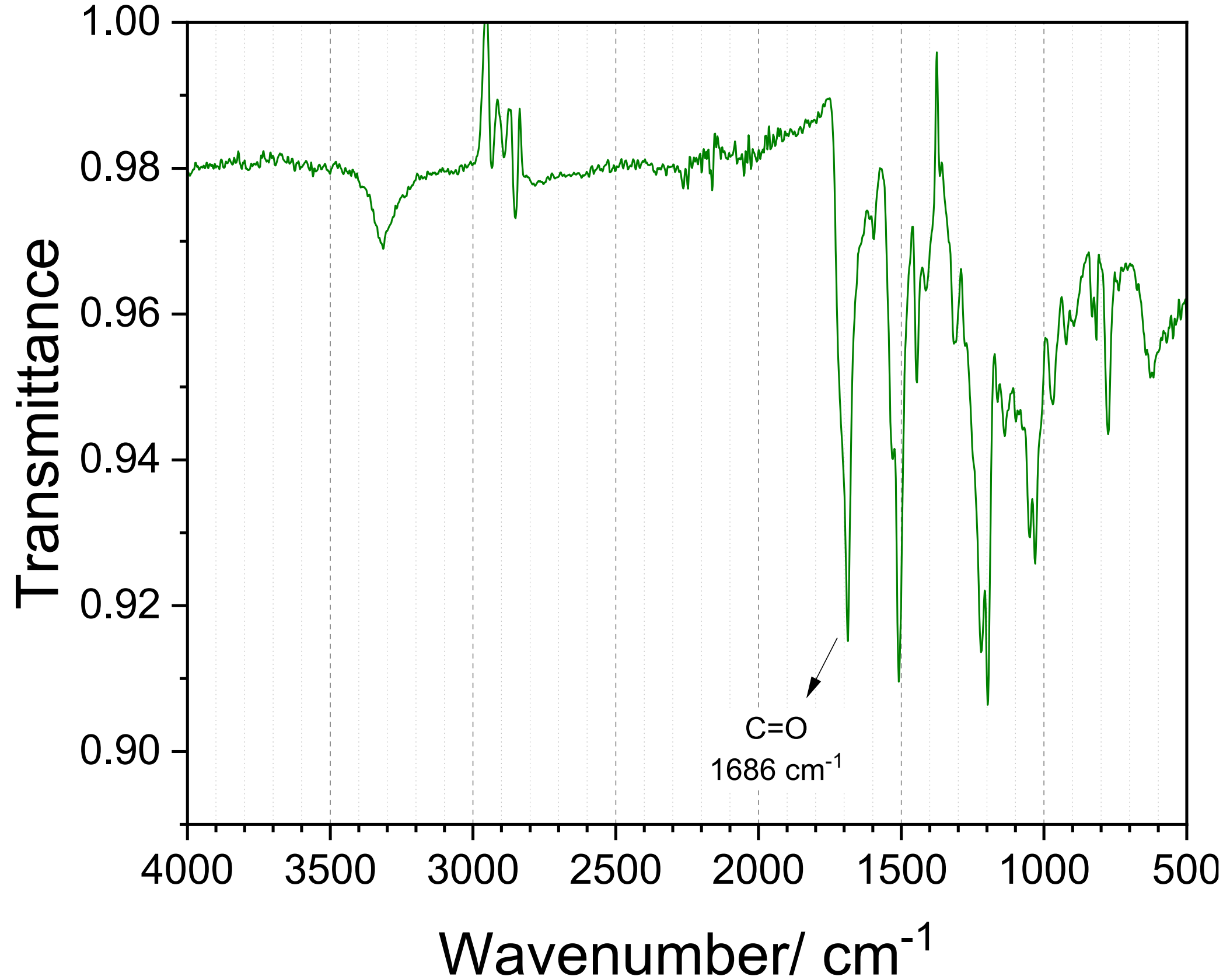


**Figure S20.** FTIR spectrum of **P(Q-H)** in DMSO-$d_6$.

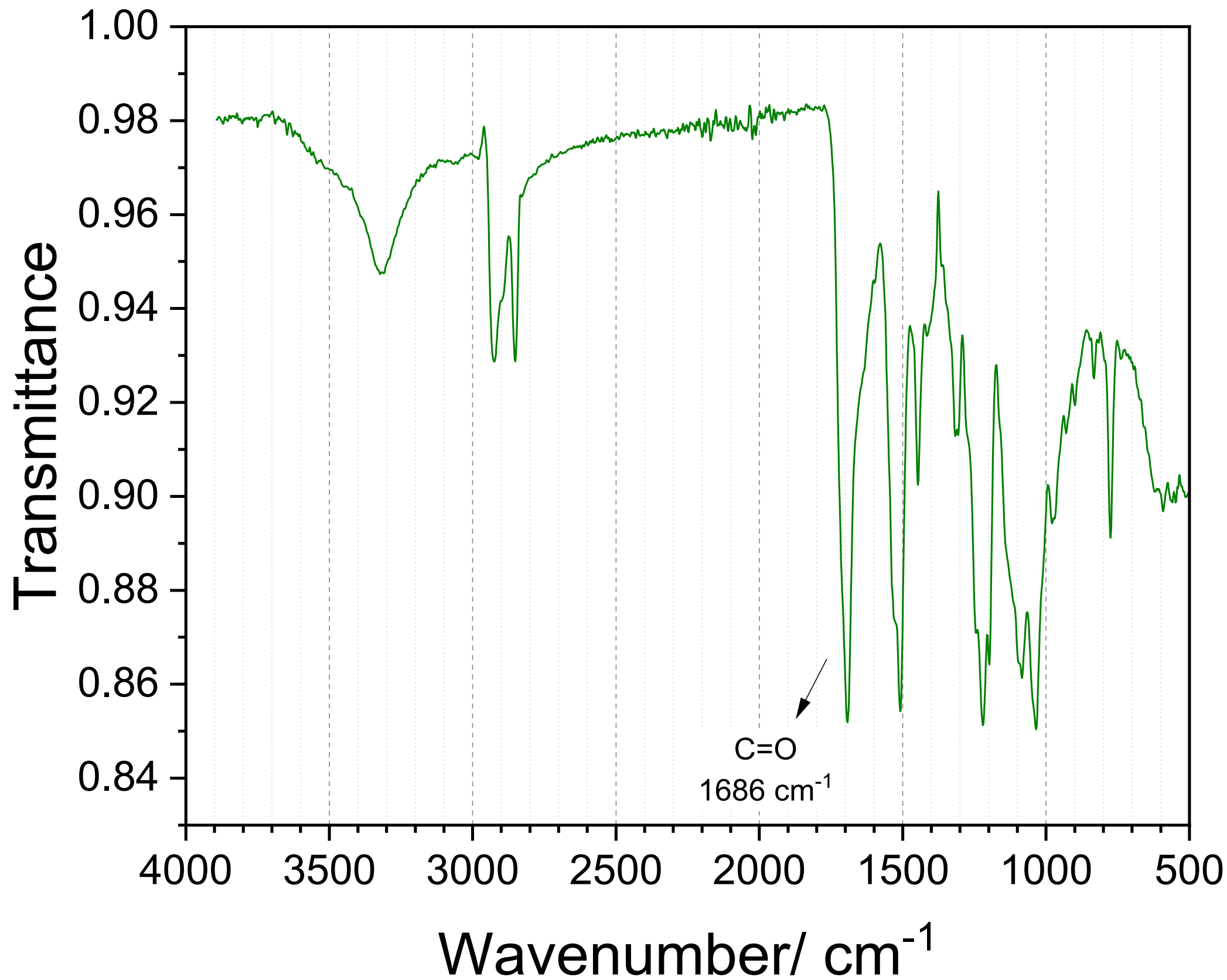


**Figure S21.** FTIR spectrum of **P($Q_{35}T_{49}F_{16}$-H)** in DMSO-$d_6$.

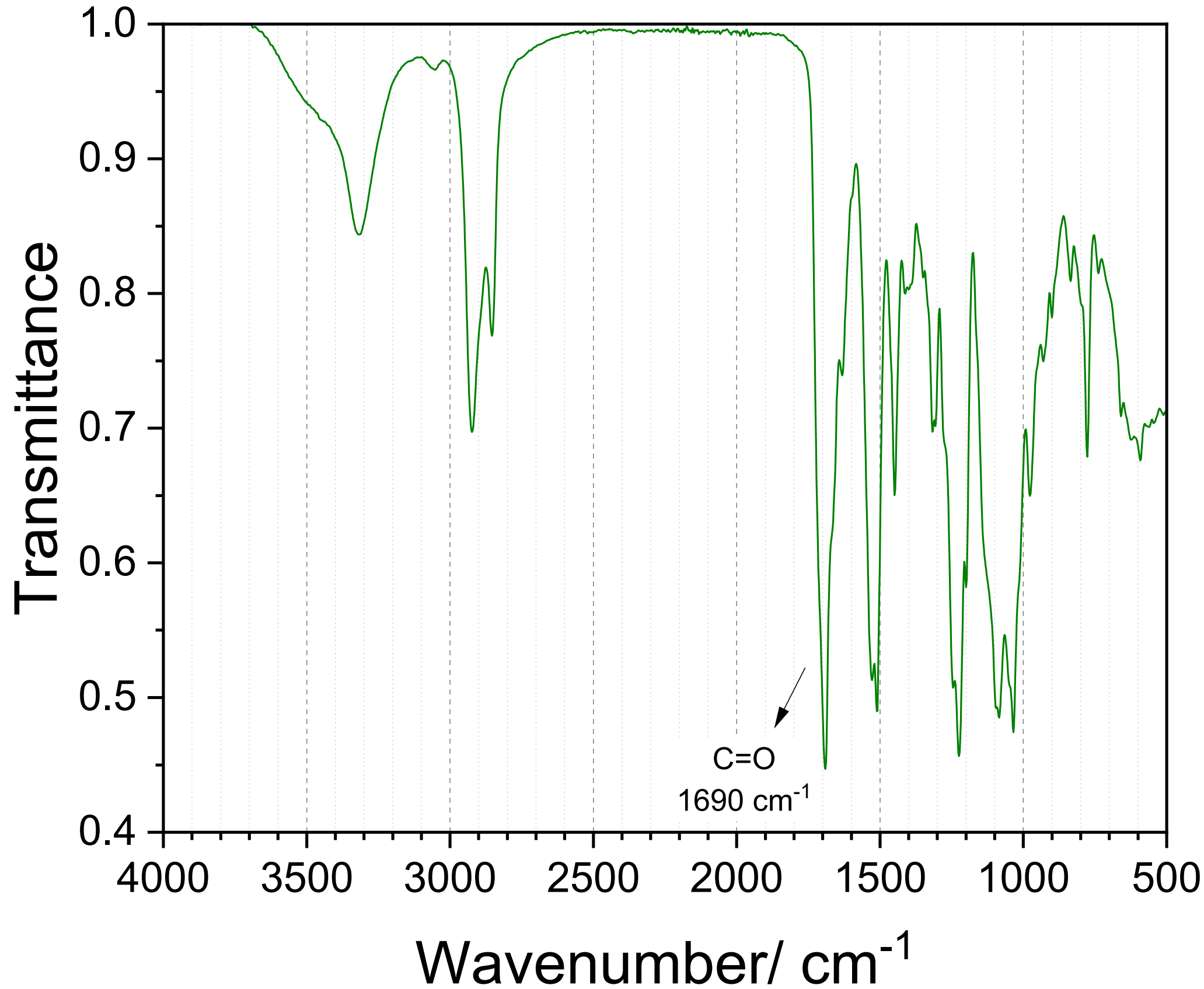


**Figure S22.** FTIR spectrum of **P($Q_{23}T_{62}F_{15}$-H)** in DMSO-$d_6$.

## 5. Synthesis and characterization of PU – GPC chromatograms

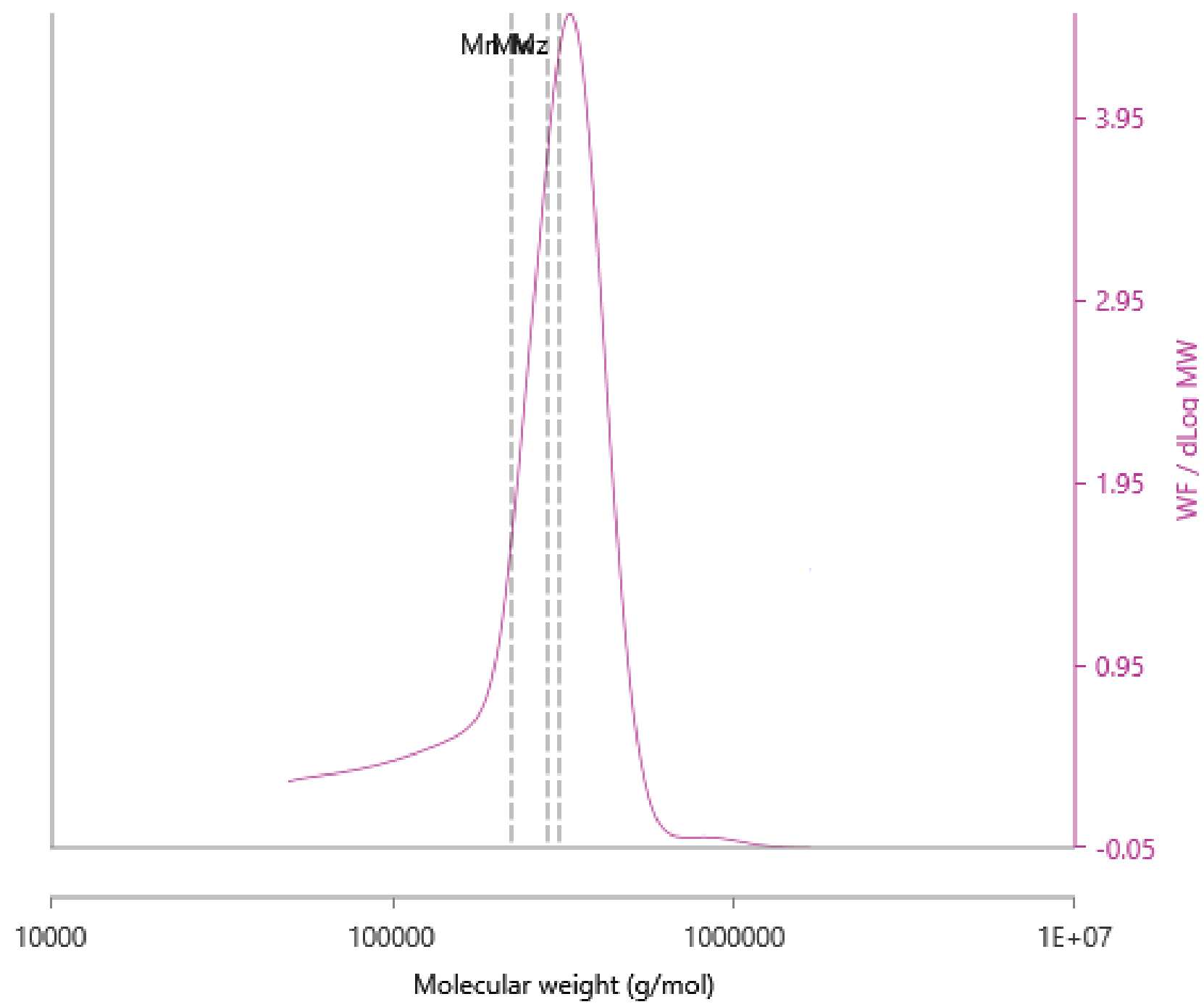


**Figure S23.** Gel permeation chromatogram of **P(Q-M)** in THF.

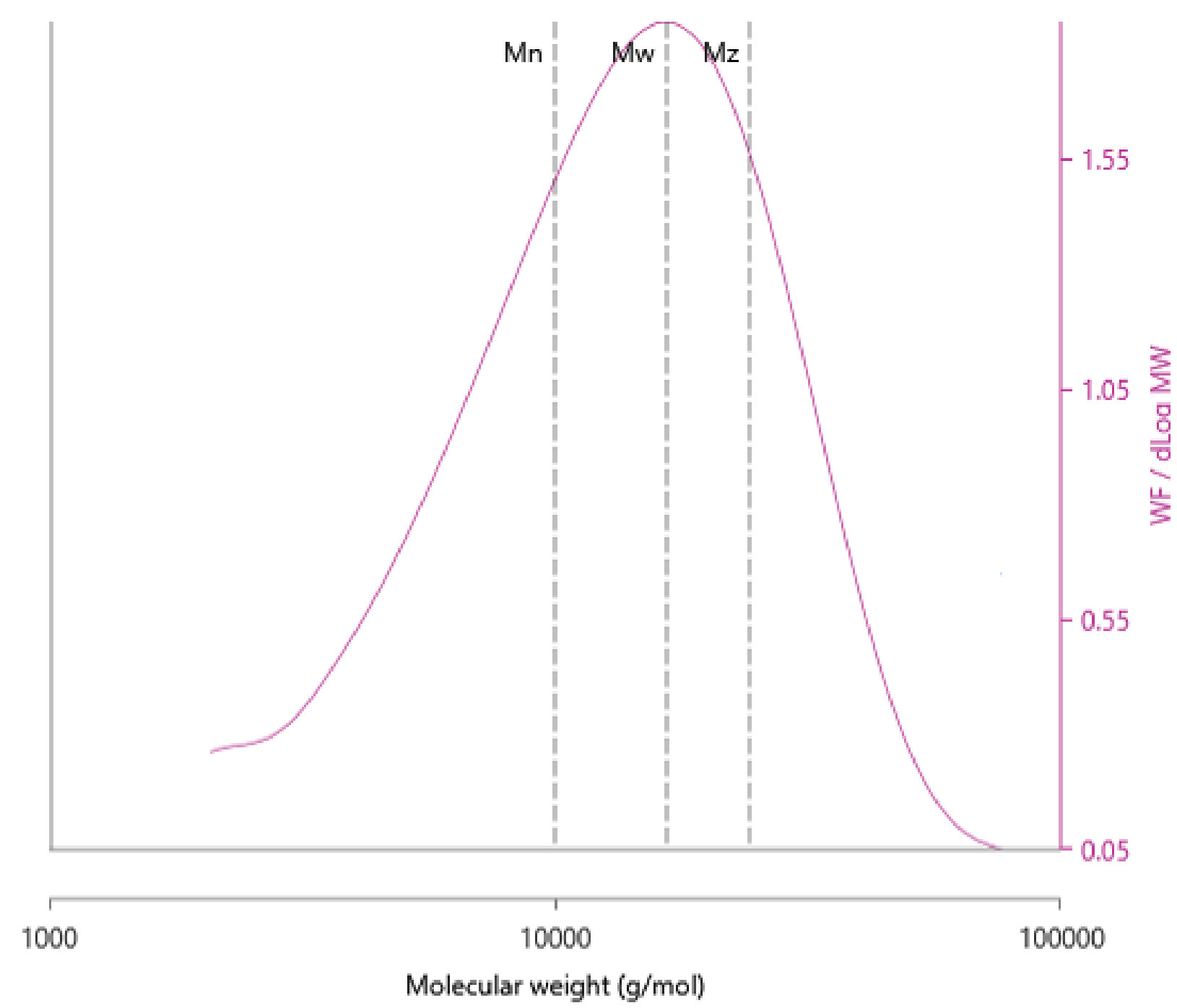


**Figure S24.** Gel permeation chromatogram of **P($Q_{35}T_{41}F_{19}$-M)** in THF.

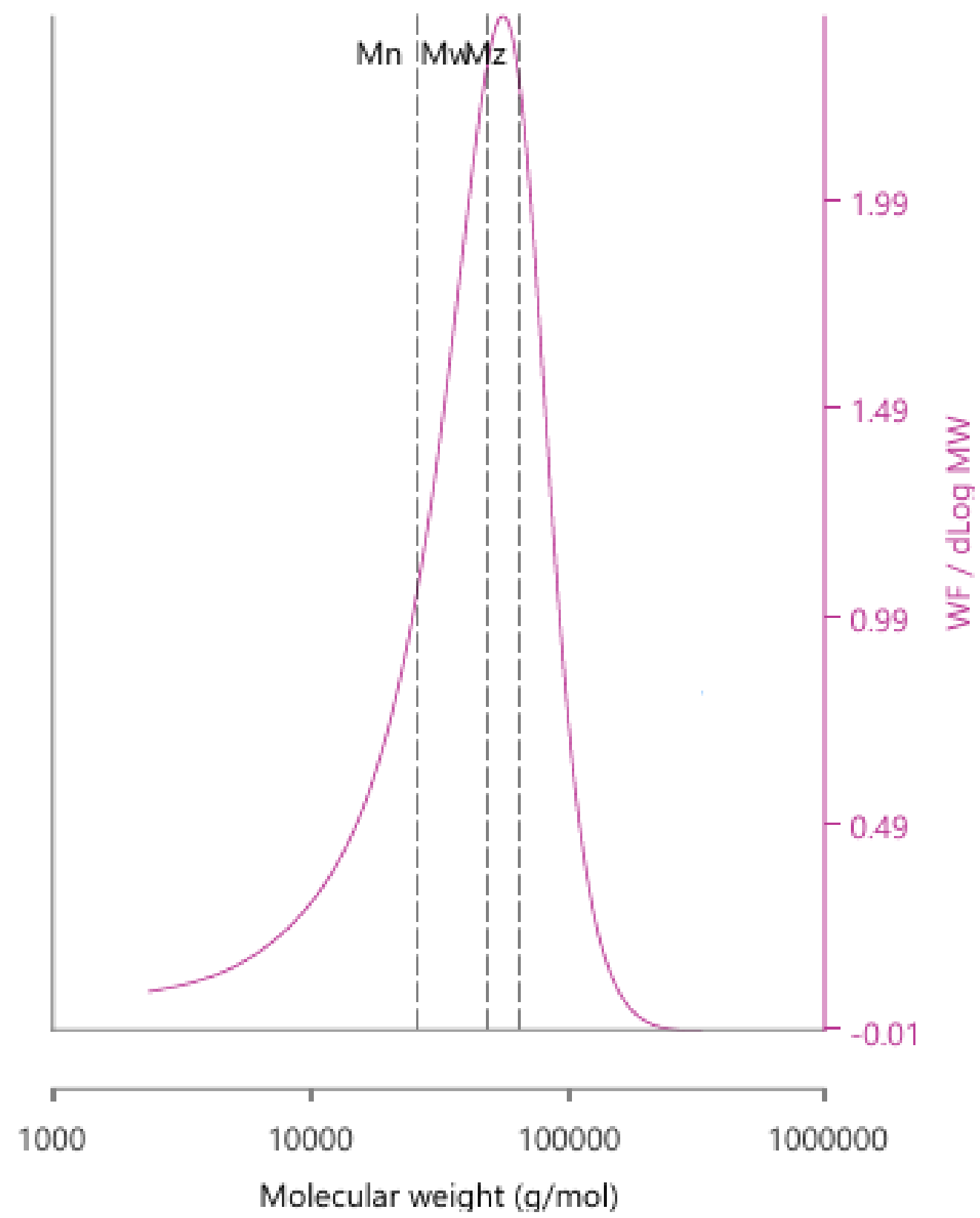


**Figure S25.** Gel permeation chromatogram of **P($Q_{21}T_{58}F_{21}$-M)** in THF.

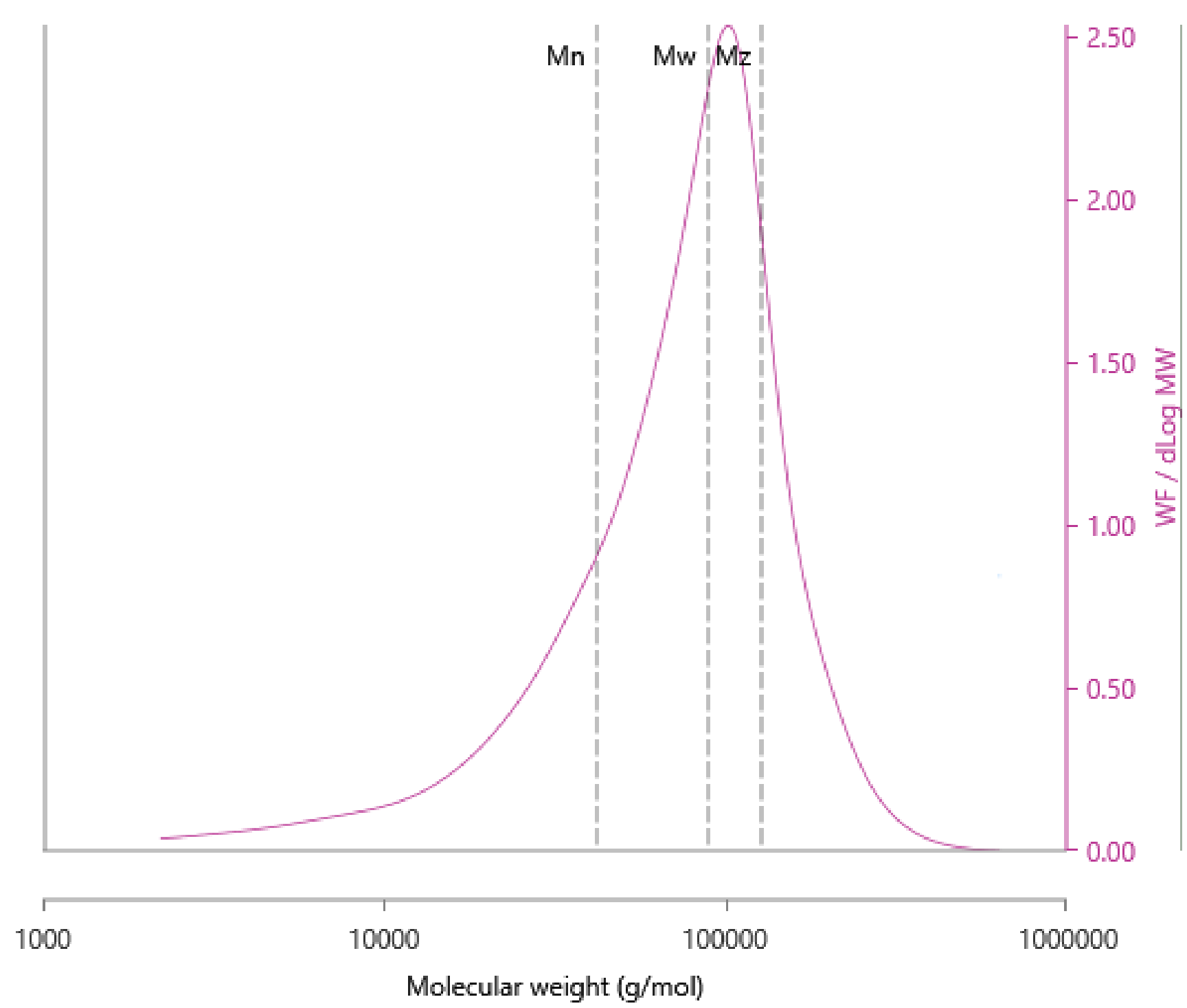


**Figure S26.** Gel permeation chromatogram of **P(Q-H)** in THF.

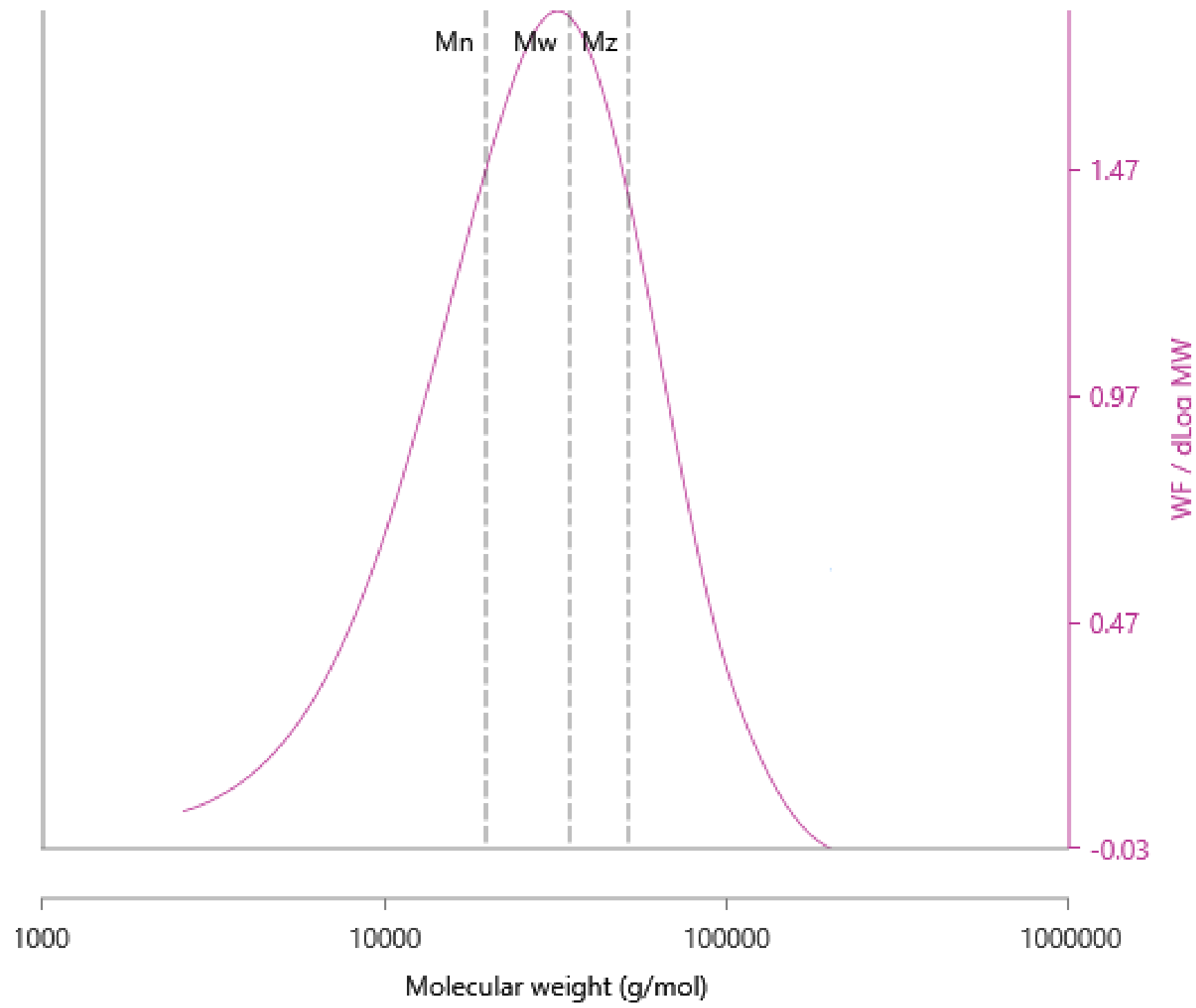


**Figure S27.** Gel permeation chromatogram of **P($Q_{35}T_{49}F_{16}$-H)** in THF.

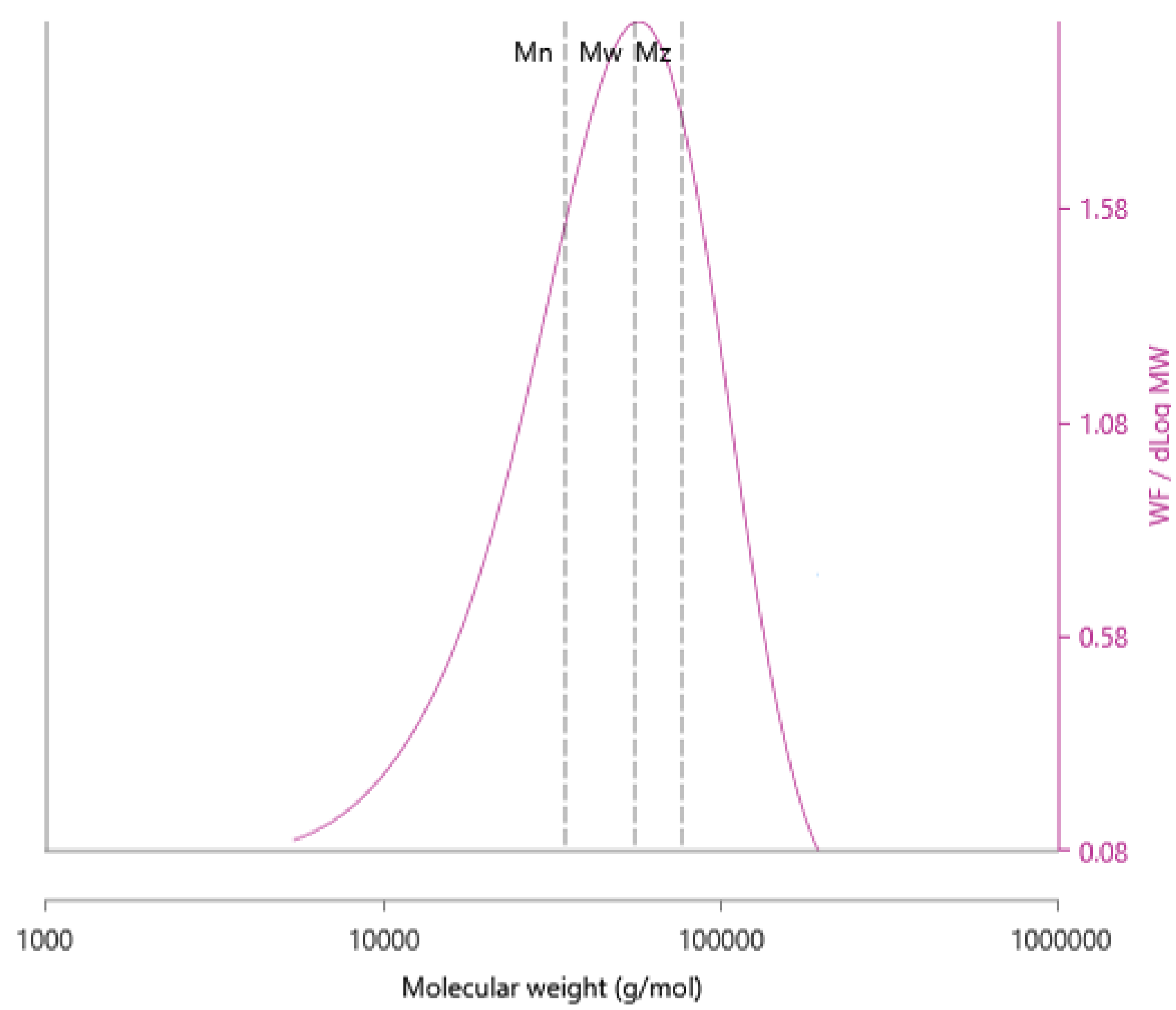


**Figure S28.** Gel permeation chromatogram of **P($Q_{23}T_{62}F_{15}$-H)** in THF.

## 6. Thermal characterization – DSC and TGA

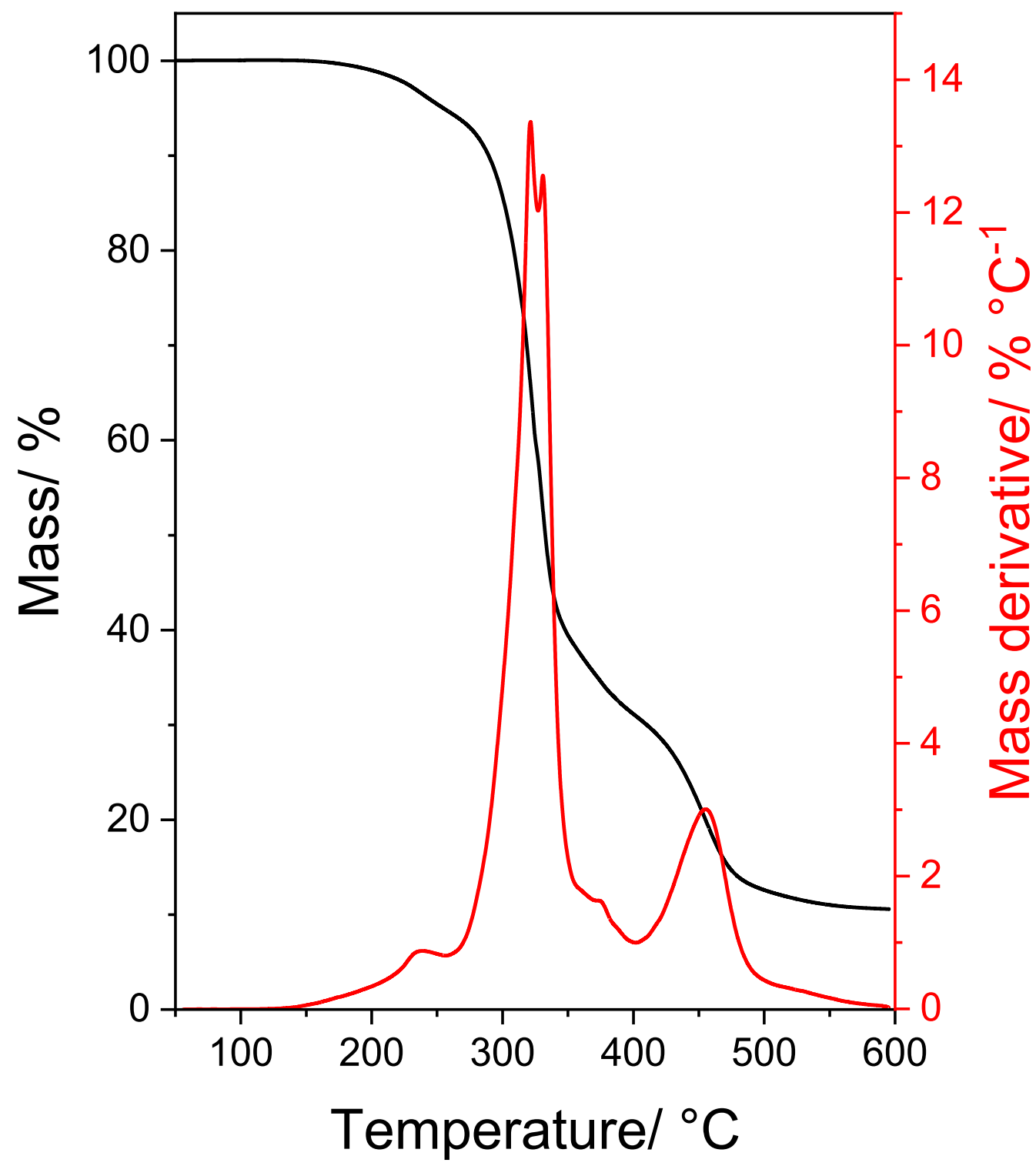


**Figure S29.** TGA and DTG curves for **P(Q-M)**.

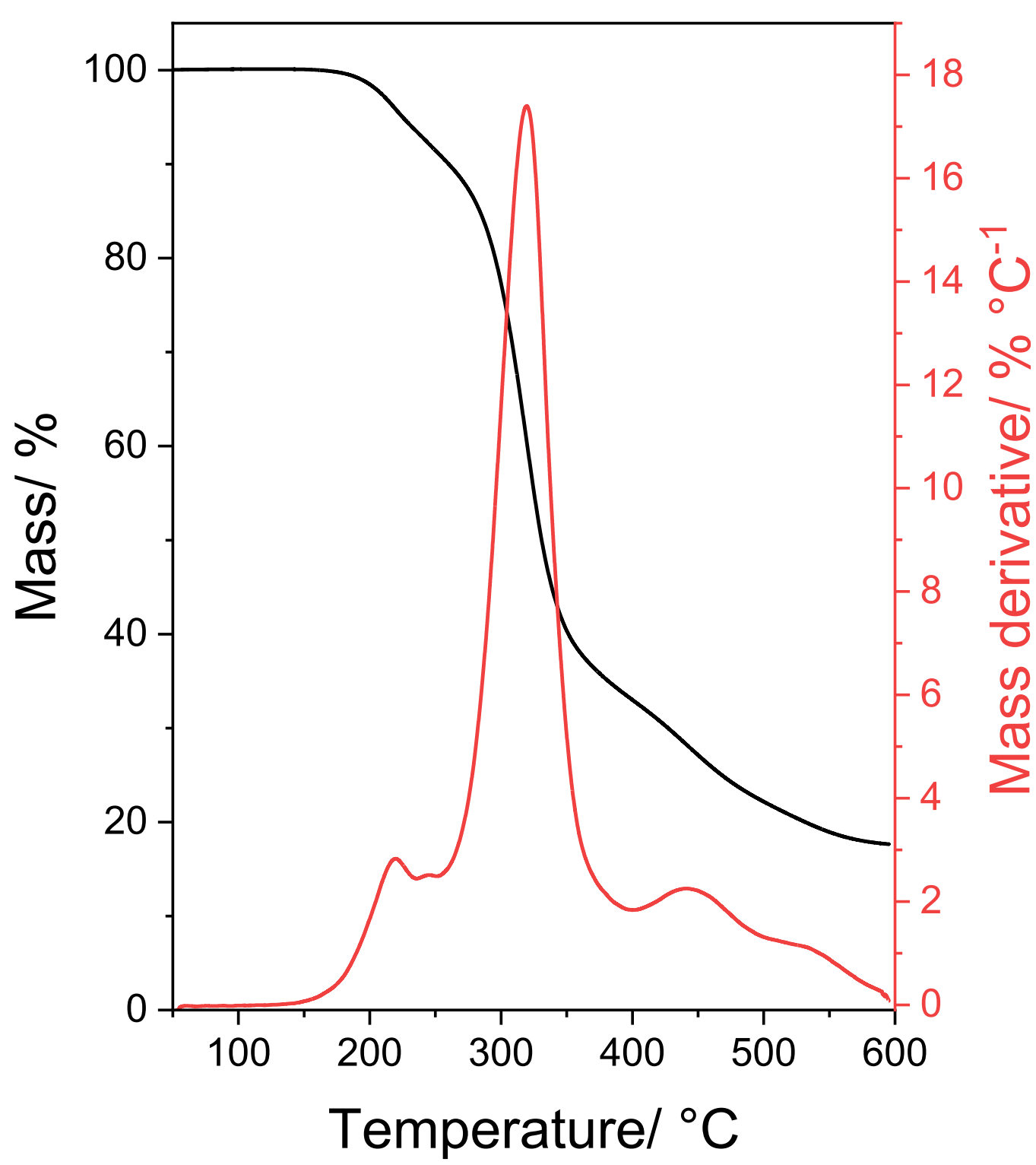


**Figure S30.** TGA and DTG curves for **P($Q_{35}T_{41}F_{19}$-M)**.

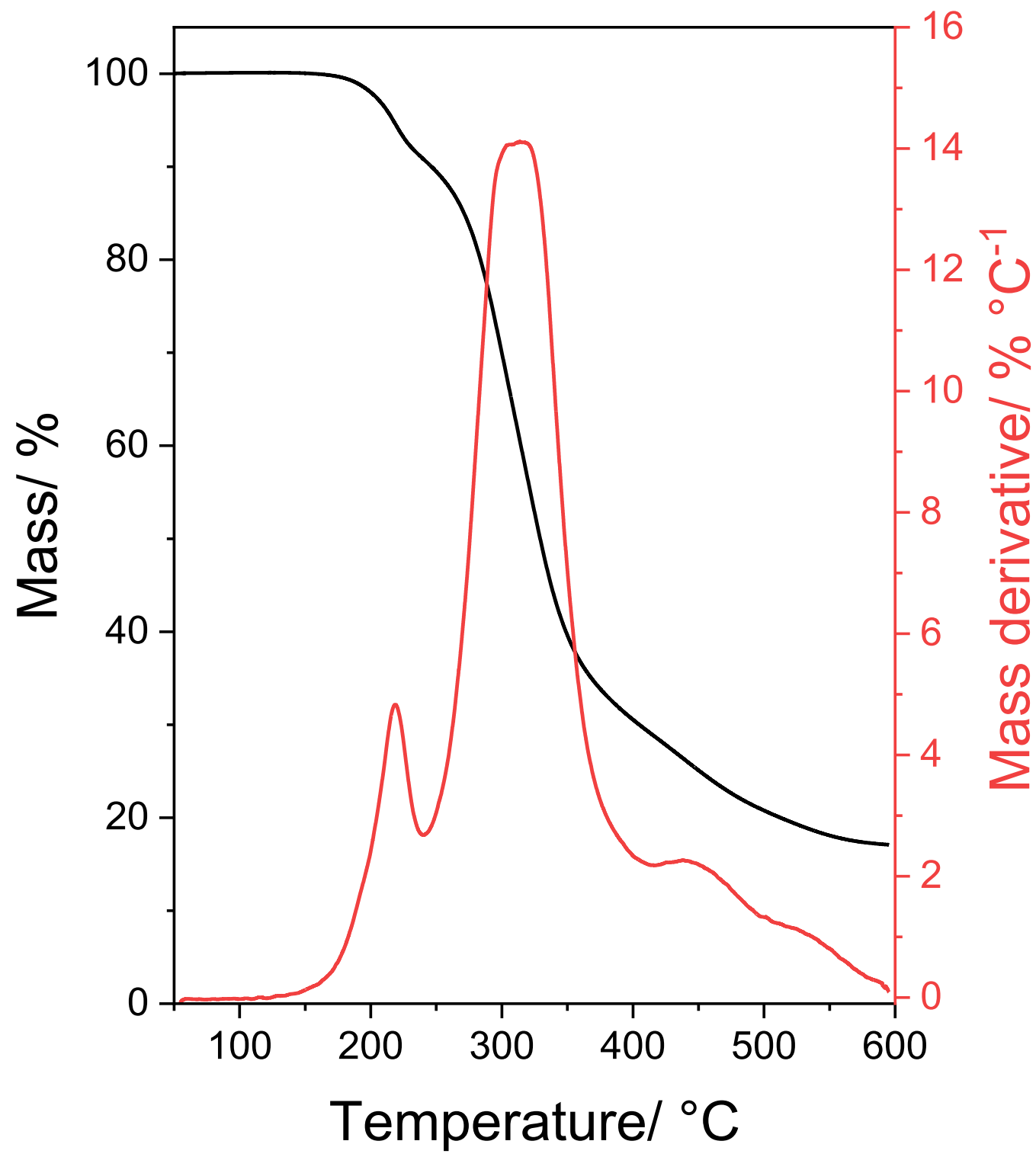


**Figure S31.** TGA and DTG curves for **P($Q_{21}T_{58}F_{21}$-M)**.

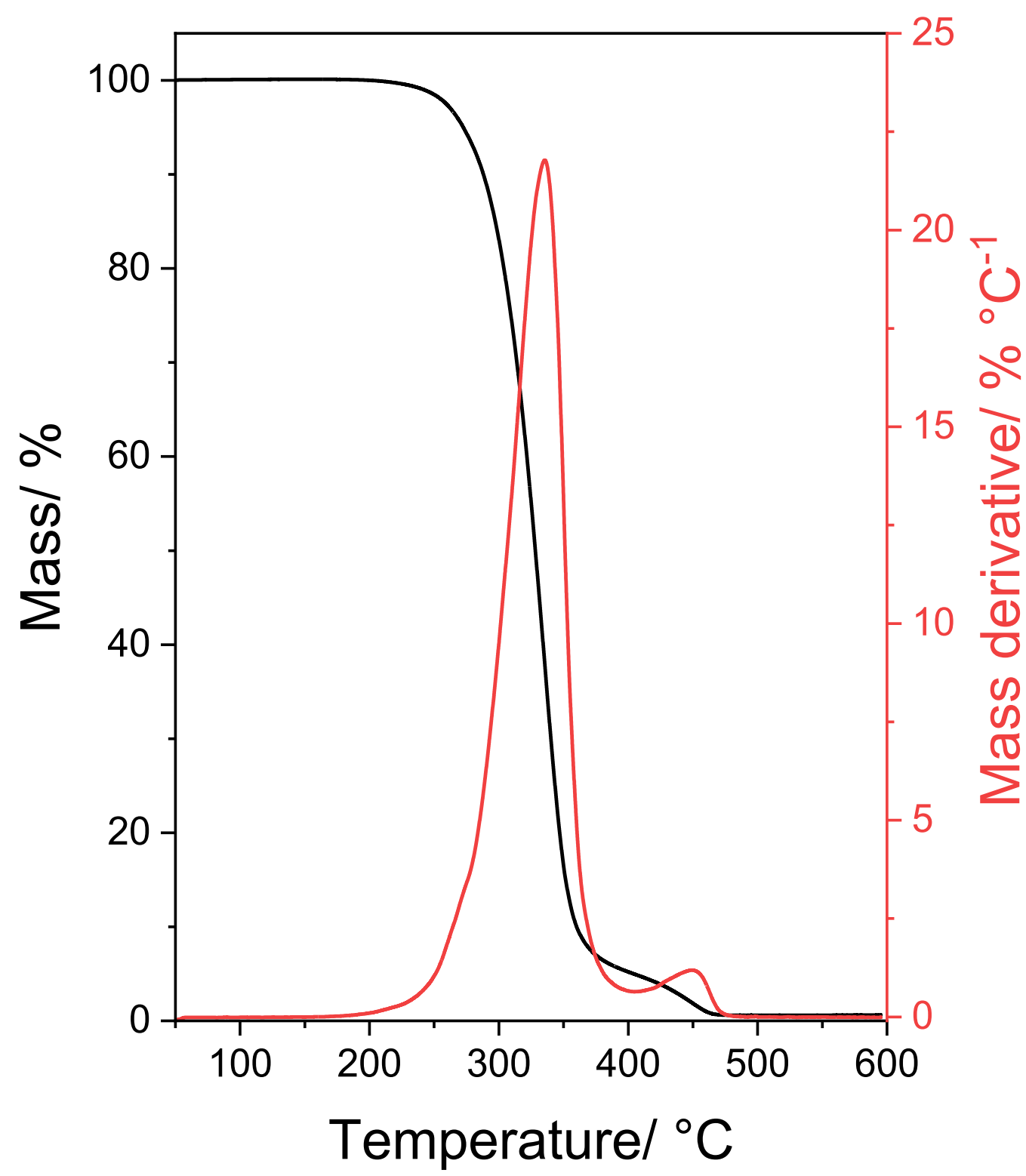


**Figure S32.** TGA and DTG curves for **P(Q-H)**.

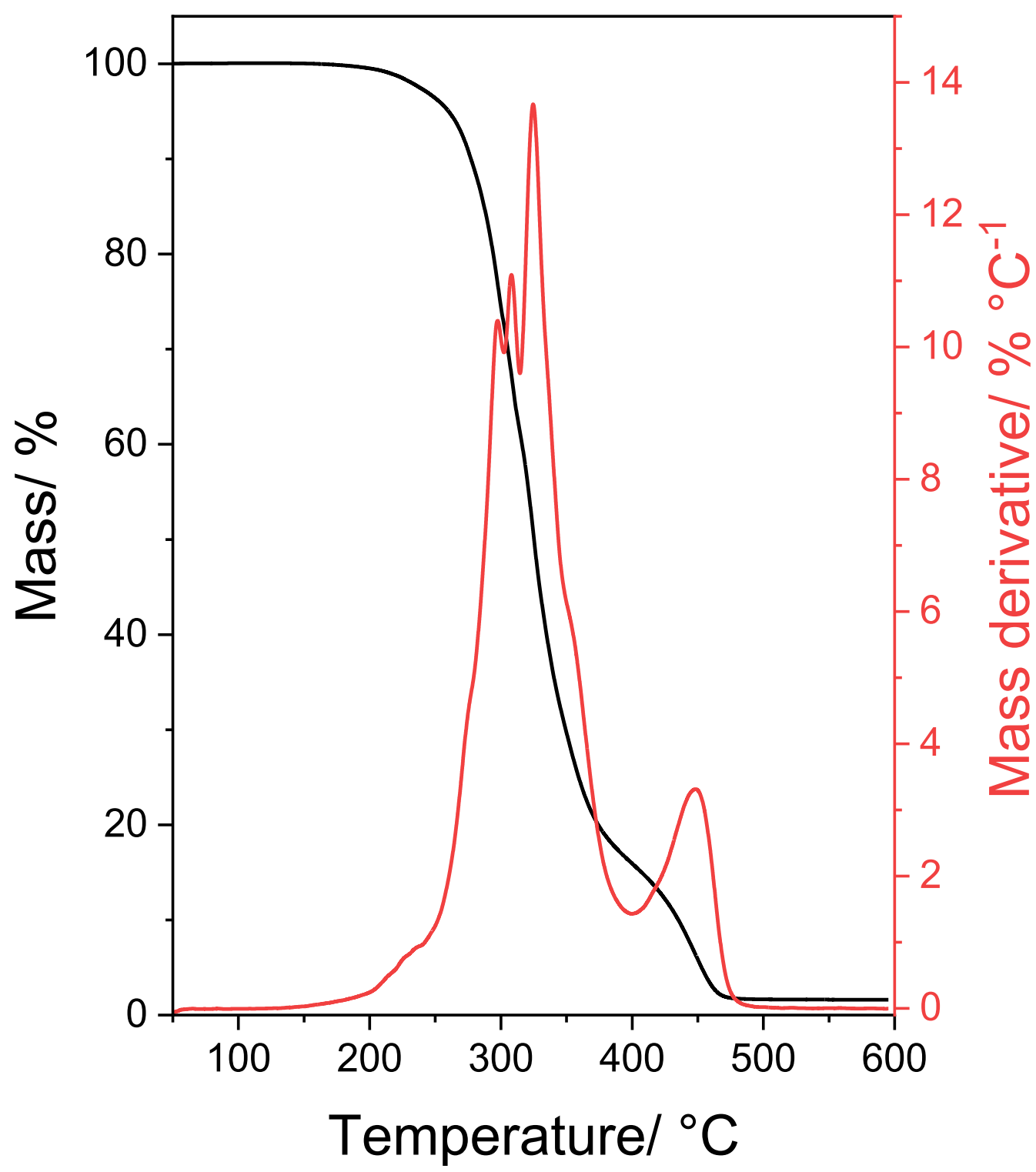


**Figure S33.** TGA and DTG curves for **P($Q_{35}T_{49}F_{16}$-H)**.

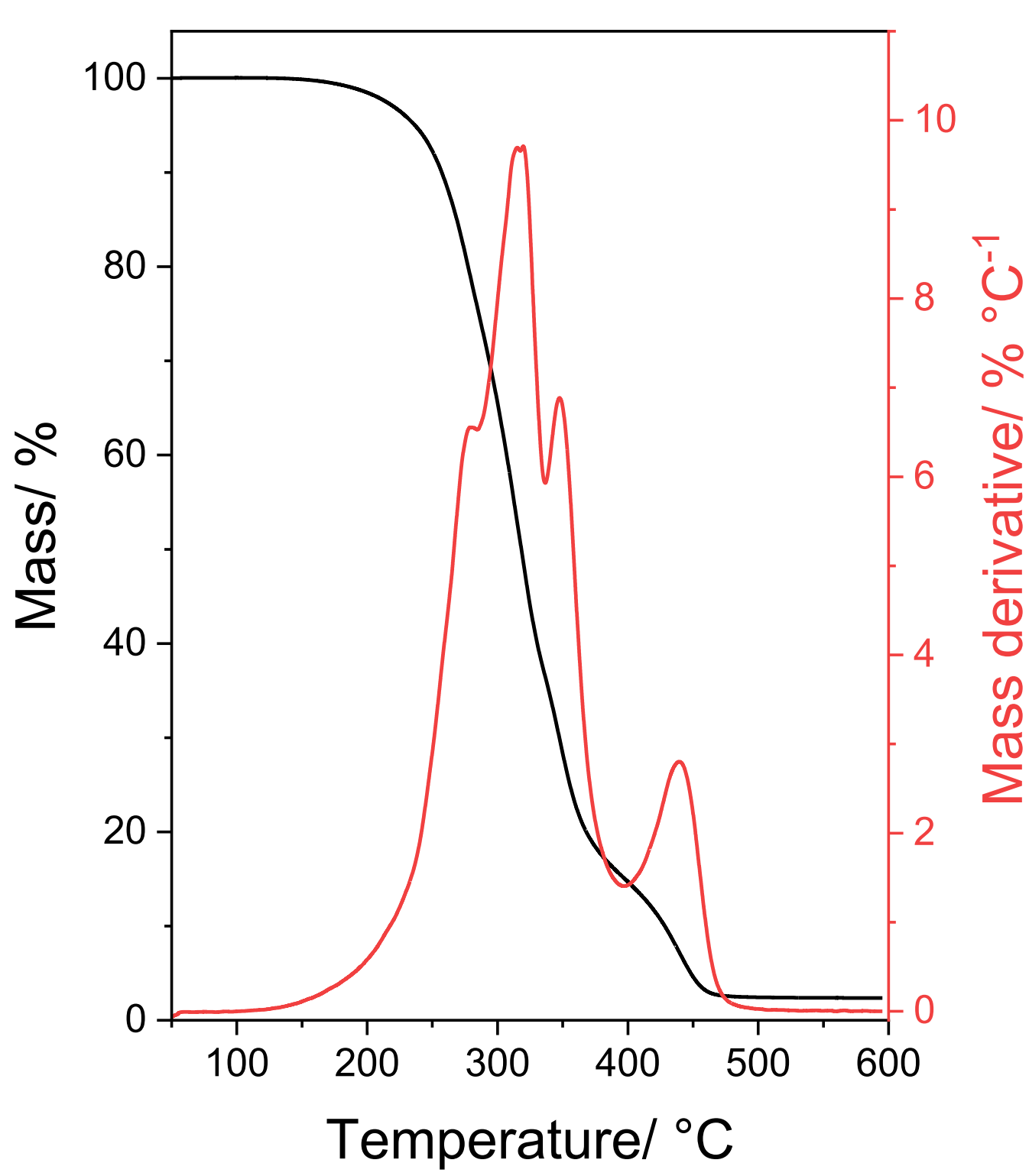


**Figure S34.** TGA and DTG curves for **P($Q_{23}T_{62}F_{15}$-H)**.

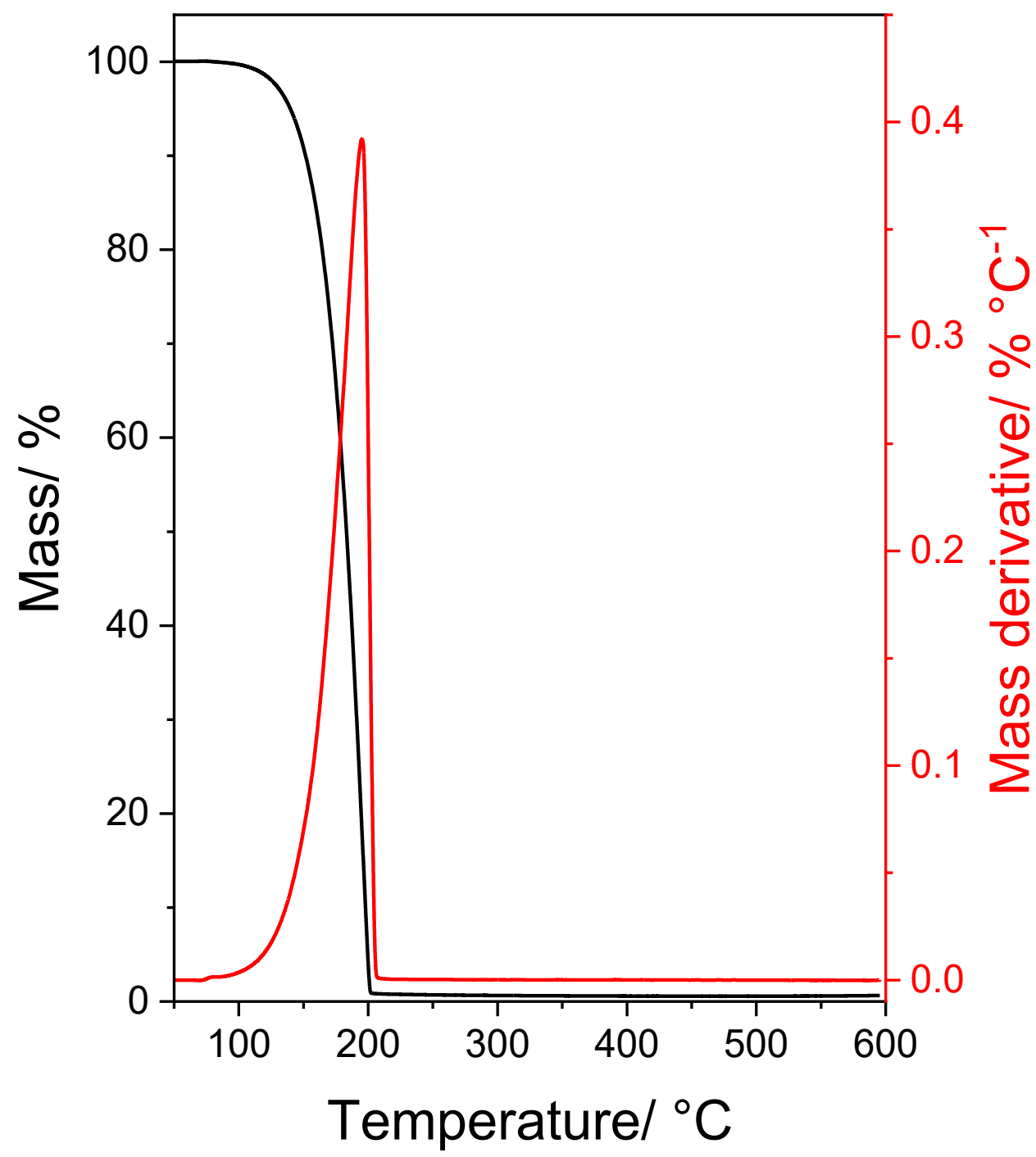


**Figure S35.** TGA and DTG curves for **BHF**.

**Table S1.** Thermal characteristics of PUs. Degradation temperature ($T_d$), temperature at which 5% weight loss is observed ($T_5$) and char yield (CY) were determined by TGA. Glass transition temperature ($T_g$) was measured by DSC from the second heating curve.

| **Polymer** | $T_d$ (°C) | $T_5$ (°C) | $T_g$ (°C) | CY (%) |
|---|---|---|---|---|
| **P(Q-M)** | 239, 321, 331, 455 | 255 | 124 | 11 |
| **P($Q_{35}T_{41}F_{19}$-M)** | 220, 319, 441, | 225 | 70 | 18 |
| **P($Q_{21}T_{58}F_{21}$-M)** | 219, 316, 442 | 217 | 70 | 17 |
| **P(Q-H)** | 335, 449 | 272 | 90 | 1 |
| **P($Q_{35}T_{49}F_{16}$-H)** | 297, 308, 324, 448 | 260 | 78 | 2 |
| **P($Q_{23}T_{62}F_{15}$-H)** | 279, 315, 348, 440 | 236 | 31 | 2 |

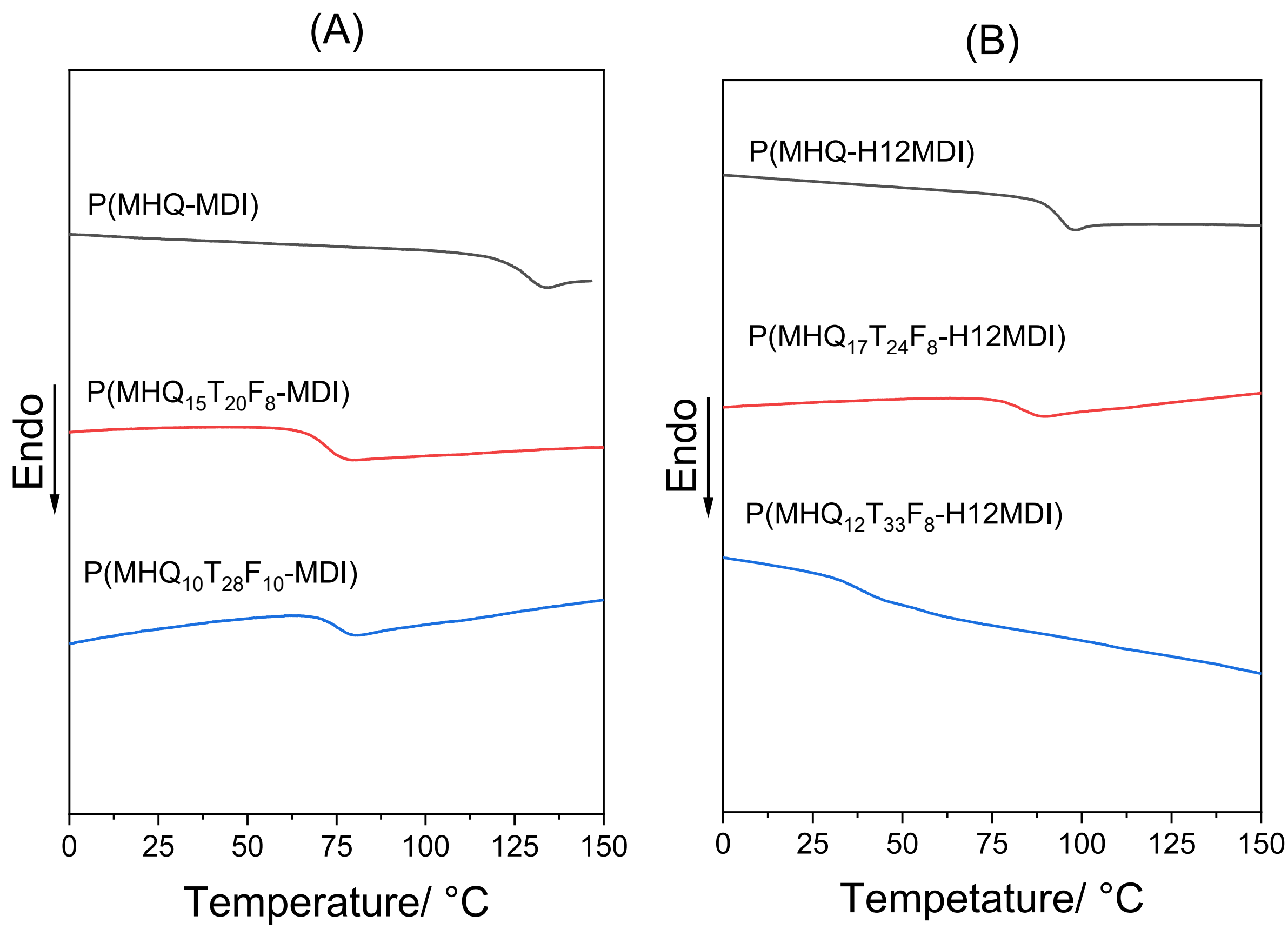


**Figure S36.** DSC curves (2$^{nd}$ heating) for **Q-based PU**.

## 7. Properties of Q-based fibers

**(A)**

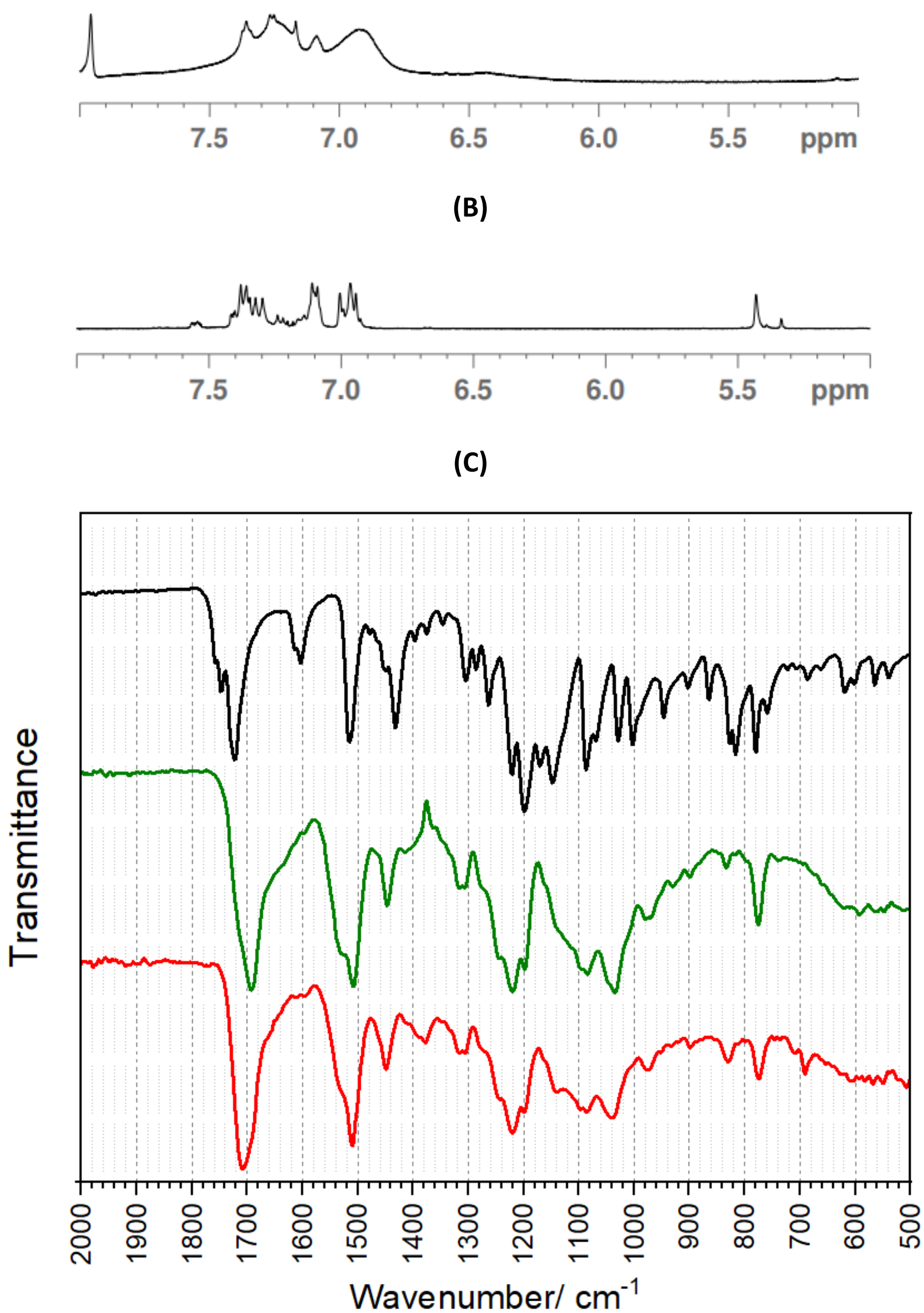


**Figure S37.** $^{1}$H NMR spectra of **(A) P($Q_{21}T_{58}F_{21}$-M)** and **(B) P($Q_{35}T_{49}F_{16}$-H)** fibres (draw ratio = 1.0) previously swollen in DMSO-$d_6$ for 72 h. **(C)** FTIR spectra of monomer **Q** (black), and **P($Q_{35}T_{49}F_{16}$-H)** fibre before (green) and after crosslinking (red).

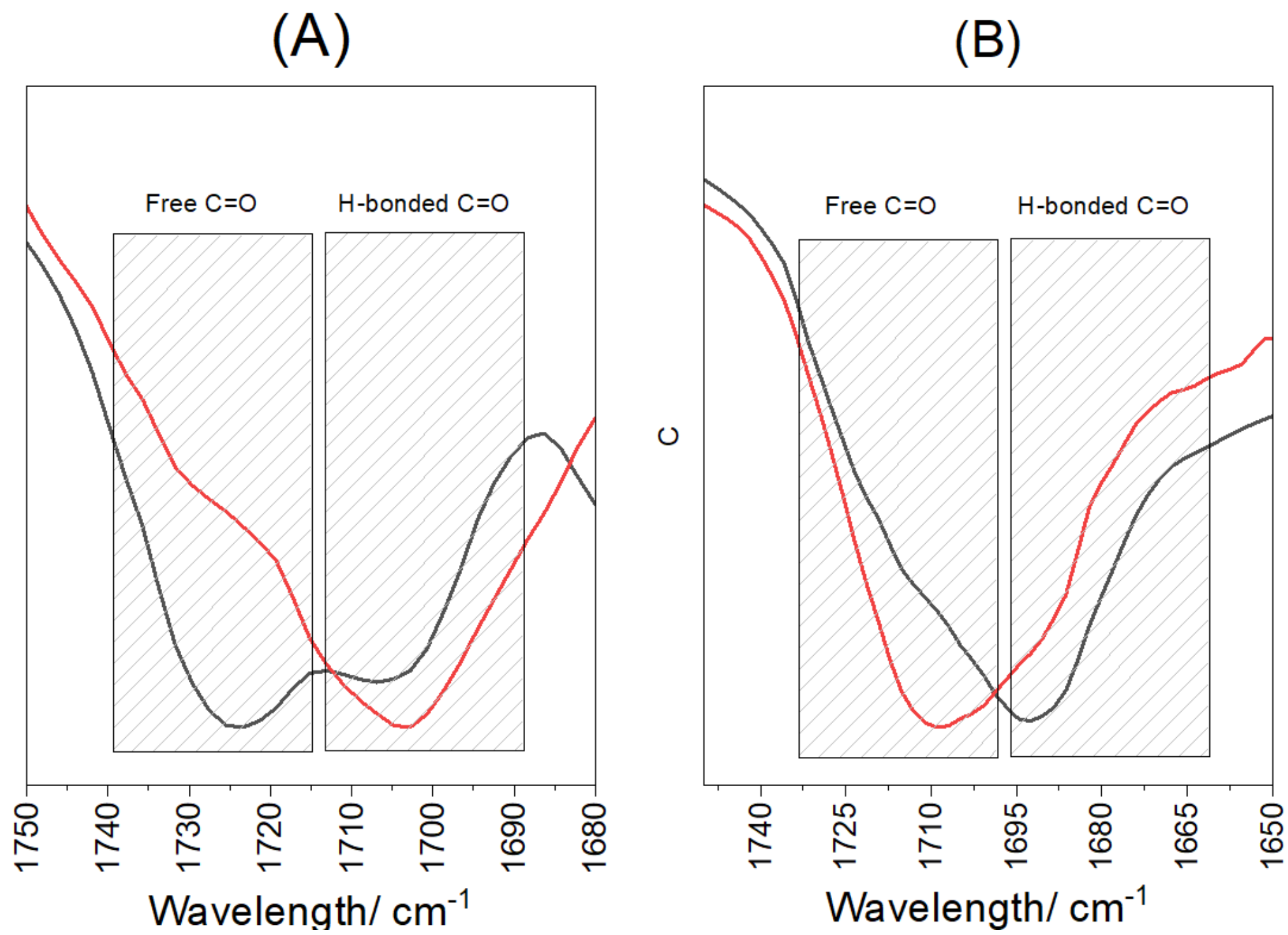


**Figure S38.** FTIR spectra of **(A) P($Q_{21}T_{58}F_{21}$-M)** and **(B) P($Q_{35}T_{49}F_{16}$-H)** fibres (draw ratio = 1.0) before (black) and after crosslinking (red).

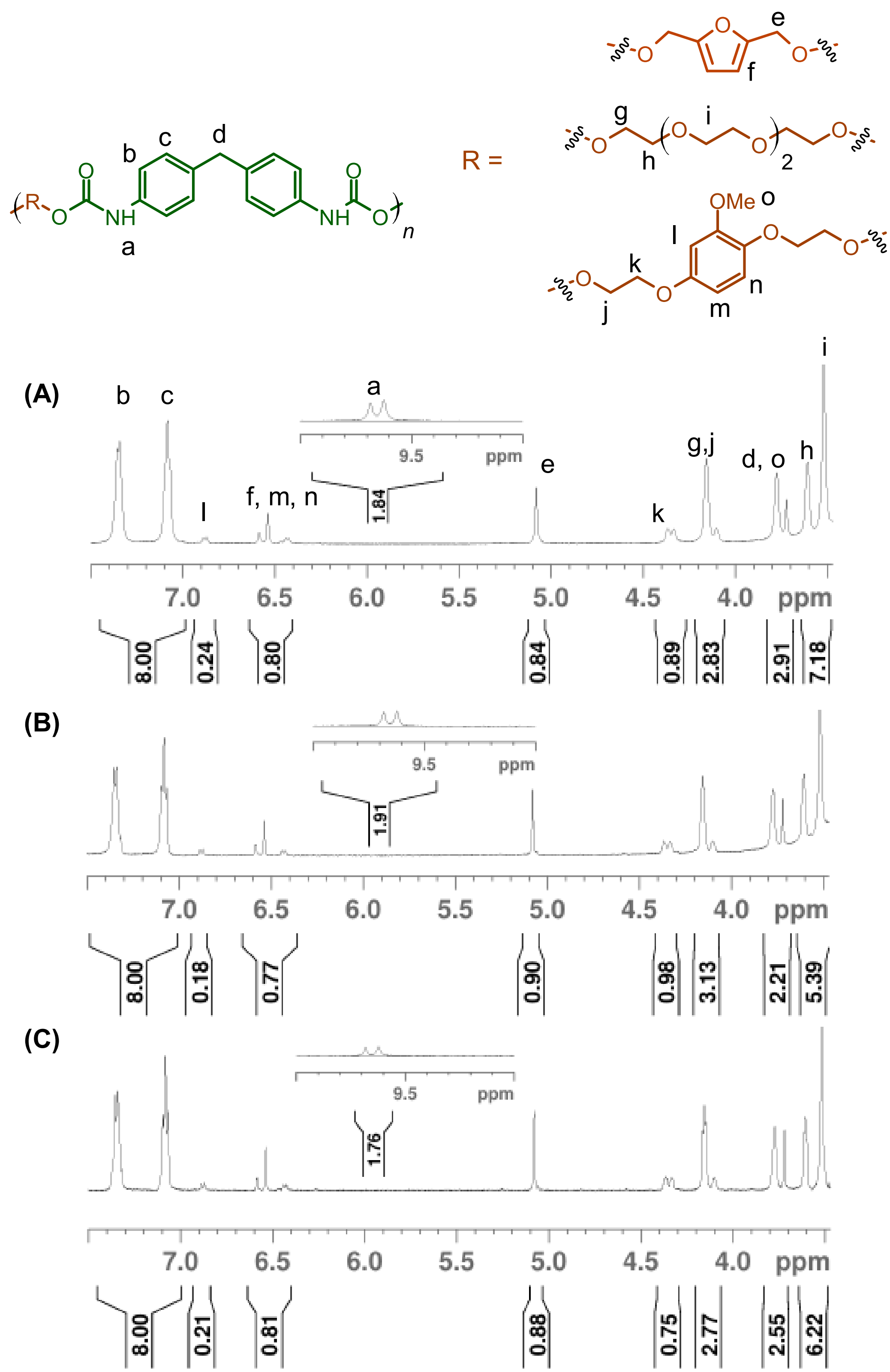

**Figure S39**. $^{1}$H-NMR spectra of **P(Q$_{21}$T$_{58}$F$_{21}$-M)** obtained at draw ratio of **(A)** 1.0, **(B)** 1.2, and **(C)** 1.4.

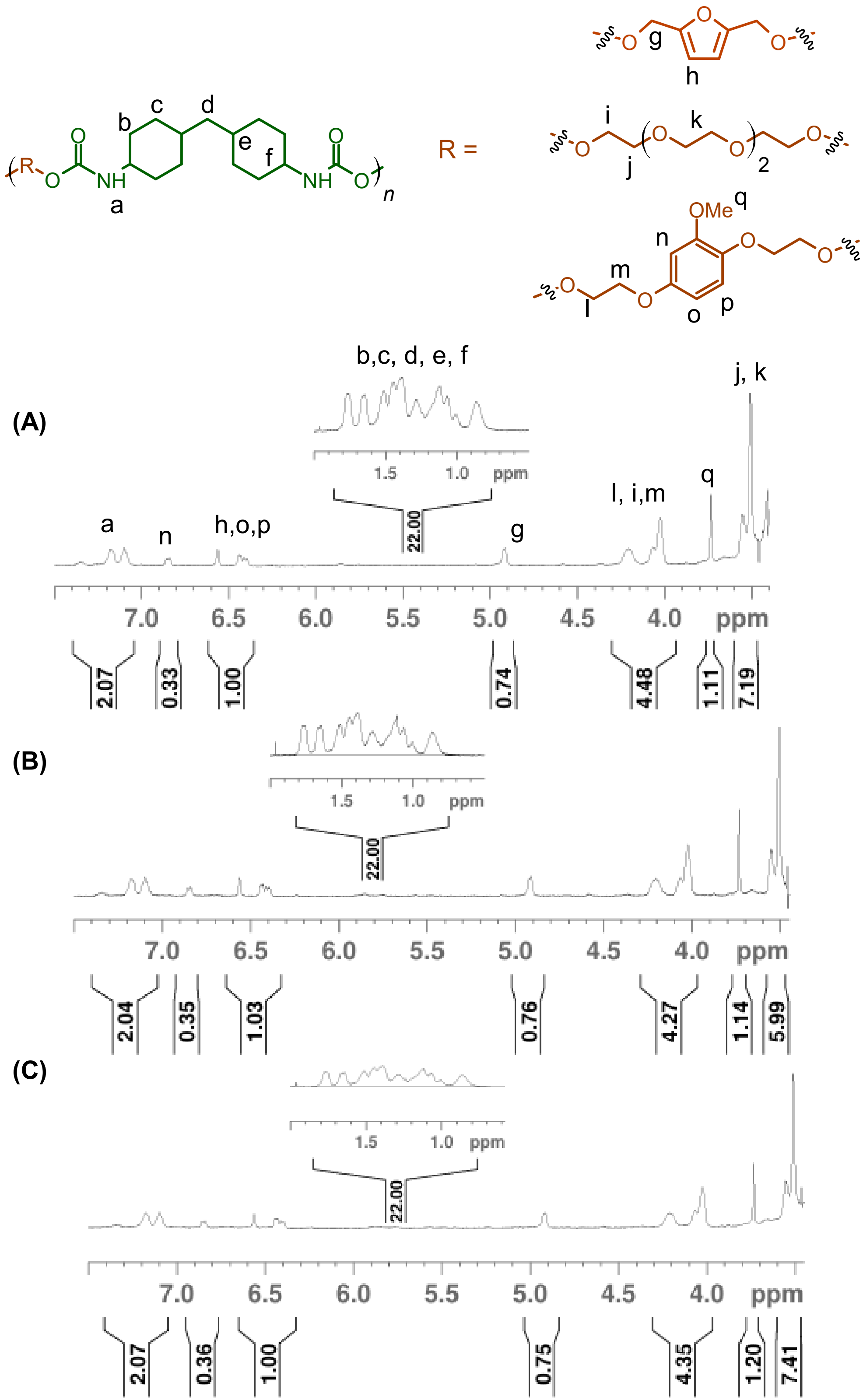


**Figure S40**. $^{1}$H-NMR spectra of **$P(Q_{35}T_{49}F_{16}\text{-}H)$** obtained at draw ratio of **(A)** 1.0, **(B)** 1.2, and **(C)** 1.4.

**(A)**

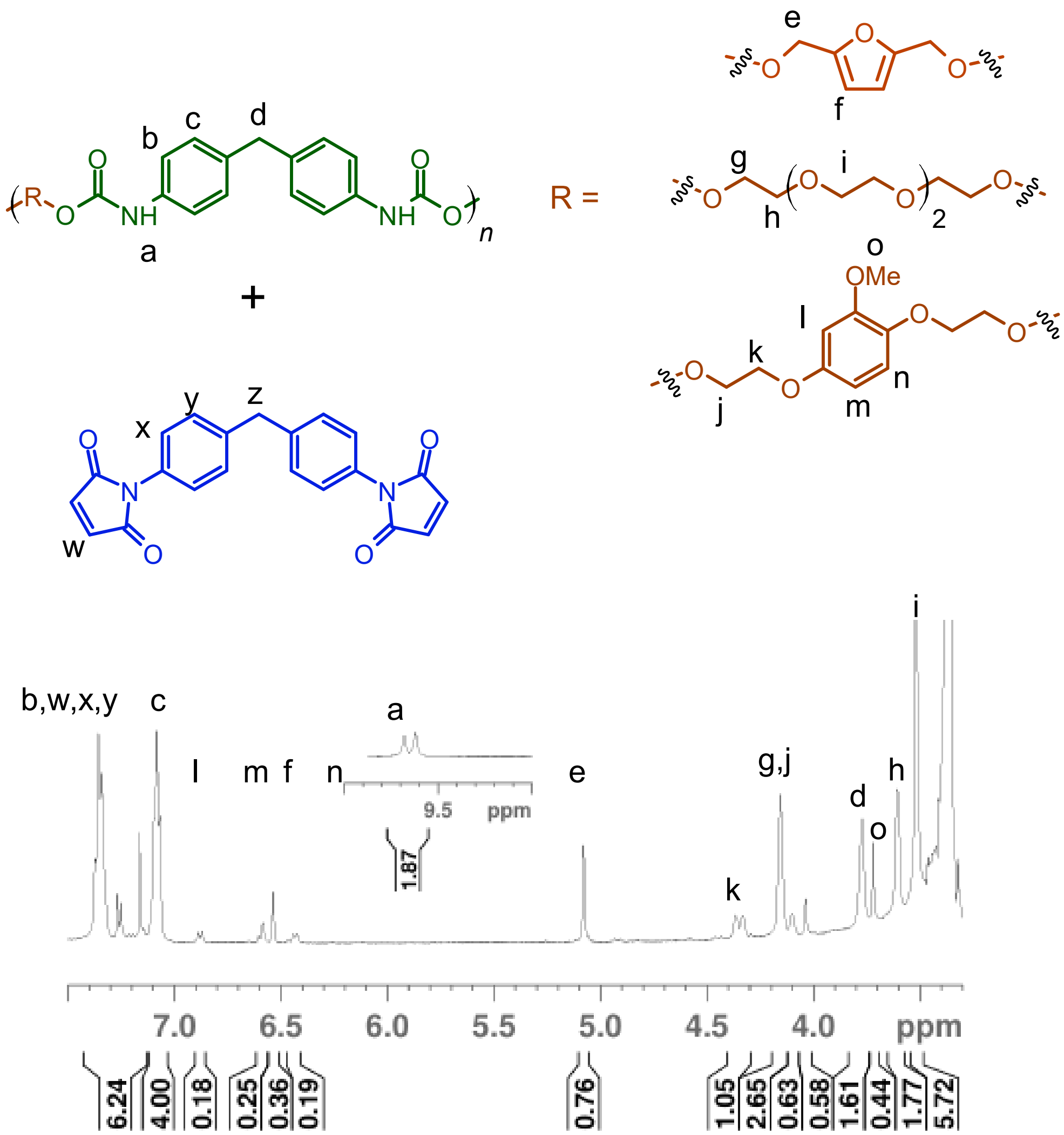


**(B)**

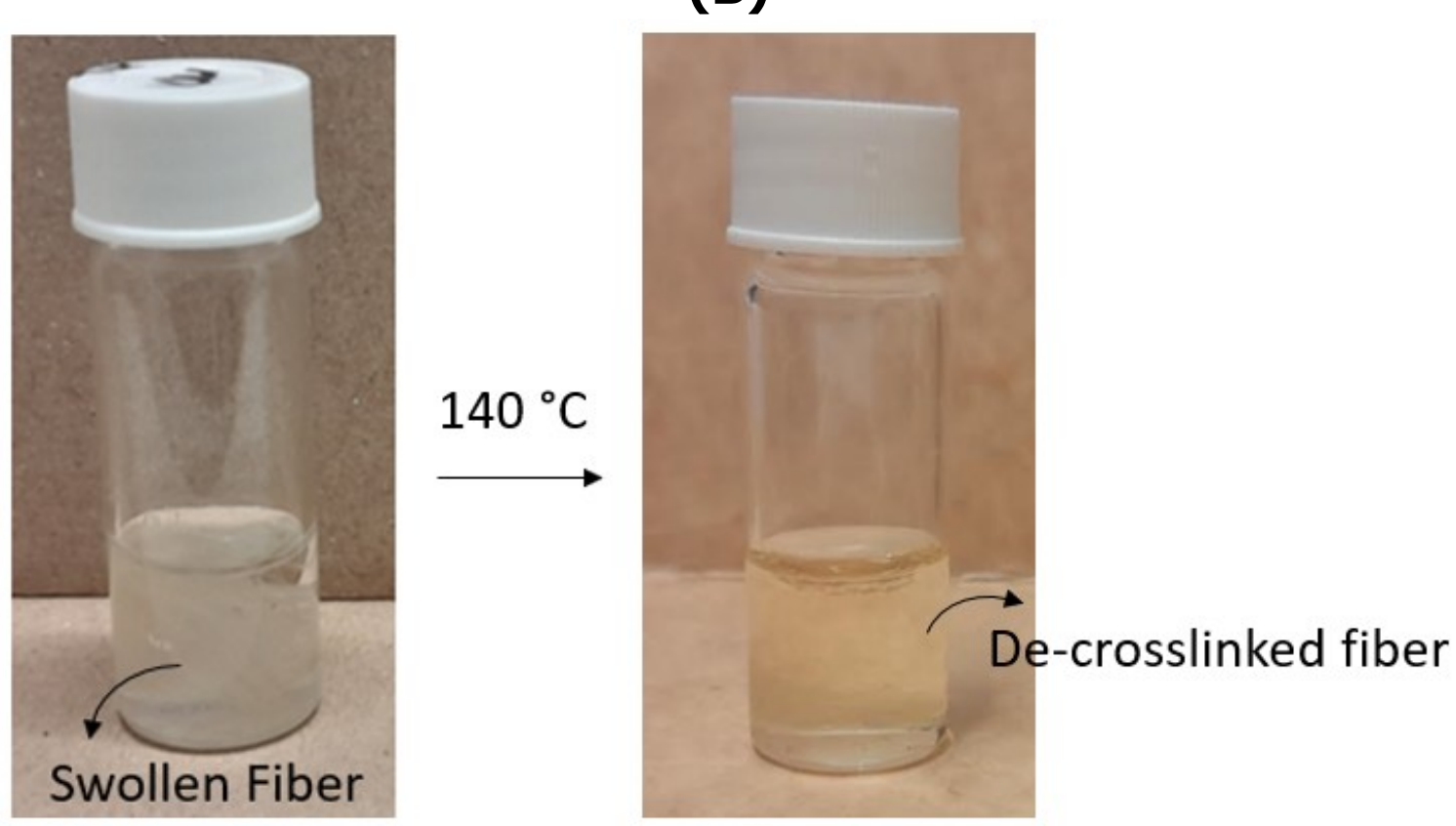


**Figure S41. (A)** $^{1}$H-NMR spectra of **P($Q_{21}T_{58}F_{21}$-M)** obtained at draw ratio of 1.0 after de-crosslinking at 140 °C. **(B) P($Q_{21}T_{58}F_{21}$-M)** de-crosslinking.

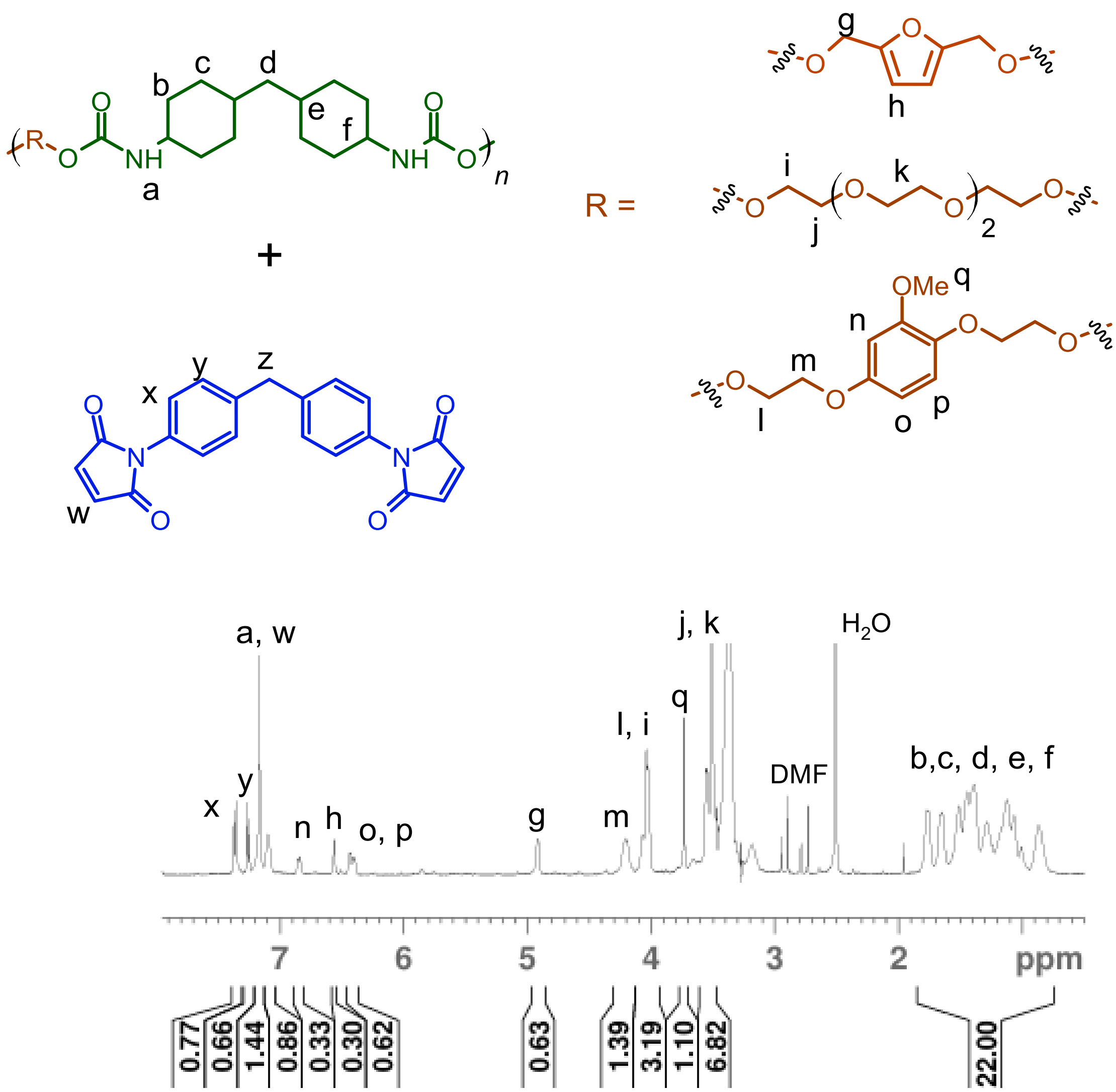


**Figure S42.** $^{1}$H-NMR spectra of **P(Q$_{35}$T$_{49}$F$_{16}$-H)** obtained at draw ratio of 1.0 after de-crosslinking at 140 °C.

**Thermal analysis**

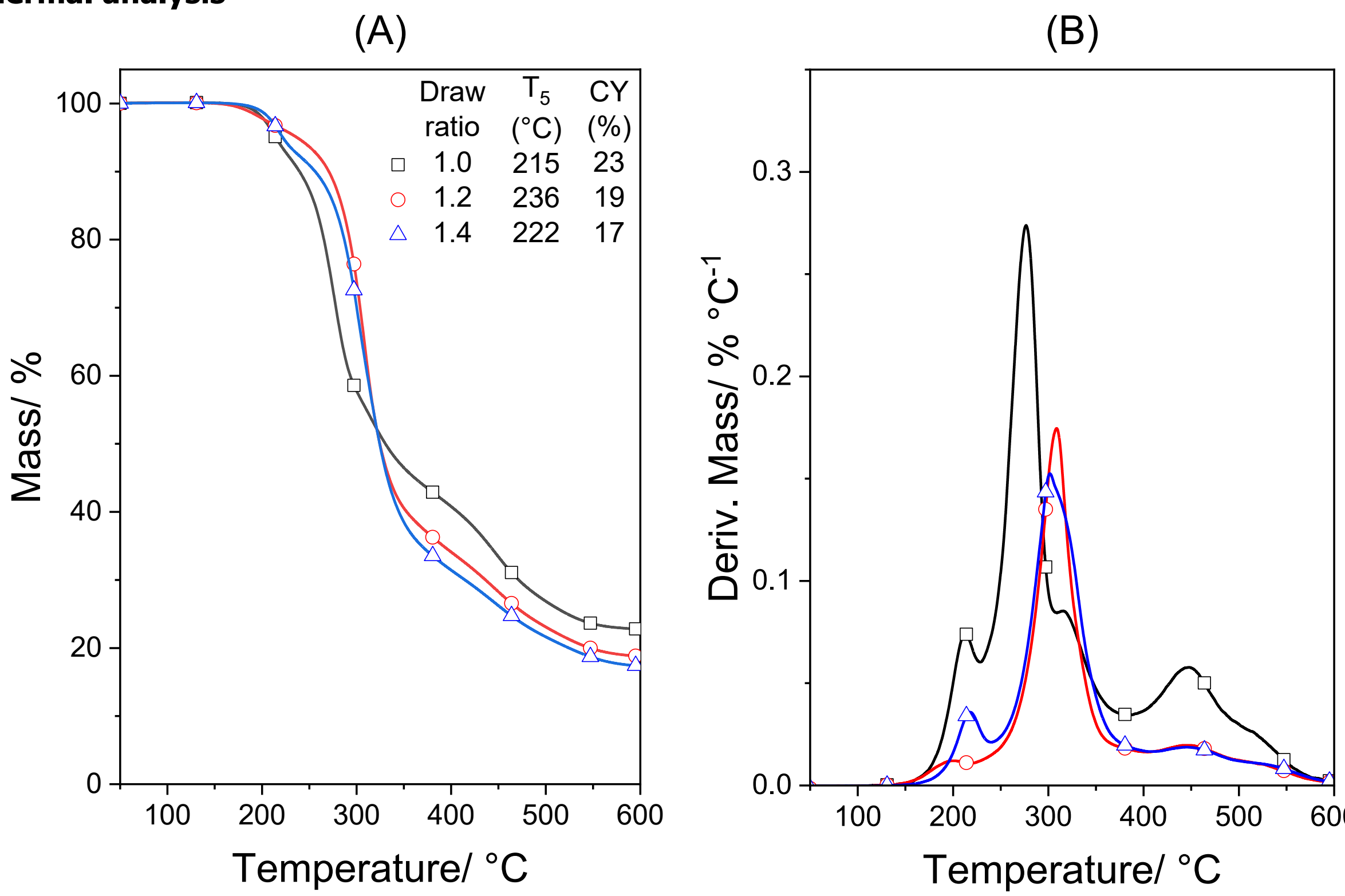


**Figure S43. (A)** TGA and **(B)** DTG curves for non-crosslinked **P($Q_{21}T_{58}F_{21}$-M)** fibres.

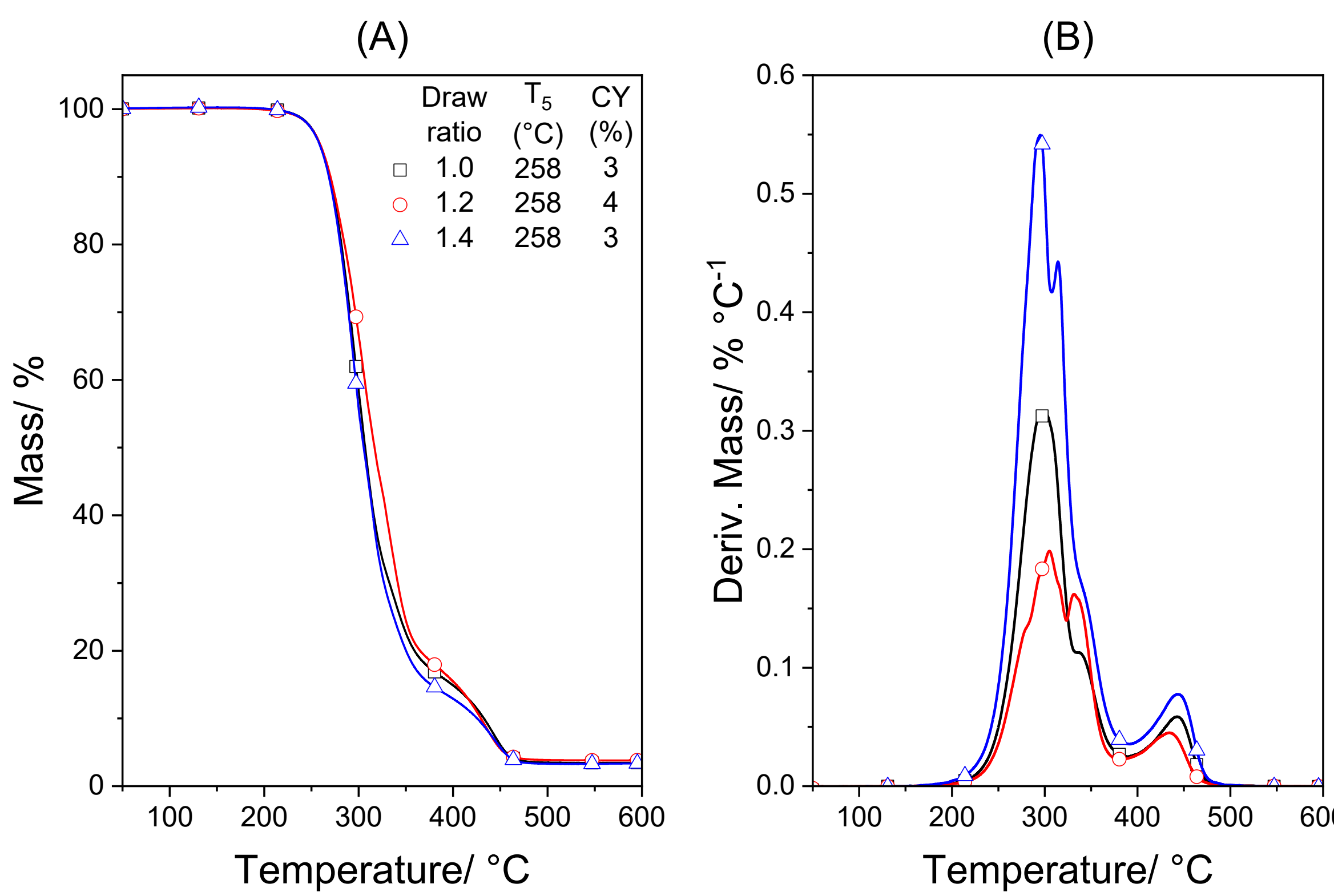


**Figure S44. (A)** TGA and **(B)** DTG curves for non-crosslinked **P($Q_{35}T_{49}F_{16}$-H)** fibres.

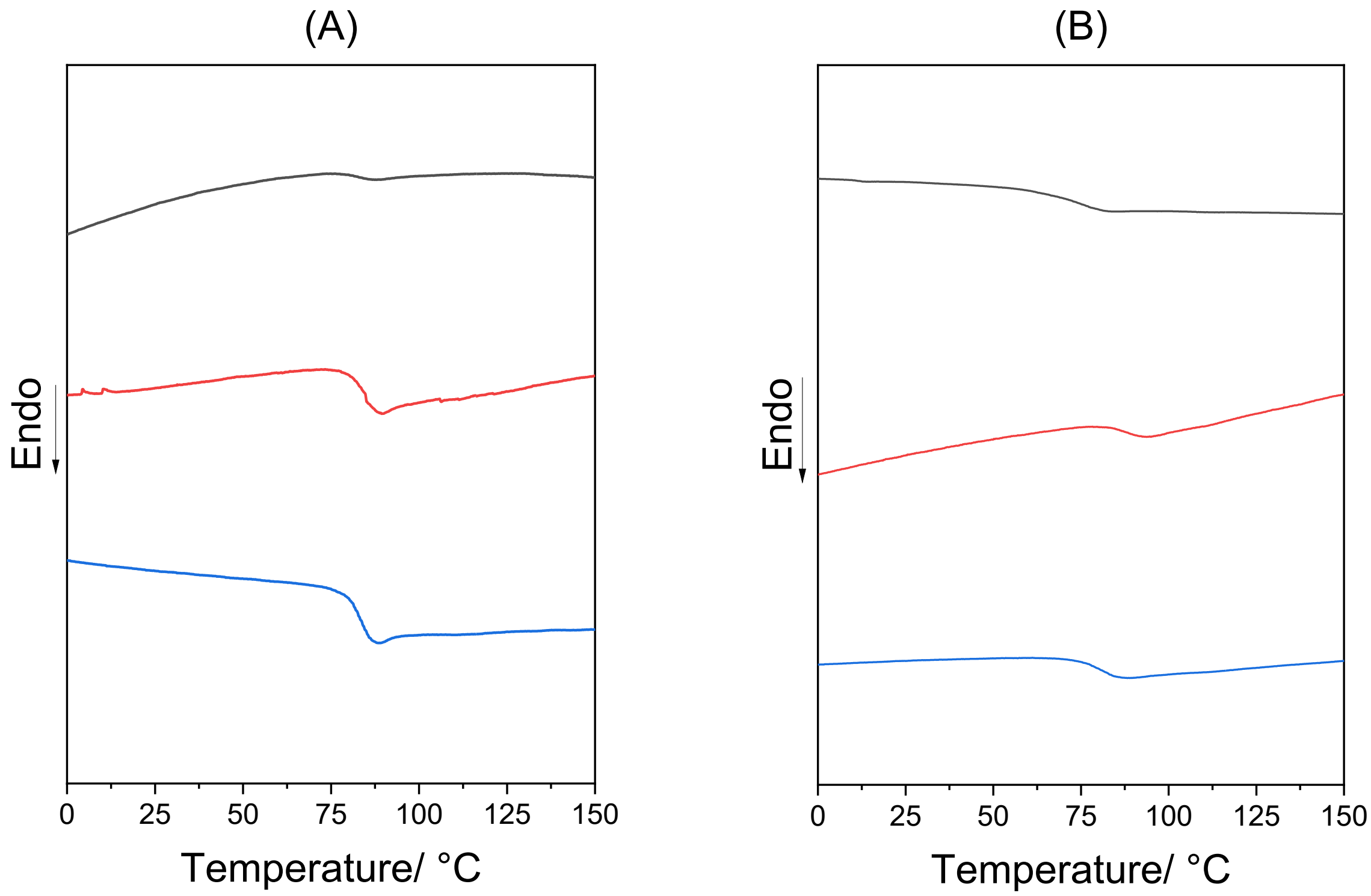


**Figure S45**. DSC curves (2nd heating) for non-crosslinked (**A) P($Q_{35}T_{49}F_{16}$-H)** and **(B) P($Q_{21}T_{58}F_{21}$-M)** fibers obtained at different draw ratios.

**Table S2**. Thermal and mechanical data for non-crosslinked and crosslinked fibres obtained at different draw ratio. Elong. stands for elongation at break. Ten. stands for tensile strength. YM stands for Young's modulus. The error correspond to the standard deviation.

| Fibre | | Draw ratio | $T_g$ (°C) | Elong. (%) | Ten. (cN tex$^{-1}$) | YM (cN tex$^{-1}$) |
|---|---|---|---|---|---|---|
| **P($Q_{21}T_{58}F_{21}$-M)** | Non-crosslinked | 1.0 | 77 | 10.9 ± 4.8 | 7.6 ± 2.4 | 258 ± 64 |
| | Non-crosslinked | 1.2 | 81 | 4.7 ± 1.2 | 2.9 ± 0.7 | 139 ± 23 |
| | Non-crosslinked | 1.4 | 80 | 3.3 ± 0.9 | 3.6 ± 0.4 | 147 ± 52 |
| | Crosslinked | 1.0 | 87 | 10.0 ± 0.6 | 1.9 ± 0.4 | 106 ± 5 |
| | Crosslinked | 1.2 | - [a] | 9.4 ± 3.4 | 3.9 ± 0.4 | 145 ± 26 |
| | Crosslinked | 1.4 | - [a] | 9.8 ± 4.9 | 3.0 ± 0.4 | 111 ± 22 |
| **P($Q_{35}T_{49}F_{16}$-H)** | Non-crosslinked | 1.0 | 77 | 32.1 ± 2.4 | 2.4 ± 0.1 | 110 ± 8 |
| | Non-crosslinked | 1.2 | 80 | 2.3 ± 0.6 | 1.8 ± 0.3 | 98 ± 2 |
| | Non-crosslinked | 1.4 | 75 | 5.2 ± 1.3 | 2.2 ± 0.2 | 87 ± 8 |
| | Crosslinked | 1.0 | 84 | 25.2 ± 6.1 | 2.5 ± 0.5 | 109 ± 12 |
| | Crosslinked | 1.2 | - [a] | 3.3 ± 0.6 | 2.9 ± 0.5 | 137 ± 18 |
| | Crosslinked | 1.4 | - [a] | 4.3 ± 1.5 | 5.4 ± 0.6 | 184 ± 7 |

[a] Not measured.

**P(Q$_{35}$T$_{49}$F$_{16}$-H)**

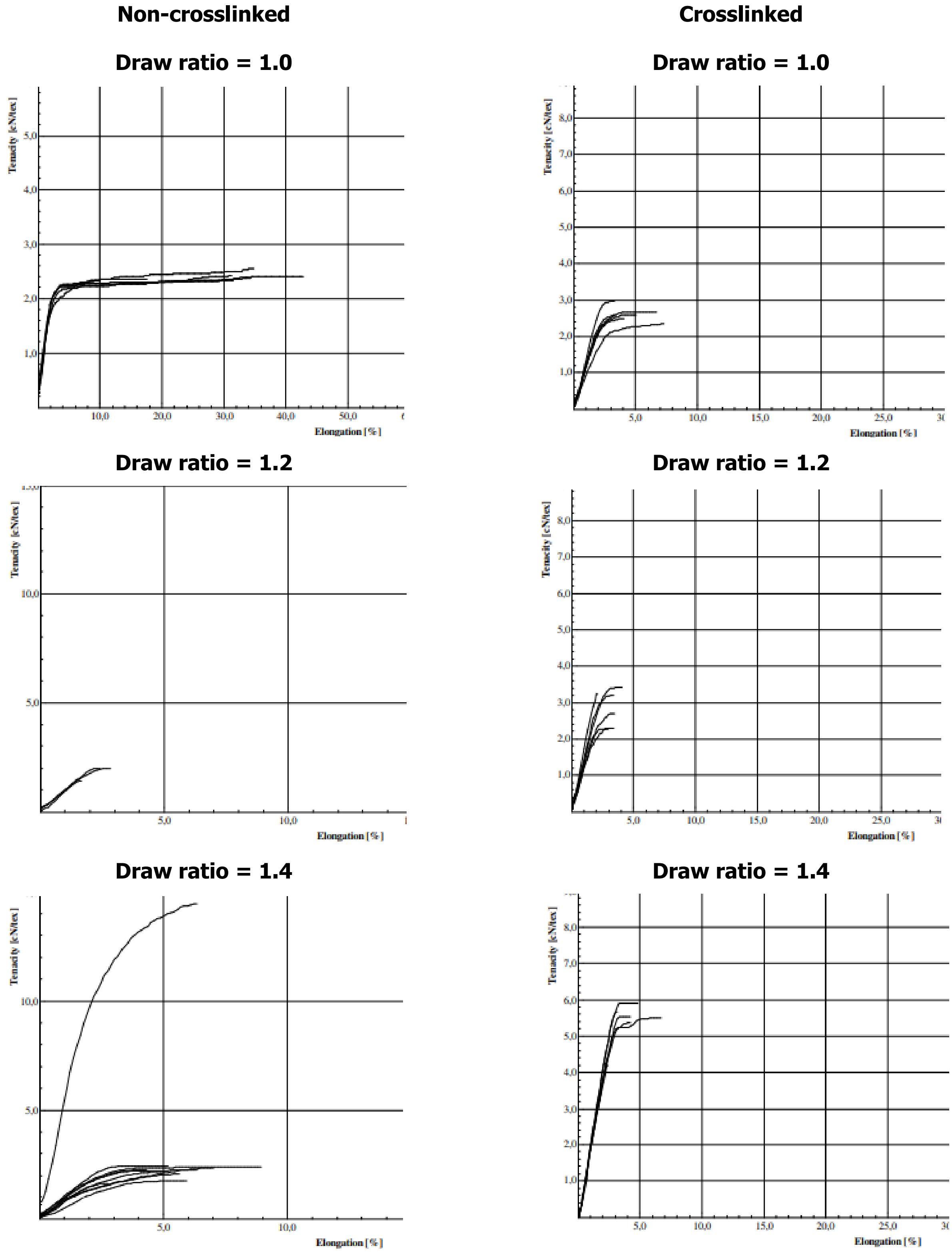


**Figure S46.** Stress-strain curves for **P(Q$_{21}$T$_{58}$F$_{21}$-M)** fibres.

**P(Q$_{21}$T$_{58}$F$_{21}$-M)**

**Non-crosslinked** | **Crosslinked**

**Draw ratio = 1.0**

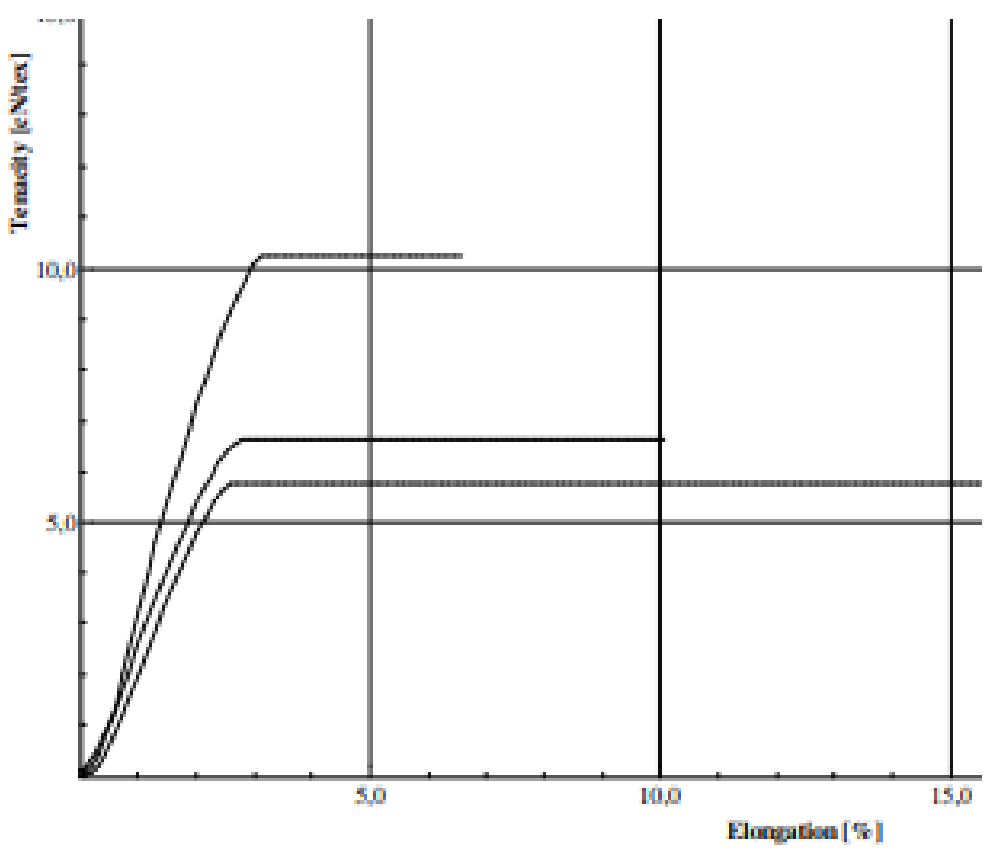


**Draw ratio = 1.0**

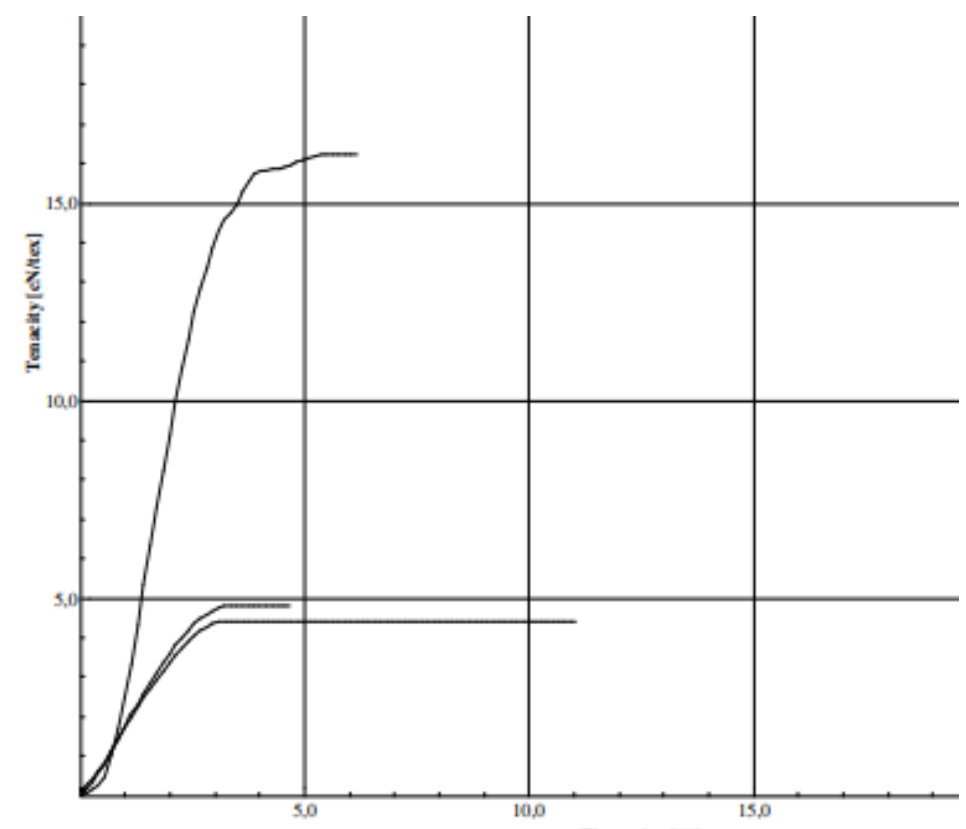


**Draw ratio = 1.2**

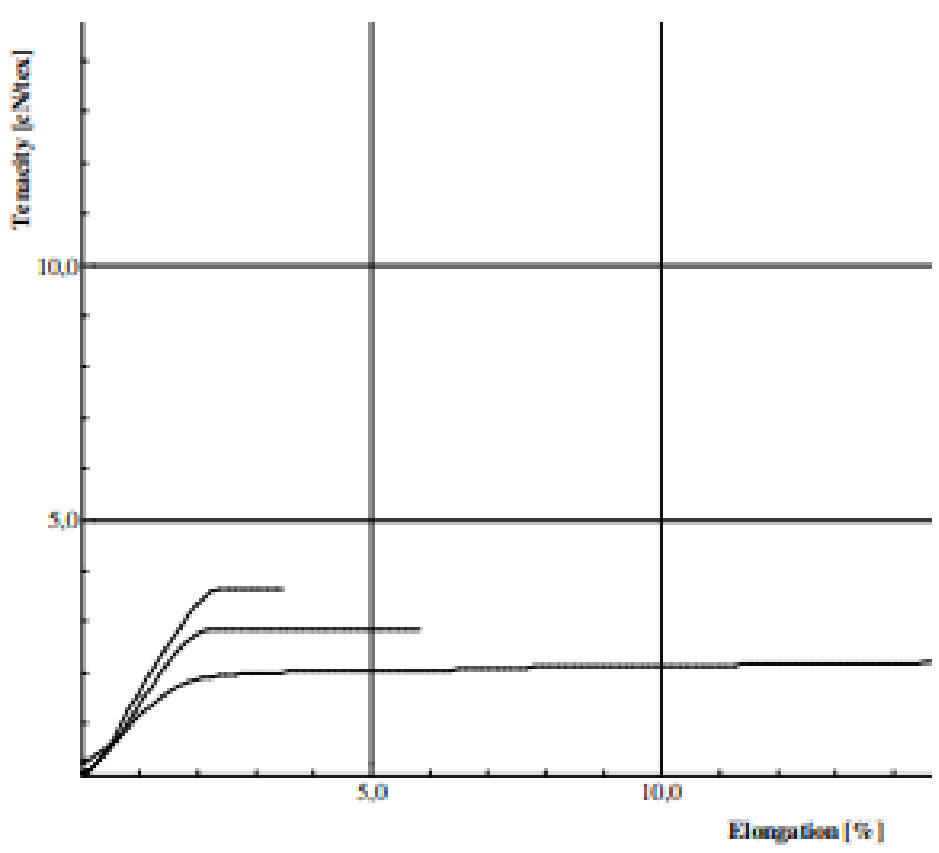


**Draw ratio = 1.2**

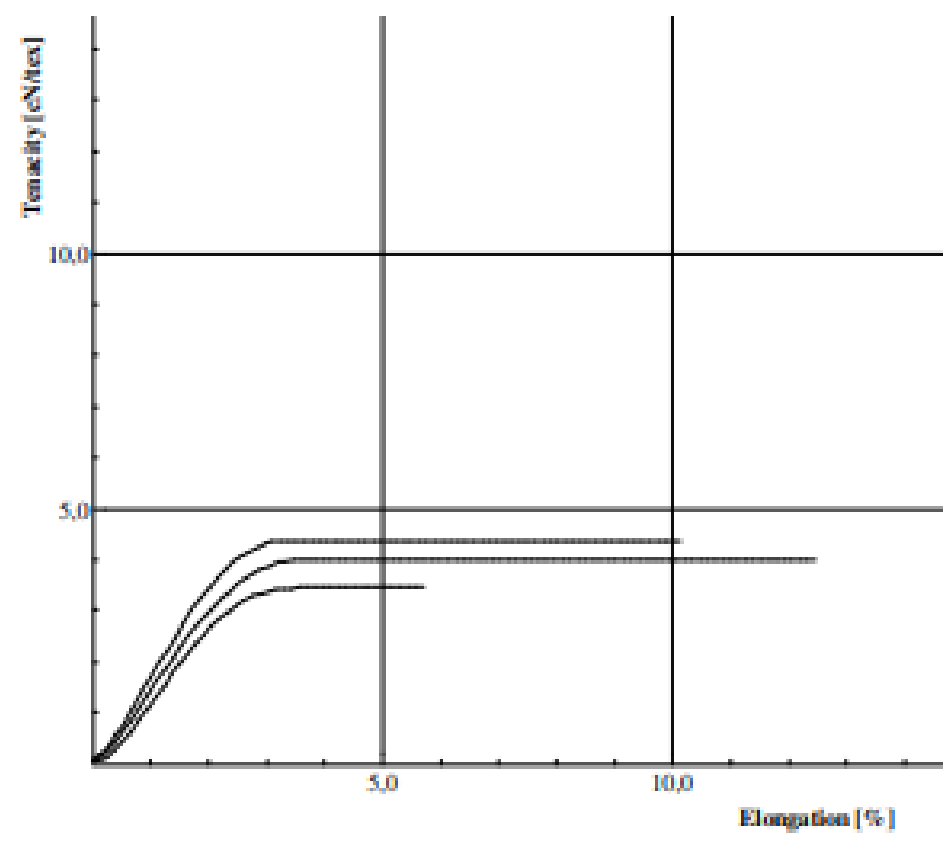


**Draw ratio = 1.4**

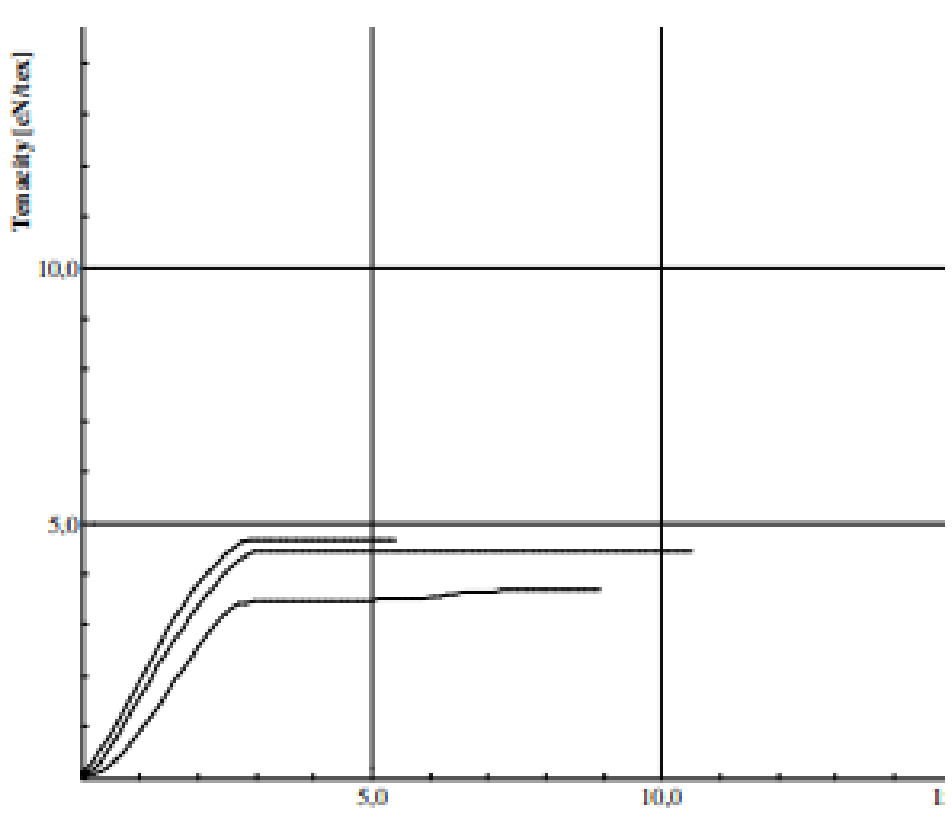


**Draw ratio = 1.4**

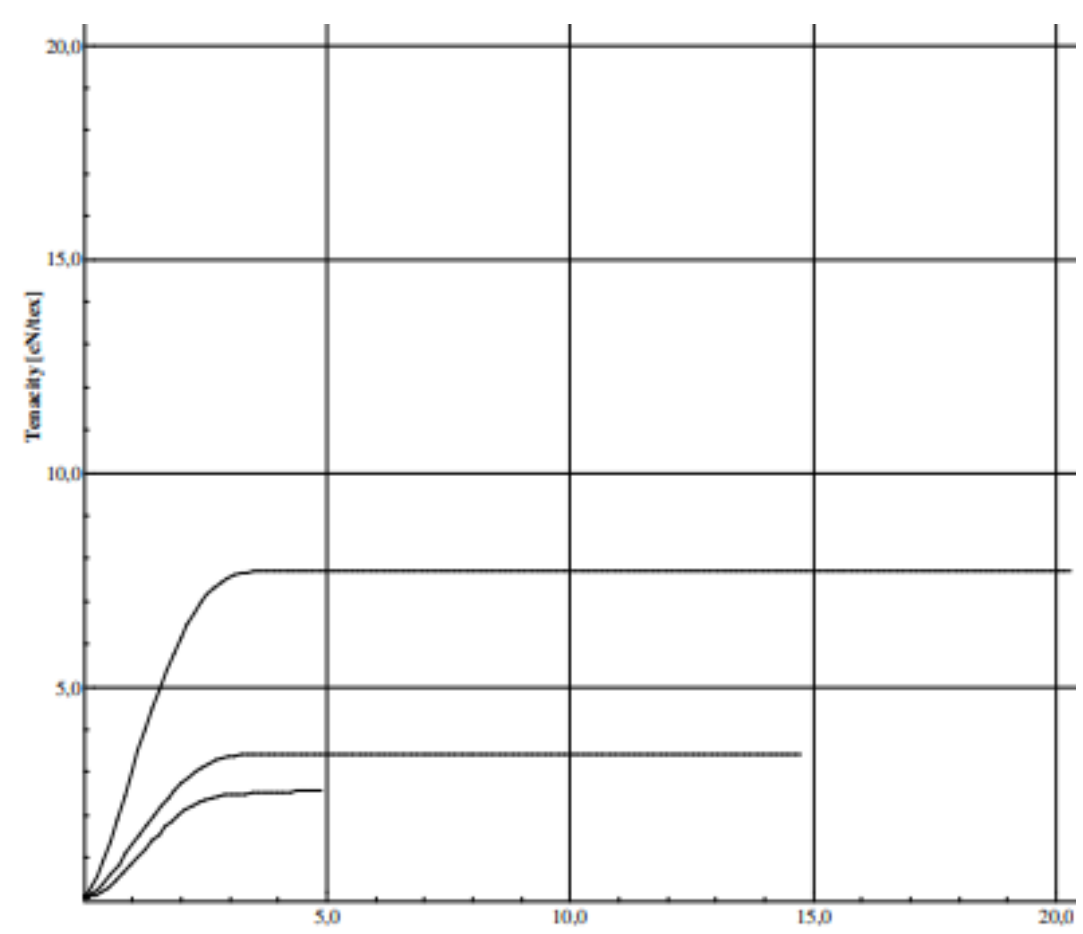


**Figure S47.** Stress-strain curves for **P(Q$_{35}$T$_{49}$F$_{16}$-H)** fibres.

## 8. Crosslinking/de-crosslinking investigation

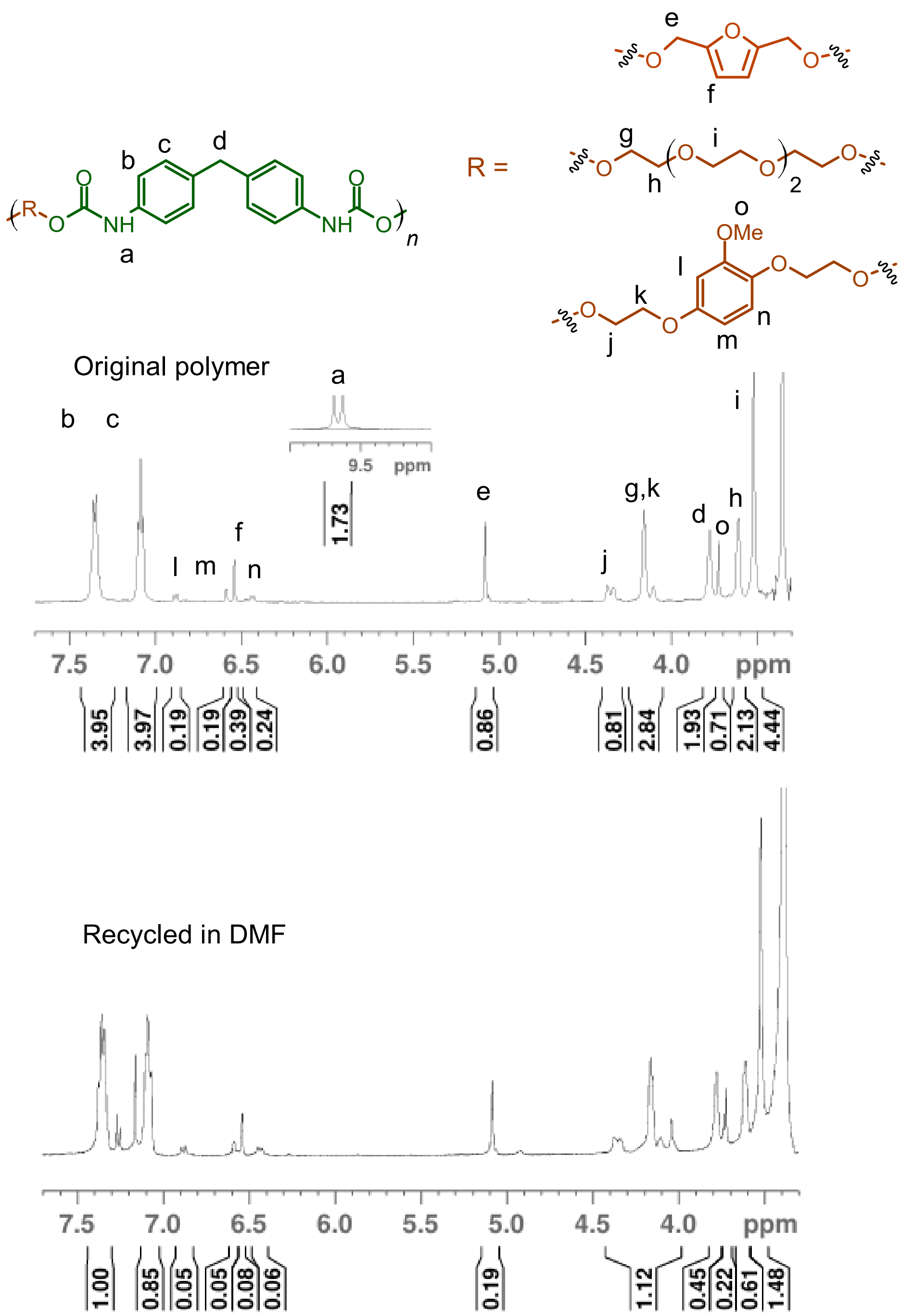


**Figure S48.** $^1$**H NMR spectra of P(Q$_{21}$T$_{58}$F$_{21}$-M)**

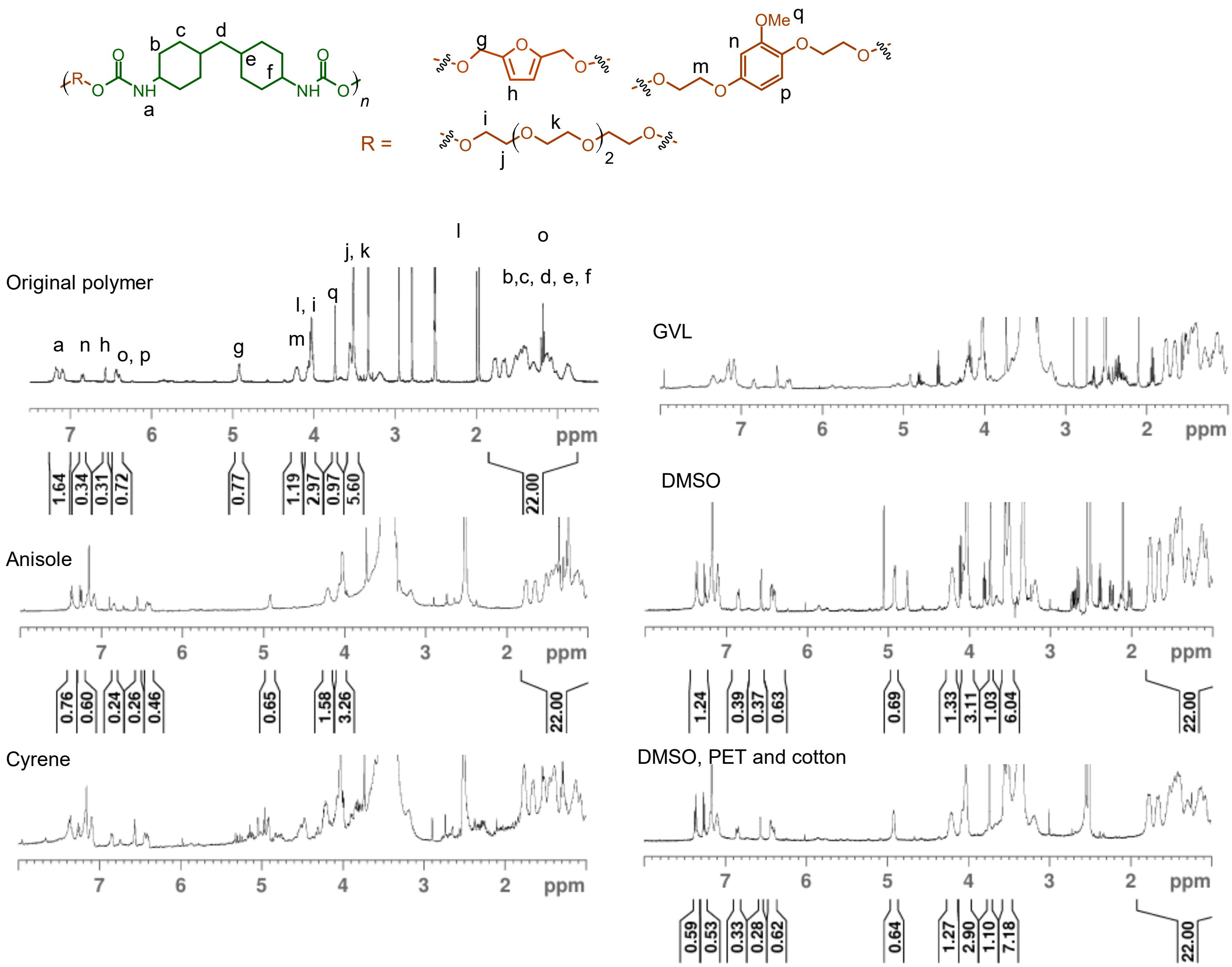


**Figure S49. $^{1}$H NMR spectra of P($Q_{35}T_{49}F_{16}$-H).**

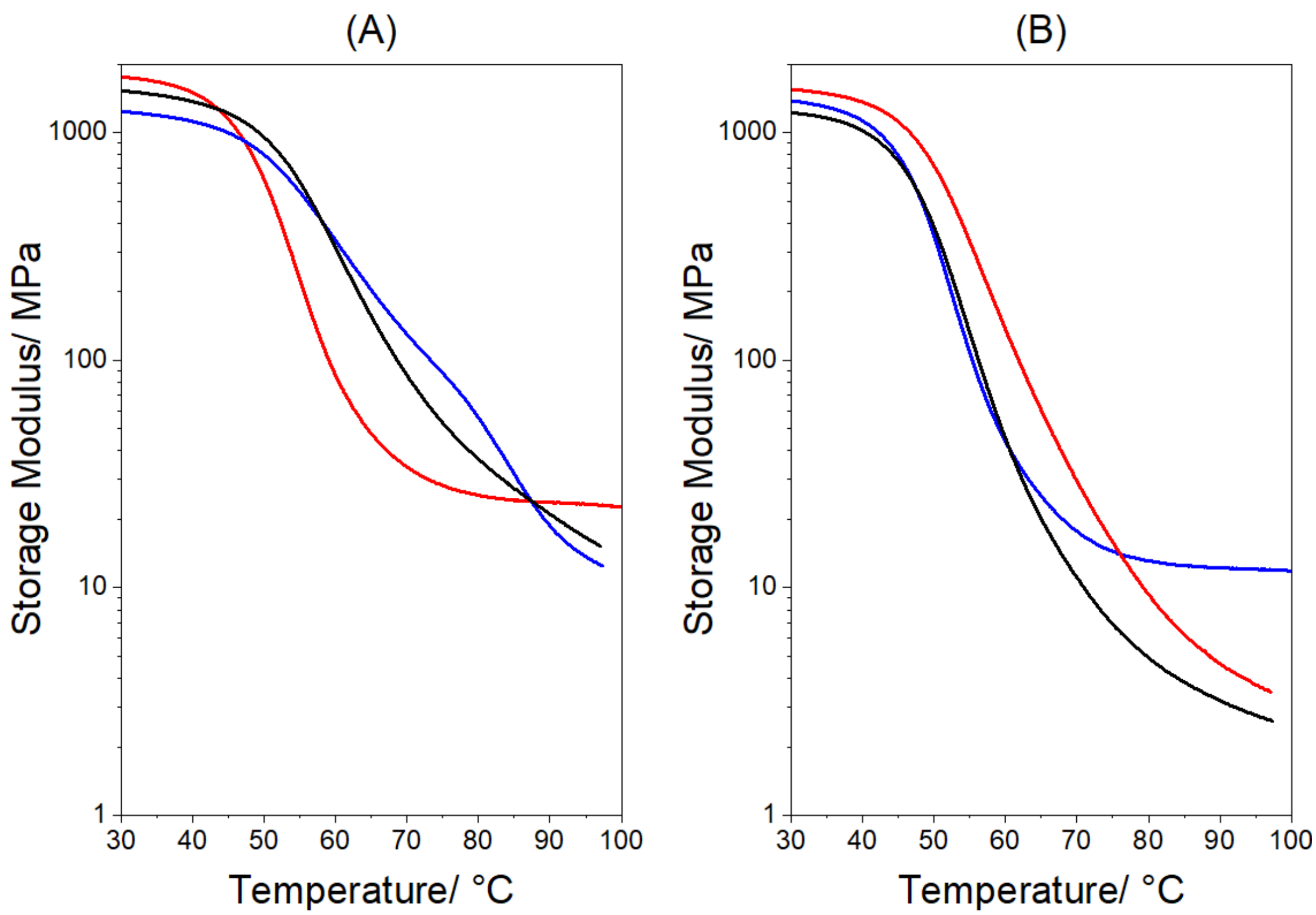


**Figure S50.** Storage modulus as a function of temperature according to DMA measurements for **(A) P($Q_{21}T_{58}F_{21}$-M)** and **(B) P($Q_{35}T_{49}F_{16}$-H)** after the first (black), second (red), and third (blue) crosslinking/de-crosslinking cycles.

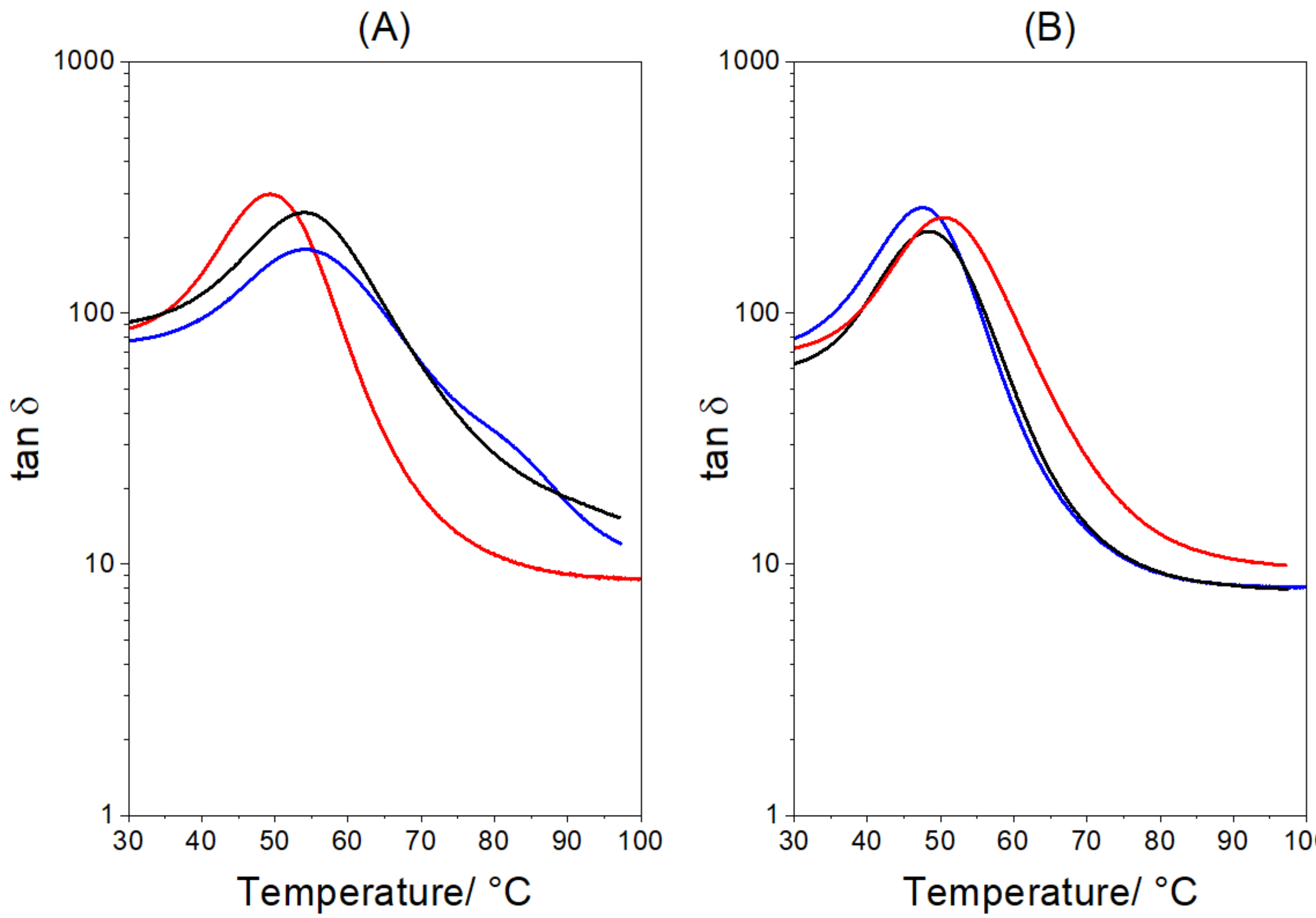


**Figure S51.** tan δ as a function of temperature obtained by DMA for **(A)** P($Q_{21}T_{58}F_{21}$-M) and **(B)** P($Q_{35}T_{49}F_{16}$-H) after the first (black), second (red), and third (blue) crosslinking/de-crosslinking cycles.
